# Recommendations for Accurate Resolution of Gene and Isoform Allele-Specific Expression in RNA-Seq Data

**DOI:** 10.1371/journal.pone.0126911

**Published:** 2015-05-12

**Authors:** David L. A. Wood, Katia Nones, Anita Steptoe, Angelika Christ, Ivon Harliwong, Felicity Newell, Timothy J. C. Bruxner, David Miller, Nicole Cloonan, Sean M. Grimmond

**Affiliations:** 1 Queensland Centre for Medical Genomics, University of Queensland, Brisbane, Australia; 2 QIMR Berghofer Medical Research Institute, 300 Herston Road, Herston, QLD, 4006, Australia; 3 Translational Research Centre, University of Glasgow, Glasgow, Scotland; Georgia Institute of Technology, UNITED STATES

## Abstract

Genetic variation modulates gene expression transcriptionally or post-transcriptionally, and can profoundly alter an individual’s phenotype. Measuring allelic differential expression at heterozygous loci within an individual, a phenomenon called allele-specific expression (ASE), can assist in identifying such factors. Massively parallel DNA and RNA sequencing and advances in bioinformatic methodologies provide an outstanding opportunity to measure ASE genome-wide. In this study, matched DNA and RNA sequencing, genotyping arrays and computationally phased haplotypes were integrated to comprehensively and conservatively quantify ASE in a single human brain and liver tissue sample. We describe a methodological evaluation and assessment of common bioinformatic steps for ASE quantification, and recommend a robust approach to accurately measure SNP, gene and isoform ASE through the use of personalized haplotype genome alignment, strict alignment quality control and intragenic SNP aggregation. Our results indicate that accurate ASE quantification requires careful bioinformatic analyses and is adversely affected by sample specific alignment confounders and random sampling even at moderate sequence depths. We identified multiple known and several novel ASE genes in liver, including *WDR72*, *DSP* and *UBD*, as well as genes that contained ASE SNPs with imbalance direction discordant with haplotype phase, explainable by annotated transcript structure, suggesting isoform derived ASE. The methods evaluated in this study will be of use to researchers performing highly conservative quantification of ASE, and the genes and isoforms identified as ASE of interest to researchers studying those loci.

## Introduction

A major goal of medical genomics is linking genetics with complex traits and diseases, and one mechanism by which genetic variability contributes to phenotype is through modulation of gene expression [1–3]. Small changes in gene expression can lead to profound phenotypic change, and may predispose an individual to disease [4–8]. Genetic variations can modulate transcription rate via epigenetic changes [9–12], through modification of transcription factor binding affinity [13], or post-transcriptionally by changing splicing patterns [14–17]. An effective method to assist in the identification of *cis* acting genetic factors is to study allelic imbalanced expression at heterozygous SNPs within an individual, a phenomenon commonly referred to as allele-specific expression (ASE). Identifying ASE can pinpoint causative and often low frequency disease associated variants [16]. ASE is a complex phenomenon caused by a variety of mechanisms including imprinting, X-chromosome inactivation and the above-mentioned transcriptional and post-transcriptional acting factors (for reviews, see references [18–20]).

Massively parallel RNA-sequencing [21,22] is well suited to study ASE as it provides nucleotide-resolution data, digital quantification of allele expression and comprehensive genome-wide coverage. Using this technology, multiple screens have been performed searching for ASE across lymphoblastoid cell lines and primary tissues [16,17,23–25]. Studies on the frequency of ASE genome-wide vary greatly, ranging from about 5% [26,27], to around 20% [28,29] and as high as 80% [30]. The reasons for these differences are a combination of genetic diversity, the tissues being studied, and also the informatics, statistics and classification of ASE, which has been shown to be particularly challenging [26,31,32]. Accurate quantification of ASE relies on sufficient sequence depth, sufficient heterozygosity, correct alignment with appropriate error control mechanisms, and comprehensive integrative expression analysis.

In this study, we performed a methodological evaluation of frequently used bioinformatic processes associated with ASE research. The method of aligning reads to personalized haplotype genomes [28] is confirmed in our results as highly beneficial for ASE analysis over universal genome alignment. SNP alignments are comprehensively interrogated and recommendations provided for removing SNPs from analyses that are likely to provide erroneous ASE predictions. In addition, we explore two surprisingly overlooked areas of ASE analysis, the affect of sampling on allele expression quantification, and concordance of allele expression imbalance direction among intragenic phased SNPs. Finally, we apply these methods to catalog ASE events in two human tissues, show how this method can identify known and novel ASE genes, and propose that the much of ASE we observe in these data is isoform-specific and most likely driven by complex transcriptional events, of which novel examples are provided.

## Results and Discussion

### Generation of a high-resolution, customized data set for ASE identification

To robustly interrogate the biases associated with ASE identification, we have generated high-resolution, high-quality data consisting of; (i) two technical replicates of total RNA sequencing (RNA-seq) from two human tissue samples with an average of 96.96 million uniquely mapped reads per sample; (ii) whole genome sequencing (WGS) from the same samples with an average coverage of 24.44, and (iii) matching genotyping arrays using the Illumina Omni Quad 1 million SNP chips.

Within a single sample, the power to detect ASE is determined by the number of sufficiently expressed heterozygous SNPs [16], and improved by incorporating haplotype phase information [30,33]. To maximize this power, we called SNPs from WGS data for each individual, then phased haplotypes using a subset of these SNPs independently verified by array genotyping (see Materials and Methods). This approach resulted in 10.27 and 10.29 million phased and sequence verified SNPs for the liver and brain samples respectively, of which 1.63 and 1.65 million were heterozygous and available for ASE testing if expressed (S1 Table). The proportion of heterozygosity of WGS verified SNPs in brain and liver was 16.03% and 15.86%, respectively (S1 Fig). These proportions were consistent (slightly lower than the mean) with all samples from the 1000G reference data set used for imputation. Performing both the SNP imputation and WGS provided a robust and comprehensive set of variants with which to evaluate ASE, more than six times the coverage of the genotype array. Obtaining the haplotype phase provided a framework on which to assist in reconstructing observed isoform-specific ASE events.

A small proportion of SNPs called from the WGS data were not present in the haplotype reference set (453,562 from brain and 423,364 from liver), and were excluded from ASE testing as they could not be phased. Additionally, 160,844 and 221,595 small insertions and deletions (indels) were called in the liver and brain samples respectively. Both these and the un-phased SNPs were utilized when interrogating alignment accuracy (see below).

### Diploid alignment improves sensitivity and specificity of SNP ASE classification

Previous studies have shown that aligning RNA-seq reads to personalised haplotype genomes and selecting the best alignments (hereon referred to as ‘diploid alignment’) largely eliminates ‘reference bias’ seen when aligning reads to the universal reference (‘universal alignment’) [26,28]. However, several recent studies have not adopted this methodology, instead relying on simulation studies to exclude SNPs identified as affected by reference bias [16,17]. We tested the effectiveness of diploid alignment, constructing personalized haplotype genome sequences for each individual from the phased high-confidence SNPs, against which we aligned the RNA-seq data. An optimal set of alignments was selected and written to BAM files using custom software available on request (see Materials and Methods). The resulting mapped data sets contained between 16.09 and 25.69 million PCR de-duplicated, uniquely aligned R1 reads (S2 Table). Gene expression values between library replicates showed strong technical reproducibility (S2 Fig).

To quantify the difference between diploid and universal alignment methods, we compared the number and allelic origin of reads aligning to high-confidence heterozygous SNPs. At SNPs with at least 10 uniquely mapped, PCR de-duplicated reads (referred to from here as ‘testable SNPs’), diploid alignment resulted in an additional 177,618 reads uniquely aligning (4.7% of all reads aligning across heterozygous SNPs, S3 Table). Of these additional reads, the vast majority (77.3%), were RNA transcribed from the variant allele. Diploid alignment resulted in a mean reference fraction of 0.503, much closer to the expected fraction of 0.5 and significantly different to universal alignment (mean reference fraction 0.516, *p-value* < 4.21e-77, student t-test, S3 Fig). Therefore, diploid alignment resulted in increased data yield, most of which was variant derived, and more balanced allelic expression, consistent with previous observations [26,28].

**Fig 1:**
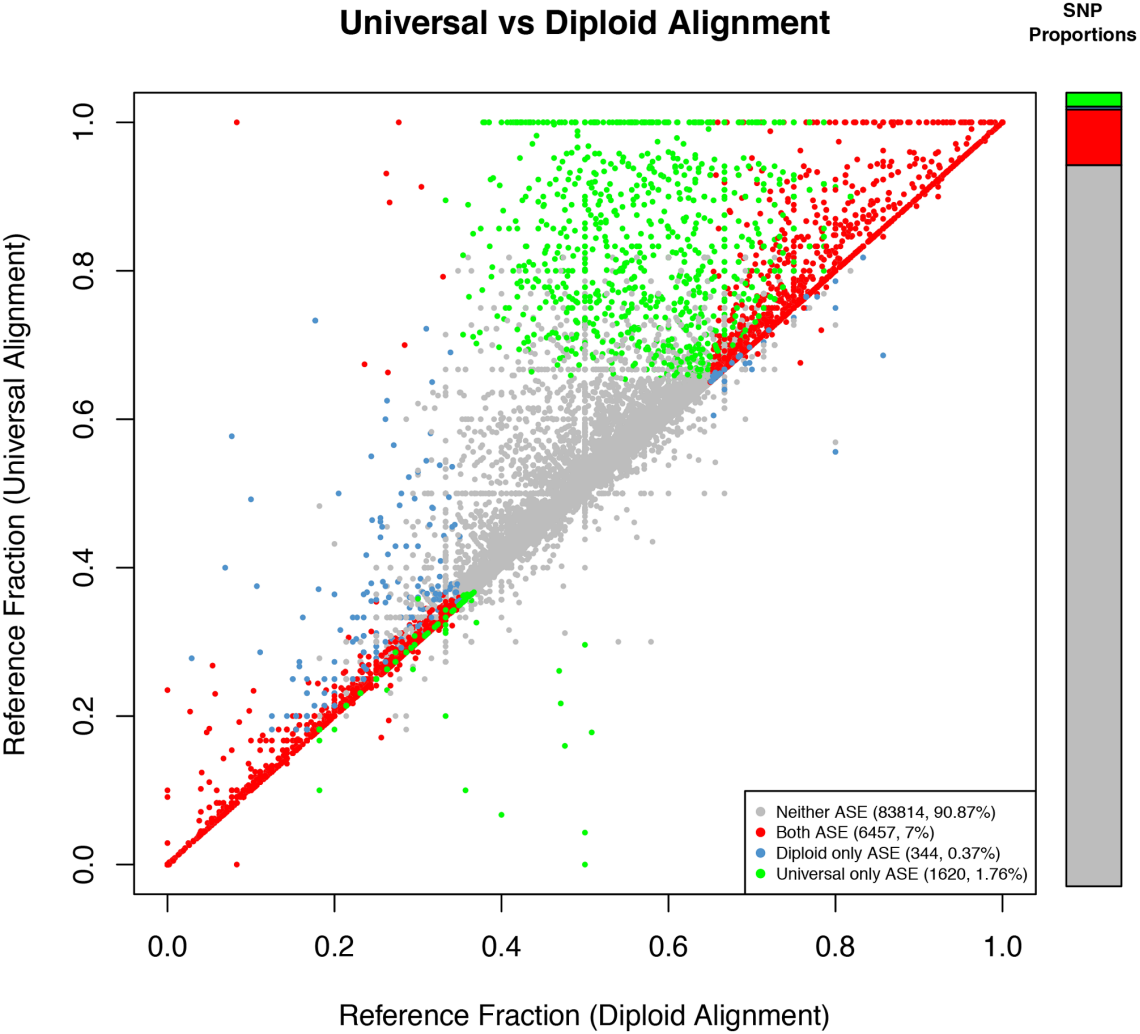
SNP reference fraction correlations between universal and diploid alignment methods. Each point is a SNP, coloured according to its ASE classification in both alignment methods. Bar plots indicate the proportion of all SNPs in the plot (left), and the proportion of all SNPs classified as ASE in any method (right). Aligning RNA-seq reads to personalised haplotype references then choosing the alignments with the smallest edit-distance resulted in more balanced allelic expression ratios than aligning to the universal reference.

Allelic expression imbalance is a quantitative not qualitative trait, but in order to survey it across tissues, we categorized each SNP as ASE or not using both the observed reference fraction and the probability of this reference fraction (see Materials and Methods for details). Using both criteria ensured that neither highly expressed SNPs with slight imbalance, nor lowly expressed SNPs with large imbalance were classified as ASE, resulting in a conservative set of ASE SNPs. In total, more SNPs were classified ASE using universal alignment (8.76%; 8,082 out of 92,250) than diploid alignment (7.45%; 7,177 out of 96,658; S4 Table). For SNPs that were testable in both methods (n = 92,235), 23.25% changed reference fraction between the diploid and universal alignment sets, indicating that at least one alignment at those loci differed between the methods. More than two percent of SNPs showed substantial enough reference fraction changes to force an ASE reclassification, resulting in 344 more ASE SNPs and 1,620 less ASE SNPs in the diploid alignment set compared to the universal alignment set (Fig 1). These results indicate that not only did diploid alignment reduce global reference bias, but it had the highly desirable effect of increasing the number of testable SNPs (higher sensitivity), and reducing the extent of alignment bias and therefore false positive ASE classifications (higher specificity).

### Proximal SNVs and poor alignability cause false positive ASE predictions

**Fig 2:**
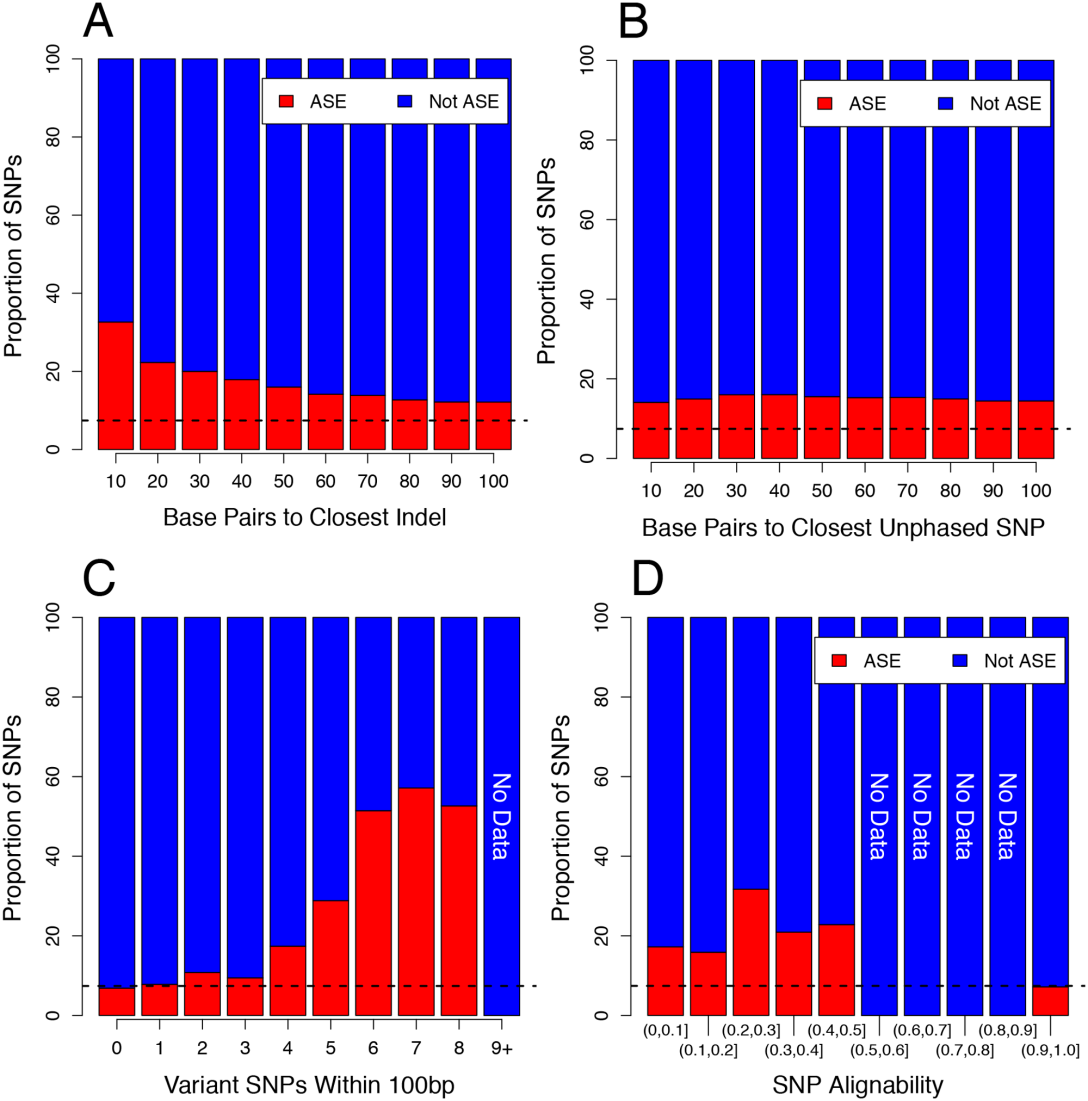
SNPs proximal to SNVs are more likely to be ASE. (A) SNPs binned by genomic distance to nearest heterozygous indel. (B) SNPs binned by nearest heterozygous un-phased *de novo* called SNP. (C) SNPs binned by the number of proximal heterozygous SNPs. (D) SNPs binned by genomic alignability. The closer a SNP is to a heterozygous SNV, the more likely that SNP is to be classified as ASE. For indels, this effect is stronger than SNPs, which are only slightly affected, however as the number of SNPs within a read length (100bp) increases, this effect is increased. In all plots, the dashed line represents the average ASE proportion for all SNPs. Numbers at the top of the bars indicate the number of SNPs in each bin (blue not ASE, red ASE).

Small insertions and deletions (indels) and SNPs, collectively termed small nucleotide variations (SNVs) not included in the construction of the personalised haplotype references may cause additional alignment errors and false positive ASE calls. To evaluate the extent of this, SNPs were binned by the genomic distance to their nearest un-phased SNV and the proportion of ASE SNPs in each bin calculated. SNPs with an indel very close (less than 10bp) were much more likely to be classified ASE (32.63% of SNPs, n = 95, Fig 2a) than the average likelihood of 7.43%. Extending further, all SNPs within the length of a read (100bp) of an un-phased SNV were more likely to be classified as ASE than SNPs with distal indels. SNPs with proximal un-phased *de novo* called SNPs were less affected, and only displayed a marginal increase above average ASE classification proportion (Fig 2b). SNPs with multiple other SNPs in close proximity, however, displayed greater likelihood of ASE classification (Fig 2c). These results are consistent with recently reported analyses showing an increased proportion of ASE SNPs proximal to SNVs [16,32]. However, in addition to this, we also observed an increase in positive ASE classification for SNPs located up to 10kb from SNVs (S4 Fig). This observation may be due to spliced reads spanning larger genomic distances and therefore distal error causing SNVs, or evidence of increased ASE in polymorphic regions, possible given expression quantitative trait loci (eQTLs) can be causative of *cis* expression modulation up to 2Mb away from their target genes [34].

In addition to assessing alignment artifacts, we also quantified the affect read alignability had on SNP ASE classification. Due to sequence similarity, some genomic regions are more difficult to align than others [35,36], and reads derived from these regions are more likely to map incorrectly. In such regions, if alignment errors occur, we expect a higher number of false positive ASE predictions. To test this, we calculated the proportion of ASE SNPs binned by alignability values obtained from the Encode 100bp alignability track [36]. SNPs with lower than perfect alignability (<1) showed a higher likelihood of being classified as ASE than SNPs with perfect alignability (Fig 2d), suggesting that these SNPs were likely subject to erroneous ASE predictions caused by incorrect or missing alignments.

Taken together, these SNP alignment tests provided a comprehensive assessment of the susceptibility of our ASE predictions to alignment error. Very few SNPs were affected by more than one alignment confounder, and surprisingly SNPs with the largest changes in reference fraction between diploid and universal alignment methods were mostly unaffected by these potential alignment error causing artifacts (S5 Fig). These results indicate that comprehensive alignment quality control, in addition to the diploid alignment method, is highly desirable to improve the coverage and accuracy of ASE predictions and should be adopted when possible. To obtain a high confidence set of SNPs for further analysis, we used the diploid alignments, and removed SNPs with either an indel or four or more un-phased SNPs within 100bp, as well as SNPs with less-than-perfect alignability. Across both tissues, 1,177, 397 and 923 SNPs fell into each of these categories respectively. In total, 2,450 SNPs testable for ASE were removed (1,000 from the brain sample, and 1,450 from liver, S5 Table). Full tables of all testable SNPs across all replicates are provided in S1 Dataset.

### Low R^2^ values do not substantially confound ASE prediction

Although our use of WGS for SNP verification meant that it was unnecessary to rely on R^2^ values as a measure of imputation quality, we did observe an increase in the proportion of ASE called SNPs with lower R^2^ values (from 6.9% when R^2^ > 0.9 to 21.8% when R^2^ < 0.2, S6a Fig). It was therefore important to more closely examine the R^2^ values of our imputed SNPs to exclude a potential source of bias in our results. As might be expected, SNPs with the lowest R^2^ values (<0.1) made up the majority of the SNPs failing WGS verification (314,995 of 675,687 in total), however SNPs across the entire range of R^2^ values failed verification, including 98,163 SNPs with R^2^ values ≥ 0.9 (S6c Fig). Proportionally, SNPs with high R^2^ values had the highest WGS verification rate (S6d Fig). SNPs with low R^2^ values were proportionally depleted of both WGS failed and WGS verified heterozygous SNPs relative to SNPs with higher R^2^ values (S6e and S6f Fig).

Looking specifically at SNPs imputed as heterozygous, the observed fraction of reads matching the reference allele at these SNPs for all SNPs passing WGS verification were centered around 0.5, a result consistent with genuine heterozygosity (S7a Fig). SNPs failing verification have reference fractions mostly around 1.0 or 0.0, and are most likely homozygous SNPs incorrectly called heterozygous (S7b Fig). We note an enrichment for SNPs with R^2^ values of 1.0, but DNA reference fractions of around 0.8 or 0.2 as failing WGS verification (S7b Fig). Many of these SNPs are most likely heterozygous, but excluded from our analysis due to insufficient coverage for statistically reliable SNP calling. We next plotted the correlation between R^2^ values and the RNA-seq reference fraction for all testable SNPs. No specific bias towards low R^2^ values was observed—SNPs with low R^2^ values show the same range of reference fraction values than SNPs with high R^2^ values (S7c Fig).

Finally, we re-plotted the proportion of ASE called SNPs after filtering for proximal SNVs and poor alignability (see previous section), and found that the bias towards low R^2^ values was substantially reduced (from 21.8% to 11.4% when R^2^ < 0.2, S6b Fig). As there was still a small bias, we repeated the analyses investigating the proportion of ASE SNPs proximal to SNVs and within specific transcript features using only perfect proxies (R^2^ = 1) to determine whether the inclusion of SNPs with a lower R^2^ value has confounded our results. The proportions of SNPs classified as ASE reduced for those proximal to SNVs and in regions of poor alignability, but all proportions were still above the average ASE rate genome-wide (S8 and S9 Figs). These results show that even when using SNPs with perfect R^2^ values, alignment confounders still affect ASE quantification and SNPs proximal to such confounders should be removed. Together, these results confirm that WGS verification and subsequent filtering of imputed SNPs allows for a sensitive examination of ASE without substantial bias from the inclusion of SNPs with low R^2^ values.

### Sampling Confounds ASE Prediction

**Fig 3:**
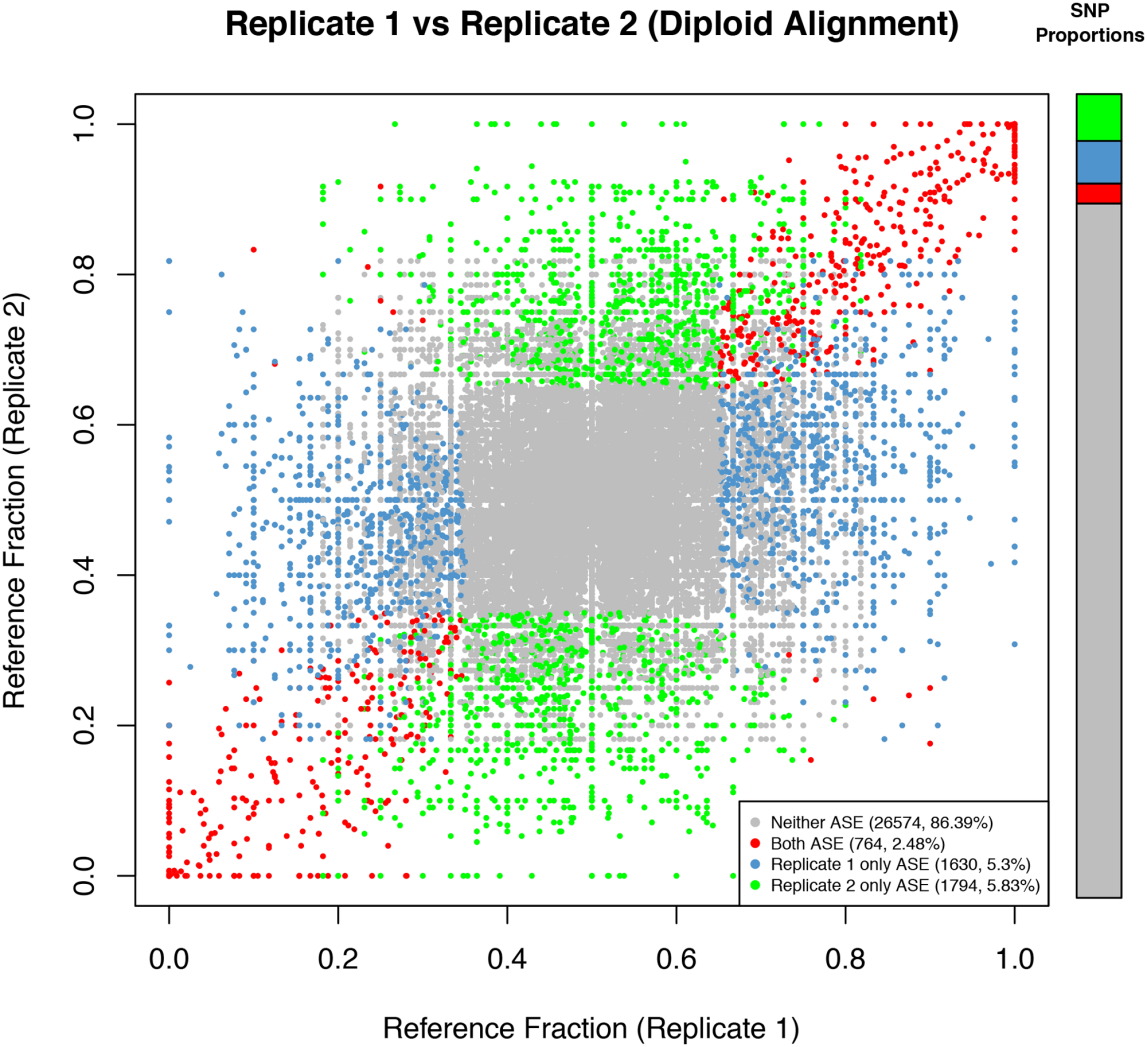
Reference fraction correlations between replicates using diploid alignment. Each point is a SNP, coloured according to its ASE classification in both replicates. Bar plots indicate the proportions of all SNPs in the plot (left), and ASE SNPs (right). Calculating ASE with low read counts can result in sampling being a confounder.

Previous studies have relied on testing ASE at SNPs using depths as shallow as six reads [28] but routinely at a depth of 20 [27,29]. At these depths, ASE quantification is susceptible to substantial sampling variability causing skewed reference fraction predictions (S10 Fig). Sampling effects in this study were quantified by comparing ASE SNP classifications between technical replicates. Of all pairs of testable SNPs, 86.39% were classified as not ASE in both replicates. Of the remaining 13.61%, 18.24% were classified as ASE in both replicates (2.48% of all SNPs, from here referred to as ‘replicated ASE SNPs’, Fig 3 and S6 Table). The remaining SNPs were split relatively evenly between replicates (38.92% classified as ASE in replicate one and 42.84% in replicate two). While the reference fractions and ASE classification of SNPs were not strongly correlated across these data, the sequence depth measured at each of these testable SNPs was strongly correlated (S11 Fig).

We next tested whether increasing the minimum read depth to test a SNP for ASE would improve correlations between replicates. Setting the minimum read depth to 50 reads increased the overlap of ASE SNP classifications to 40.80%, and raising it to 100 or more reads increased the overlap to 65.15%, but resulted in a massive loss of testable SNPs (from 30,762 at 10+ reads, to 1,868 at 100+ reads, S6 Table and S12 Fig), representing an unacceptable loss of testable SNPs. Sampling effects on allele expression is even greater than sampling effects for biallelic gene and isoform expression due to the second degree of freedom introduced by the additional variable (allele) to be tested, and because on average the actual amount of data available for allele expression quantification is half that for gene expression quantification. These results indicate that sampling can be a major confounder in ASE quantification, even at moderate depths, and render important the use of SNP aggregation methods (described below), replication or independent verification when measuring ASE.

### SNP aggregation by phased haplotype reduces sampling affects

Having collated a high confidence set of replicated and alignment quality controlled SNPs, we next sought to evaluate the extent of ASE at a whole-locus level by SNP aggregation. All testable and alignment quality controlled SNPs within annotated Ensembl v71 genic boundaries were collated and genic ASE calculated using a binomial test on the sum of the haplotype read counts. This method accommodates sampling effects observed at individual SNPs as the summed counts have substantially more power, as well as quantifies allelic imbalance with respect to haplotype, and provides a mechanism to rank ASE genes by magnitude of imbalance. Each gene was annotated with the presence of multiple testable exonic SNPs, replicated ASE SNPs, and concordant direction of allelic imbalance with the phased haplotype. Taken together, genes could then be ranked and interrogated for maximum accuracy or sensitivity.

Across both tissues, of the 3,843 genes with at least two testable exonic SNPs in both replicates, 143 contained at least one replicated ASE SNP, and all SNPs showed concordant imbalance direction in 43 of these (S1 Dataset). Ensuring a gene contained at least one replicated ASE SNP dramatically increased the overlap of ASE genes between technical replicates and hence the specificity of our candidate lists (S13 Fig). However, removing this imbalance concordance requirement increased the number of genes classified as ASE in both replicates from 69.1% to 73.2%. This is to be expected, as sampling variability will affect SNPs causing the allelic fraction to skew and hence the test for phase concordance is not as robust as the genic test of ASE. This observation is also biologically meaningful, as genetic differences have been shown to differentially affect intragenic exon expression [16]. We note also that of all SNPs testable in both replicates (n = 30,762), 13.47% were testable in both tissues (n = 2,072 for each tissue). Of these SNPs, only eight were classified as ASE in both liver and brain, possibly indicating high tissue-specificity for ASE SNPs (S14 Fig).

### A recommendation on methodology for ASE quantification

**Fig 4:**
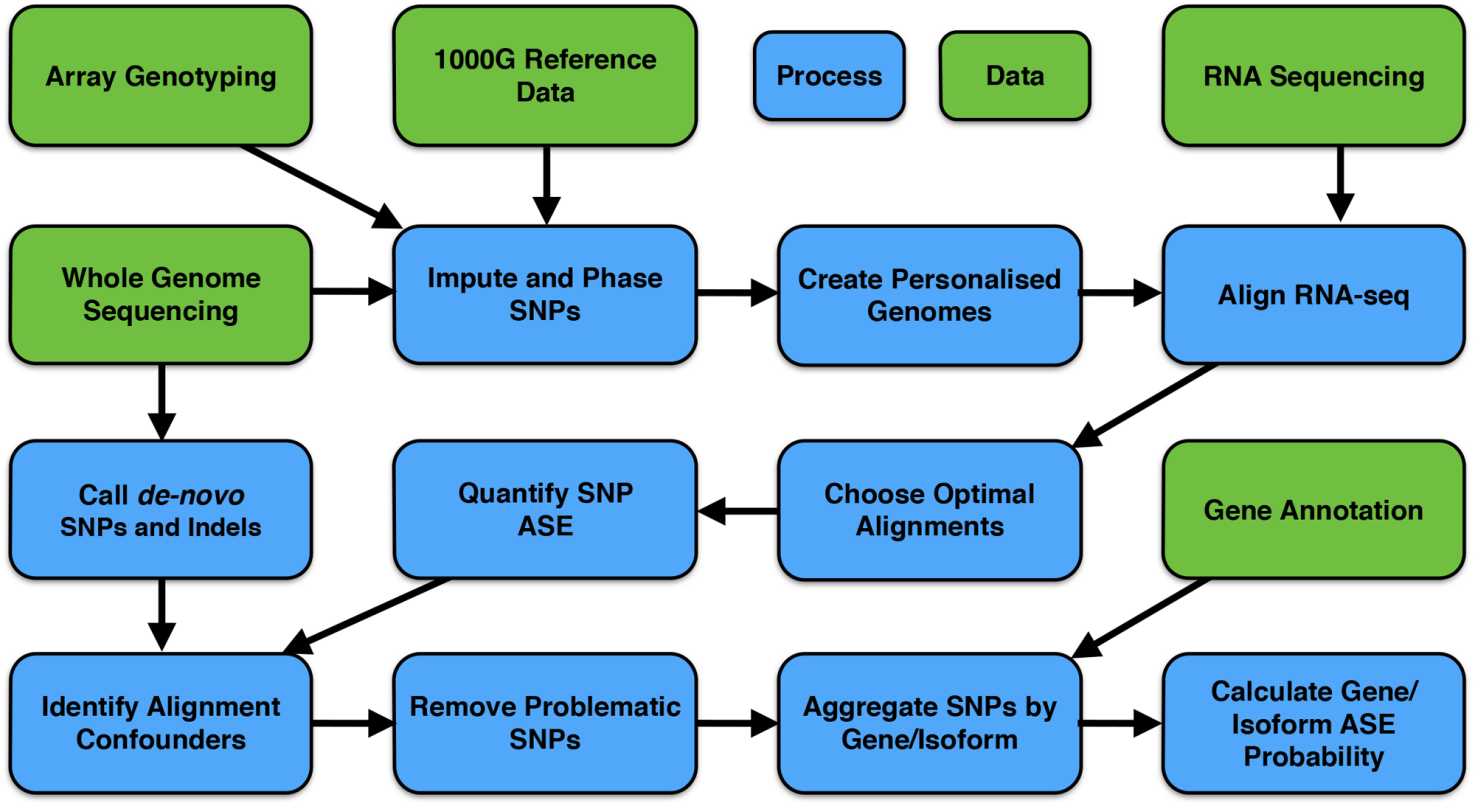
Recommended workflow for ASE quantification using matched genotype and RNA-seq data. Robust quantification of ASE requires detailed genotype information and matched high quality RNA-seq data. Phasing and imputing SNPs provides haplotype information, used to determine whether intragenic SNPs with imbalanced expression show concordant direction of imbalance, as well as build personalised reference genomes for alignment. Personalised alignment increases both sensitivity (data yield) and specificity of ASE quantification. WGS data can be used to identify indels and other proximal SNVs not included in the imputation reference data set that may confound alignment and potentially produce false positive ASE calls. SNPs proximal to such factors can be removed from further analysis. Aggregation of SNPs by gene and isoform boundaries reduces potential sampling issues for lowly expressed regions, and assists in identifying ASE regions (genes and isoforms).

As described above, quantification of ASE using RNA-seq data can involve a series of bioinformatic steps not typically performed when quantifying standard gene or isoform expression. These include nucleotide specific integration of genotype and haplotype phase data with RNA-seq, personalised sequence alignment [26,28], alignment quality control [32], and intragenic SNP aggregation [30] (Fig 4). Performing all of these steps will provide a robust and conservative set of putative ASE SNPs and genomic regions (genes, isoforms or regions). When sample sizes are small, and high specificity is required for ASE analyses, we suggest using all of these methods. The following sections describe the genes and isoforms identified as ASE in the liver and brain samples.

### Description of known and novel ASE genes

**Fig 5:**
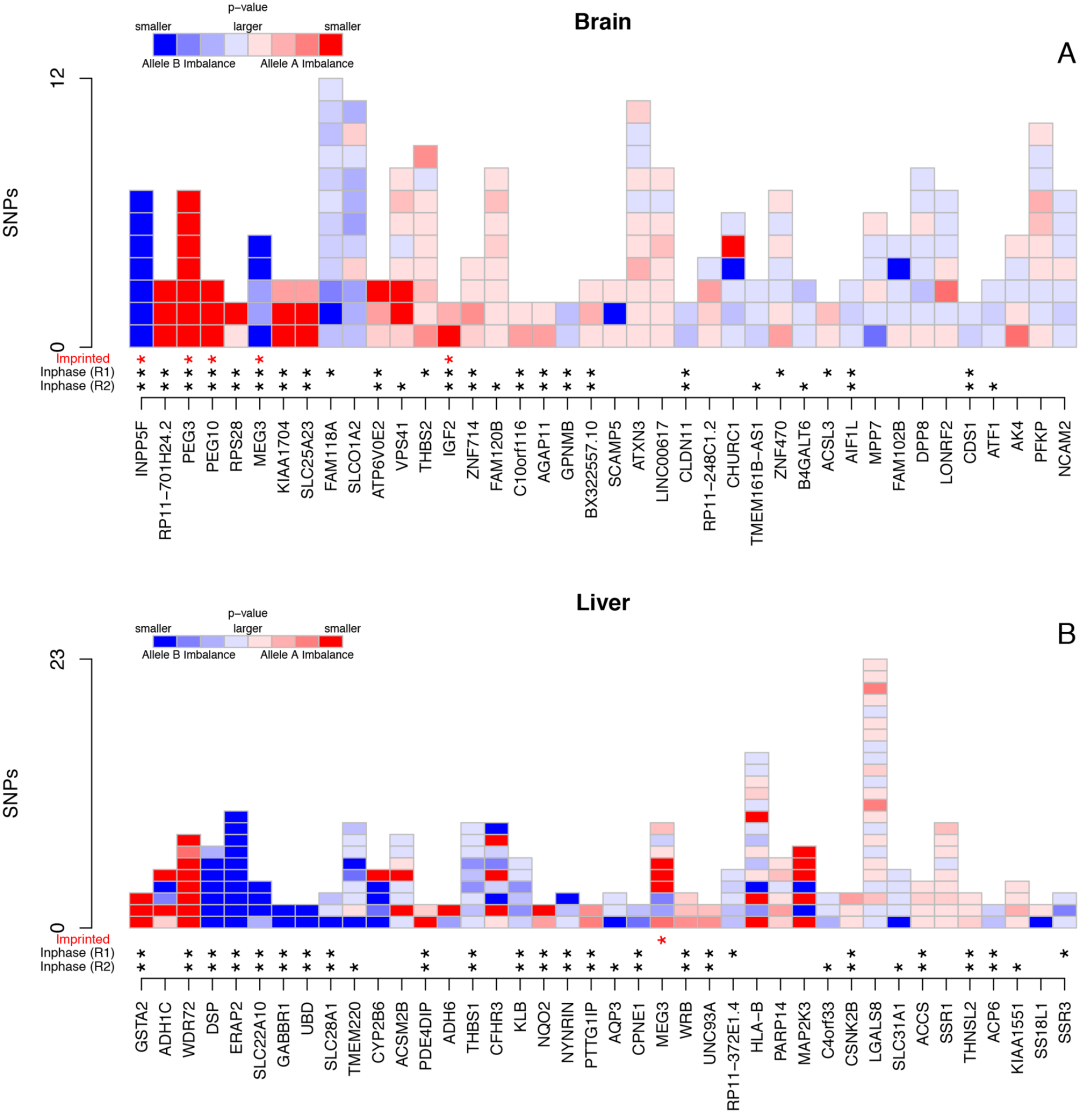
Highest ranked ASE genes from (A) brain and (B) liver. Each gene is shown as vertical bars of exonic SNPs testable within that gene. Red boxes are imbalanced towards haplotype A, and blue boxes imbalanced towards haplotype B, darker colours indicate more significant the level of imbalance. All data presented is the average of the two replicates. Genes are ordered left to right by the probability of gene imbalance. Stars below the bars indicate whether the gene is known imprinted (red), or whether the SNPs for that gene all show concordant direction of expression imbalance in each replicate (black). Each of the tissues has a cluster of genes with very significant SNPs mostly with concordant direction of ASE towards the left of the plots (strong genic ASE). It is clear, however, that some genes contain SNPs that vary in their direction and magnitude of imbalance.

The two tissues in this study were selected for the high frequency of imprinting in brain (reviewed in [37]), and the high amount of known genetic modulation of gene expression within metabolically active liver [38]. The 40 genes showing the strongest evidence for ASE are shown in Fig 5 and Table 1 and Table 2. Our method is confirmed by identification of known imprinted genes among the top ASE brain genes (*PEG3*, *PEG10*, *INPP5F*, *MEG3*, *IGF2*), and also in liver (*MEG3*). Imbalanced expression was also detected in liver at the ERAP2 locus, which harbors a known splicing quantitative trait loci (sQTL) introducing a premature stop codon, resulting in allele-specific nonsense mediated decay [39] (S15a Fig). Our assay detected the gene *GSTA2* with very strong ASE in liver (S15b Fig). *GSTA2* is highly expressed in this tissue, and has well characterised genetically modulated expression variability that affects an individuals ability to detoxify carcinogen metabolites and efficacy to chemotherapeutic drugs [40].

**Table 1:** Top ASE genes from the brain tissue sample.

Ensembl Gene ID	Gene Name	Imbalance P-value	Rep1 In-phase	Rep2 In-phase	Imprinting Status^a^	eQTLs^b^	transcript-QTLs^b^	exon-QTLs^b^
**ENSG00000198825**	*INPP5F*	0	YES	YES	Paternal	0	0	0
**ENSG00000257151**	*RP11-701H24*.*2*	0	YES	YES	Not reported	0	0	0
**ENSG00000198300**	*PEG3*	4.94E-324	YES	YES	Paternal	0	0	0
**ENSG00000242265**	*PEG10*	2.16E-211	YES	YES	Paternal	0	0	0
**ENSG00000233927**	*RPS28*	3.40E-168	YES	YES	Not reported	1	0	0
**ENSG00000214548**	*MEG3*	5.32E-110	YES	YES	Maternal	21	0	0
**ENSG00000133114**	*KIAA1704*	5.25E-44	YES	YES	Not reported	3	0	1
**ENSG00000125648**	*SLC25A23*	1.46E-30	YES	YES	Not reported	2	0	0
**ENSG00000100376**	*FAM118A*	4.63E-30	YES	No	Not reported	67	0	0
**ENSG00000084453**	*SLCO1A2*	9.30E-28	No	No	Not reported	0	0	0
**ENSG00000171130**	*ATP6V0E2*	1.94E-25	YES	YES	Not reported	1	0	0
**ENSG00000006715**	*VPS41*	5.80E-21	No	YES	Not reported	33	0	4
**ENSG00000186340**	*THBS2*	6.24E-17	YES	No	Not reported	0	0	0
**ENSG00000167244**	*IGF2*	1.40E-15	YES	YES	Paternal	1	0	0
**ENSG00000160352**	*ZNF714*	6.24E-13	YES	YES	Not reported	0	0	0
**ENSG00000112584**	*FAM120B*	9.85E-10	No	YES	Not reported	1	1	0
**ENSG00000148671**	*C10orf116*	2.40E-08	YES	YES	Not reported	2	0	0
**ENSG00000151303**	*AGAP11*	2.40E-08	YES	YES	Not reported	0	0	0
**ENSG00000136235**	*GPNMB*	2.08E-07	YES	YES	Not reported	53	0	0
**ENSG00000215447**	*BX322557*.*10*	4.59E-07	YES	YES	Not reported	0	0	0
**ENSG00000198794**	*SCAMP5*	5.17E-07	No	No	Not reported	29	0	0
**ENSG00000066427**	*ATXN3*	7.13E-07	No	No	Not reported	23	0	0
**ENSG00000250366**	*LINC00617*	7.51E-06	No	No	Not reported	0	0	0
**ENSG00000013297**	*CLDN11*	7.87E-06	YES	YES	Not reported	1	0	0
**ENSG00000232229**	*RP11-248C1*.*2*	1.07E-05	No	No	Not reported	0	0	0
**ENSG00000258289**	*CHURC1*	1.07E-05	No	No	Not reported	133	0	0
**ENSG00000247828**	*TMEM161B-AS1*	2.47E-05	No	YES	Not reported	0	0	0
**ENSG00000197016**	*ZNF470*	9.58E-05	YES	No	Not reported	0	0	0
**ENSG00000118276**	*B4GALT6*	2.22E-04	No	YES	Not reported	0	0	0
**ENSG00000123983**	*ACSL3*	3.82E-04	YES	No	Not reported	8	0	3
**ENSG00000126878**	*AIF1L*	4.17E-04	YES	YES	Not reported	0	0	0
**ENSG00000150054**	*MPP7*	8.60E-04	No	No	Not reported	0	0	0
**ENSG00000162636**	*FAM102B*	1.12E-03	No	No	Not reported	0	0	0
**ENSG00000074603**	*DPP8*	1.96E-03	No	No	Not reported	0	0	5
**ENSG00000170500**	*LONRF2*	6.12E-03	No	No	Not reported	0	0	0
**ENSG00000163624**	*CDS1*	1.41E-02	YES	YES	Not reported	0	0	0
**ENSG00000123268**	*ATF1*	2.05E-02	No	YES	Not reported	0	0	0
**ENSG00000162433**	*AK4*	2.08E-02	No	No	Not reported	0	0	0
**ENSG00000067057**	*PFKP*	2.62E-02	No	No	Not reported	10	1	5
**ENSG00000154654**	*NCAM2*	3.47E-02	No	No	Not reported	0	0	0

^a^ Imprinting status sourced from http://www.geneimprint.org/ accessed December 4, 2013.

^b^ eQTL, transcript-QTL and exon-QTL data sourced from http://eqtl.uchicago.edu/cgi-bin/gbrowse/eqtl/, accessed January 29, 2014.

**Table 2:** Top ASE genes from the liver tissue sample.

Ensembl Gene ID	Gene Name	Imbalance P-value	Rep1 In-phase	Rep2 In-phase	Imprinting Status^a^	eQTLs^b^	transcript-QTLs^b^	exon-QTLs^b^
ENSG00000244067	*GSTA2*	0	Yes	Yes	Not Reported	15	0	0
ENSG00000248144	*ADH1C*	1.73E-186	No	No	Not Reported	0	0	0
ENSG00000166415	*WDR72*	4.47E-162	Yes	Yes	Not Reported	0	0	0
ENSG00000096696	*DSP*	9.58E-153	Yes	Yes	Not Reported	0	0	0
ENSG00000164308	*ERAP2*	3.34E-145	Yes	Yes	Not Reported	5	47	50
ENSG00000184999	*SLC22A10*	5.86E-105	Yes	Yes	Not Reported	1	0	0
ENSG00000204681	*GABBR1*	5.70E-75	Yes	Yes	Not Reported	0	0	0
ENSG00000213886	*UBD*	5.70E-75	Yes	Yes	Not Reported	1	0	0
ENSG00000156222	*SLC28A1*	2.10E-67	Yes	Yes	Not Reported	0	0	0
ENSG00000187824	*TMEM220*	3.65E-41	No	Yes	Not Reported	1	0	0
ENSG00000197408	*CYP2B6*	3.88E-40	No	No	Not Reported	0	0	0
ENSG00000066813	*ACSM2B*	6.32E-32	No	No	Not Reported	0	0	0
ENSG00000178104	*PDE4DIP*	1.05E-31	Yes	Yes	Not Reported	0	0	0
ENSG00000172955	*ADH6*	2.04E-27	No	No	Not Reported	0	0	0
ENSG00000137801	*THBS1*	1.07E-26	Yes	Yes	Not Reported	9	0	0
ENSG00000116785	*CFHR3*	8.13E-22	No	No	Not Reported	0	0	0
ENSG00000134962	*KLB*	8.24E-21	Yes	Yes	Not Reported	2	0	0
ENSG00000124588	*NQO2*	2.00E-16	Yes	Yes	Not Reported	49	0	0
ENSG00000205978	*NYNRIN*	4.96E-16	Yes	Yes	Not Reported	0	0	0
ENSG00000183255	*PTTG1IP*	2.06E-15	Yes	Yes	Not Reported	23	0	0
ENSG00000165272	*AQP3*	3.90E-14	No	Yes	Not Reported	1	0	0
ENSG00000214078	*CPNE1*	7.05E-13	Yes	Yes	Not Reported	167	23	46
ENSG00000214548	*MEG3*	1.53E-12	No	No	Maternal	21	0	0
ENSG00000182093	*WRB*	1.09E-11	Yes	Yes	Not Reported	38	0	0
ENSG00000112494	*UNC93A*	9.07E-11	Yes	Yes	Not Reported	0	0	0
ENSG00000243818	*RP11-372E1*.*4*	1.30E-10	Yes	No	Not Reported	0	0	0
ENSG00000234745	*HLA-B*	2.16E-10	No	No	Not Reported	11	0	0
ENSG00000173193	*PARP14*	1.90E-09	No	No	Not Reported	1	0	0
ENSG00000034152	*MAP2K3*	4.57E-09	No	No	Not Reported	0	0	0
ENSG00000151470	*C4orf33*	8.88E-09	No	Yes	Not Reported	2	0	0
ENSG00000204435	*CSNK2B*	2.81E-08	Yes	Yes	Not Reported	8	0	0
ENSG00000116977	*LGALS8*	5.35E-08	No	No	Not Reported	25	0	0
ENSG00000136868	*SLC31A1*	2.25E-07	No	Yes	Not Reported	1	0	0
ENSG00000110455	*ACCS*	2.45E-07	Yes	Yes	Not Reported	3	0	0
ENSG00000124783	*SSR1*	4.30E-07	No	No	Not Reported	1	0	0
ENSG00000144115	*THNSL2*	4.76E-07	Yes	Yes	Not Reported	3	0	0
ENSG00000162836	*ACP6*	9.70E-07	Yes	Yes	Not Reported	9	0	0
ENSG00000174718	*KIAA1551*	4.49E-06	No	Yes	Not Reported	0	0	0
ENSG00000184402	*SS18L1*	5.66E-06	No	No	Not Reported	1	0	0
ENSG00000114850	*SSR3*	3.84E-05	Yes	No	Not Reported	3	0	0

^a^ Imprinting status sourced from http://www.geneimprint.org/ accessed December 4, 2013.

^b^ eQTL, transcript-QTL and exon-QTL data sourced from http://eqtl.uchicago.edu/cgi-bin/gbrowse/eqtl/, accessed January 29, 2014.

**Fig 6:**
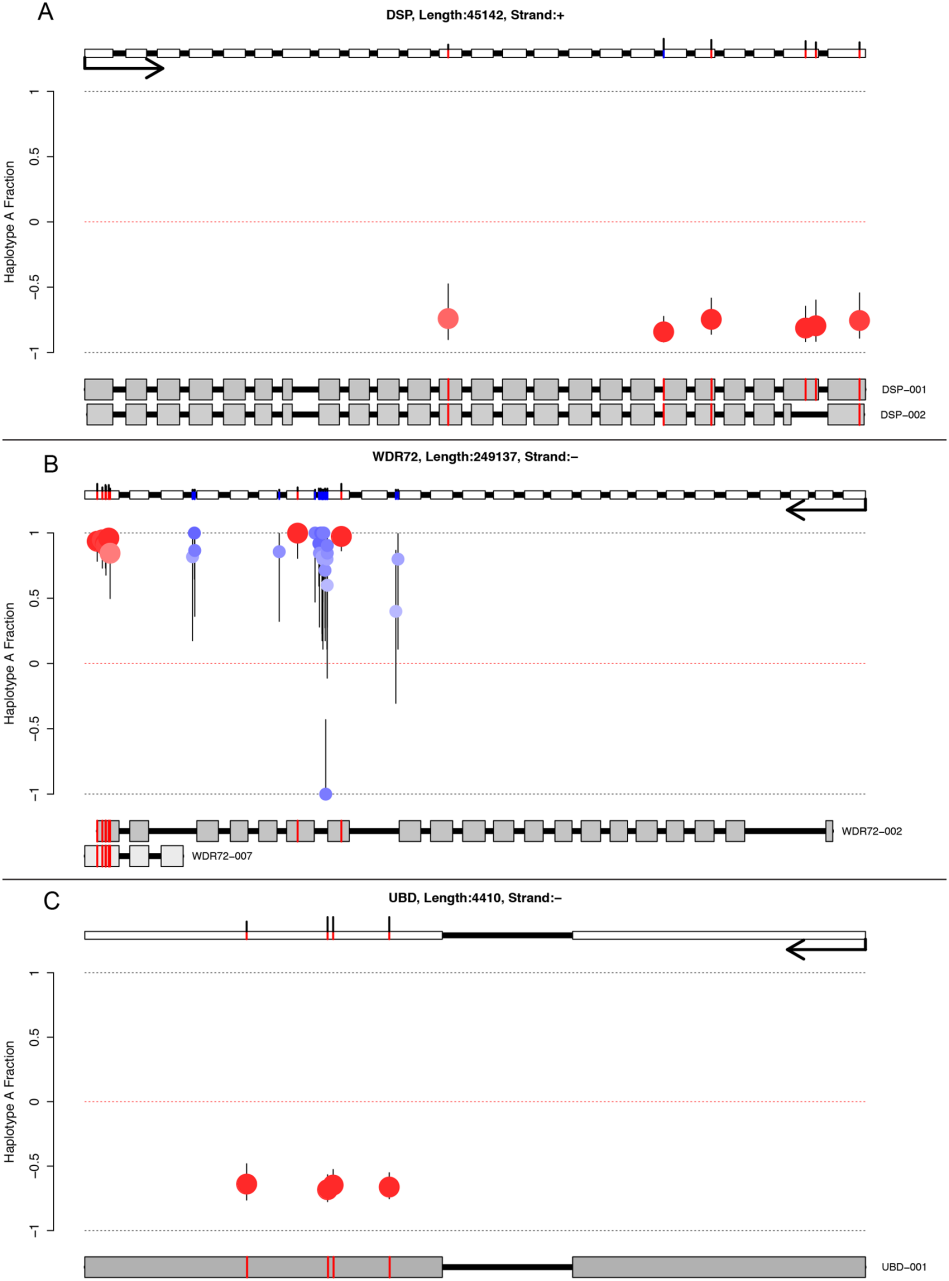
Schematic representation of three novel ASE genes in liver tissue. Red circles are exonic SNPs, blue circles intronic SNPs. SNP colour is proportional to probability of ASE (darker is more significant). Error bars represent the 95% binomial confidence interval (Pearson-Klopper). The gene model at the top shows the exonic (red) and intronic (blue) SNPs, the black sticks above these represent the read depth (minimum height represents 10 reads, maximum height 200 or more reads). Isoform models below are ordered by expression (top highest), and coloured by expression (darker is higher expressed). Only exonic SNPs are shown on transcripts, and only transcripts with at least one testable exonic SNP are drawn. Exon lengths are drawn at log2, and intron lengths at log10. Desmoplakin (*DSP*) is shown in panel (A), WD Repeat Domain 72 (*WDR72*) in panel (B) and Ubiquitin D (*UBD*) in panel (C). All three genes contain multiple imbalanced SNPs concordant with haplotype phase, indicating strong ASE. Multiple intronic SNPs were testable in *WDR72* and also show strong imbalance, suggesting that this gene was likely transcribed from a single allele. As no intronic SNPs were testable in either *DSP* or *UBD*, we cannot determine in these cases whether these genes were subject to allele-specific transcription or allele-specific post-transcriptional regulation.

Investigation of the remaining highly imbalanced genes in liver identified several other candidates showing near monoallelic expression (Fig 6). These included Desmoplakin (*DSP*), implicated in skin fragility, wooly hair syndrome [41] and heart disease [42], WD repeat domain 72 (*WDR72*) an uncharacterized tissue-restricted beta-propeller protein implicated in the tooth enamel disorder hypomaturation amelogenesis imperfecta [43], and Ubiquitin D (*UBD*) a broadly expressed ubiquitin-like protein necessary for protein degradation in which a single polymorphism linked to celiac disease [44]. Of these three genes, none have any described eQTLs in liver tissue in the eQTL Browser (http://eqtl.uchicago.edu/cgi-bin/gbrowse/eqtl/) databases (Table 2). Given the strength of ASE observed for these genes, any functional consequence in liver tissue deserves further investigation.

### Discordant direction of ASE SNP imbalance explained by splicing patterns

While there are several examples of genes with SNPs showing strong concordant direction of imbalance in these data, multiple examples exist of genes containing strong ASE SNPs with discordant direction of imbalance (Fig 5). Given recent evidence showing that much ASE in lymphoblastoid cells is isoform derived [16], and the very high confidence we have that our observed ASE SNPs are real and not sequencing artifacts, we hypothesized that the discordant SNP allelic imbalance we observed in our data derived from allele-specific isoform regulation.

**Fig 7:**
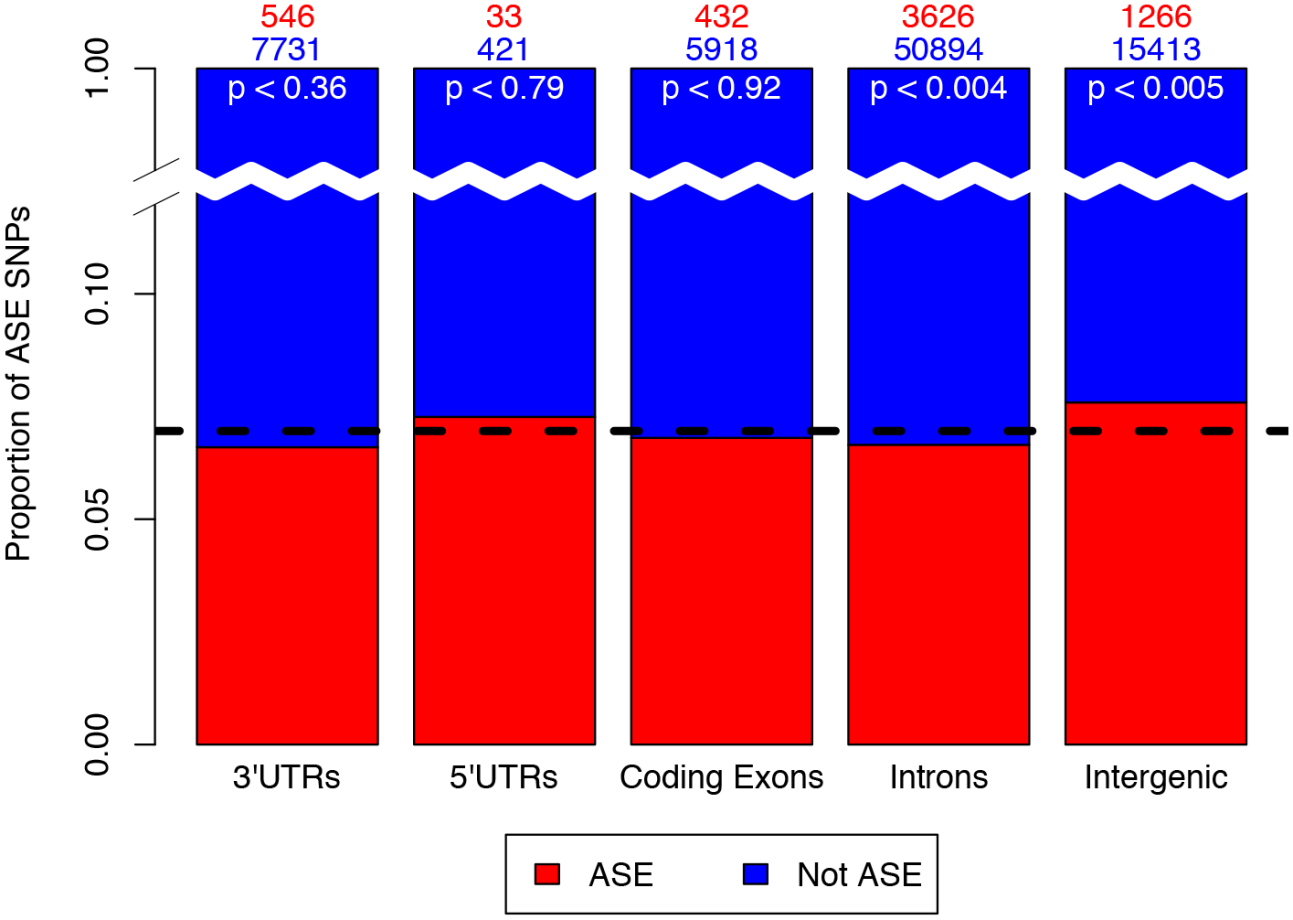
Proportion of SNPs annotated within transcript features categorised as ASE. Dashed black line represents the average proportion of all ASE SNPs. SNPs falling within intergenic regions (p < 0.005) are more likely to be classified ASE than SNPs falling in other regions. This observation is most likely due to the high amount of imprinted SNPs in the brain sample that do not have genic annotations.

In order to explore this, we began by quantifying the proportion of ASE SNPs predicted in different transcript features. We labeled every testable SNP as one or more of the following; coding exonic, intronic, 3’UTR, 5’UTR and intergenic. Using these classifications, 72,321 out of 96,658 (74.82%) testable SNPs were annotated with a single transcript feature type and retained for further analysis (SNPs overlapping multiple features were discarded). Calculating the proportion of ASE SNPs for each transcript feature, we found a higher proportion of SNPs classified as ASE in intergenic regions (8.21%, p-value < 0.005) than the average proportion of ASE SNPs residing in a single transcript feature type only (7.43%, Fig 7). ASE SNPs expressed in introns were significantly under-represented amongst all ASE SNPs (6.65%, p-value < 0.004). SNPs expressed in coding regions and 3’UTRs showed slightly less ASE SNPs than the average proportion but were not significantly different. Removing SNPs with less than perfect imputation R^2^ values did not substantially change these proportions (S16 Fig). The higher proportion of ASE SNPs in intergenic regions is most likely due to multiple SNPs located in the brain imprinted region spanning *SNURF*-*SNRPN* and *UBE3A* at chromosome 15q11-15q13, many of which have no genic annotation. The lower proportion of intronic ASE SNPs is most likely due to the lower expression of these SNPs and hence their lower power for ASE detection.

**Fig 8:**
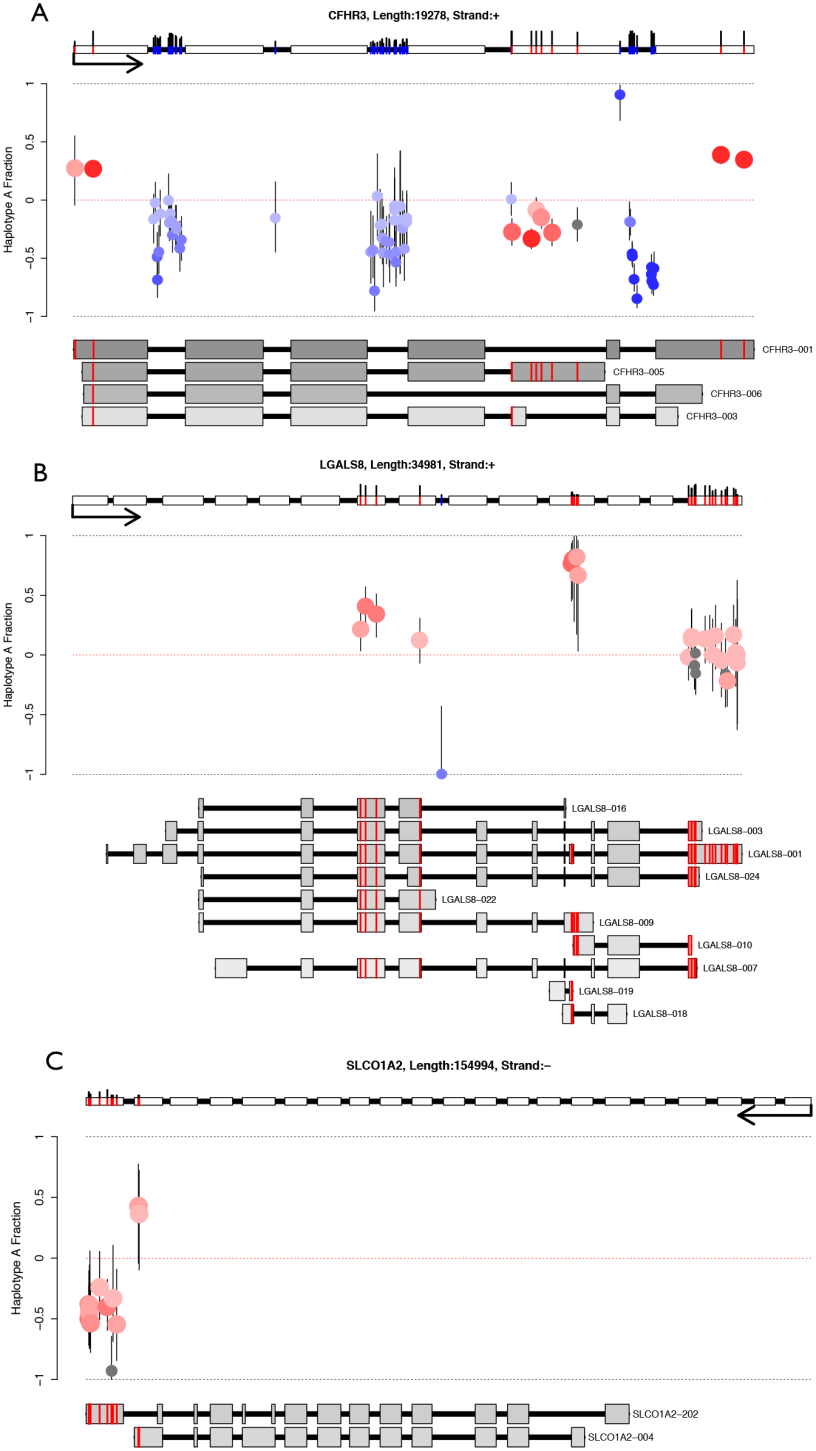
Putative allele-specific isoform expression. Red circles are exonic SNPs, blue circles intronic SNPs, gray circles are filtered SNPs (either poor alignability or SNV proximity). SNP colour is proportional to probability of ASE (darker is more significant). Error bars represent the 95% binomial confidence interval (Pearson-Klopper). The gene model at the top shows the exonic (red) and intronic (blue) SNPs, the black sticks above these represent the read depth (min height represents 10 reads, max height 200 or more reads). Isoform models below are ordered by expression (top highest), and coloured by expression (darker is higher expressed). Only exonic SNPs are shown on transcripts, and only transcripts with at least one testable exonic SNP are drawn. Exon lengths are drawn at log2, and intron lengths at log10. (A) At the *CFHR3* locus in the liver sample, two SNPs flanking either end of the gene show high allele A expression, three of which are specific to isoform *CFHR3-001*. Five SNPs show lower allele A expression in the fifth exon, and are specific to isoform *CFHR3-005*. The intronic SNPs show skewed expression towards allele B, and suggest allele-specific transcription. Taken together, these data suggest that both isoforms *CFHR3-001* and *CFHR3-003* are ASE, even though the gene as a whole does not show evidence of ASE. (B) In the liver sample, the ubiquitously expressed gene *LGALS8* shows isoform specific ASE from transcript *LGALS8*-009 (towards allele A), whereas isoform *LGALS8*-001 shows balanced expression. (C) *SLCO1A2* is a brain specific anion-transporting polypeptide, and in our brain sample it expresses two isoforms testable for ASE. Both isoforms overlap specific SNPs that show imbalanced allelic expression in opposite directions in their respective 3’UTRs. Isoform *SLCO1A2*-202 appears imbalanced towards haplotype B, and isoform *SLCO1A2*-004 towards haplotype A.

Having quantified ASE rates for different transcript features but not identifying strong enrichment, we inspected all genes with ASE SNPs individually for putative isoform ASE. Several examples of genes where SNP allelic imbalance varied considerably across the length of the gene were observed, and may be explainable by splicing patterns (Fig 8). Complement factor H-related 3 (*CFHR3*) is a liver-specific secreted protein believed to function as an inhibitor of C3 convertase activity [45]. The *CFHR3* locus in liver showed slight allelic imbalance for two highly expressed isoforms; *CFHR3-*001 towards haplotype A, and *CFHR3-*003 towards haplotype B, and an intronic SNP ASE pattern suggestive of allele-specific transcription (Fig 8a). *LGALS8* (lectin galactoside-binding soluble 8) is ubiquitously expressed protein-coding gene implicated in essential cellular functions. In the liver sample, multiple isoforms from this locus were expressed, of which *LGALS8-*009 showed imbalance expression towards haplotype A (Fig 8b), the pattern of which suggests allele-specific splicing. Finally, in the brain sample, alternative splicing resulted in two isoforms expressed from the *SLCO1A2* locus, suggesting allele-specific alternate 3’UTR expression (Fig 8c). This gene expresses an organic anion-transporting polypeptide almost exclusively in the brain, a tissue where variable use of alternative 3’UTRs has been shown to control RNA subcellular localisation [46].

In addition to putative isoform ASE, we also observed intragenic and antisense ASE. In the liver sample, expression of the small, uncharacterized protein-coding gene *RP11-422N16*.*3* initiated antisense to the third exon of Syntabulin and continued downstream for over 50kb. A large portion of this expression was detected as ASE, with 36 SNPs showing concordant direction of expression imbalance across a 14kb window (S17 Fig). In a second example, we observed antisense allele-specific expression that initiated downstream of the dihydroorotate dehydrogenase (*DHODH*) gene and proceeded for approximately 32kb (S18 Fig). These examples and the ones discussed previously indicate that interrogation of observed ASE SNPs requires additional integrative isoform expression analysis, and can potentially explain much intragenic SNP allelic imbalanced expression.

## Conclusions

In this study we performed a methodological review of bioinformatic processes for ASE quantification, made recommendations on best-practice approaches, and used these to survey SNP, gene and isoform ASE in two human tissue samples. Utilising genotyped, imputed and *de novo*-called SNPs from whole genome data maximised heterozygous locations and subsequent power to detect ASE across genes and isoforms, which we linked by computationally phased haplotypes. We confirm the diploid alignment method originally proposed in reference [28] as superior to universal alignment when quantifying ASE at the SNP level, as it increases sensitivity and specificity, and more accurately quantifies allelic expression. This method is preferable over simulation studies as it increases the data yield, testable SNPs and hence ASE discovery potential. SNPs with proximal SNVs were shown to be more likely to be detected as ASE, confirming recent analyses [32], and were therefore excluded from our analysis. Such SNPs were shown to be largely exclusive of SNPs with variable reference fraction differences between the diploid and universal alignment methods, indicating that both personalized alignment and SNP quality control are necessary for robust ASE predictions with high specificity. We estimate the SNP ASE rate in these samples to be between 2–3%, and whole gene ASE to be quite rare (restricted to a handful of genes, mostly imprinted), with the majority of observed ASE resulting from complex transcriptional events, including allele-specific transcription, splicing, antisense expression and 3’ UTR usage. The identification of multiple novel ASE genes and isoforms here should prove informative for future studies exploring the functional consequences of genetically linked expression variability.

## Materials and Methods

### Ethics Statement

RNA and DNA samples for this study were purchased commercially and no ethical approval was required.

### Sample Acquisition

Human RNA and DNA from two different individuals were sourced from BioChain, pre- extracted from the following human tissues: liver (Cat. #S1234149, donor ID NB0030, male, 30 years old) and brain (Cat. #S1234096, donor ID H13126N, male, 26 years old.).

### Whole Genome Sequencing and Analysis

Whole genome libraries were constructed using the Illumina TruSeq DNA PCR-Free LT Sample Prep Kit (Set A, Illumina, Cat. #FC-121-3001). 350bp insert libraries were prepared from both samples using 1μg of gDNA according to the manufacturers protocol (Low Sample Protocol, Illumina, Part #: 15036187). The final libraries were quality checked using the BioAnalyzer High Sensitivity DNA Kit (Agilent, Cat. #5067–4626), and were quantified by qPCR (KAPA BioSystems, Cat. #KK4824). The libraries were pooled in equimolar ratios, and sequenced twice as 100bp paired-end Illumina HiSeq 2500 Rapid runs. R2 reads from the first sequencing run were excluded due to low quality values. All remaining sequence data was merged for alignment and analysis.

Whole genome sequence (WGS) data was aligned using BWA v0.6.2-r126-tpx [47] with default parameters for both the *aln*, *sampe* (for the mate-pair data) and *samse* (for the fragment data) commands. Alignment files were transformed into BAM files and indexed using SAMTools v0.1.17 [48]. SNPs were called using qSNP v1.0 [49] with the following approach: WGS aligned reads were filtered for PCR duplicates, and only those with an alignment length greater than 34bp (CIGAR M> = 34), an MD mismatch tag < = 4, and an SM tag value > 10 included for SNP calling. A SNP was called as heterozygous using heuristic rules as follows: if the alternate allele count was greater than three reads when the total read depth was 12 or less, or greater than four reads when the total read depth was between 13 and 30, or at least 25% of the reads when there were 31 or more reads present at that location. VCF files for *de novo* called SNPs are provided in S2 Dataset.

Indels were called with Pindel version 0.2.4s [50] using default parameters. Only small insertions and deletions (< 200bp) were kept for analysis. Any putative indel contiguous with or embedded within a homopolymer of seven or more bases, or overlapping a known simple repeat or satellite region defined by RepeatMasker (A.F.A. Smit, R. Hubley & P. Green; http://repeatmasker.org) was removed from the analysis. Indels were called as heterozygous if the number of supporting reads (those containing the insertion or deletion) divided by the number of informative reads (high quality alignments) was between 0.2 and 0.7. Indel calls are provided in S2 Dataset.

### Microarray-based SNP genotyping

Samples were genotyped using the HumanOmni1M-QuadBeadChip. Briefly, 200ng of genomic DNA per sample was whole-genome amplified and hybridized to the chip as per manufacturer’s instructions (Illumina, San Diego CA). SNP arrays were scanned on iScan and data was processed using the Genotyping module (v1.8.4) in GenomeStudio (v2010.3). Where possible, SNP coordinates were converted from human genome v18 to human genome v19 using the UCSC LiftOver tool (http://genome.ucsc.edu/cgi-bin/hgLiftOver). Any SNPs with ambiguous sex chromosome positions (chrXY), or with a GC score less than 0.7, were excluded from analysis.

### SNP imputation and haplotype phasing

All SNPs with matching genotypes from both the WGS and SNP array were categorized as high confidence and used for haplotype phasing. High confidence SNPs were filtered to include only those with a minor allele frequency greater than or equal to 0.05. SNP imputation and haplotype phasing is most effective when using more related individuals, such as those of the same ethnicity. Phased haplotype data for 629 individuals were downloaded on April 12, 2012 from the MaCH website (http://www.sph.umich.edu/csg/abecasis/MACH/download/1000G-2010-08.html). In order to establish the ethnicity of the two samples, all chromosome 22 high confidence SNPs common with the 1000 genome data were extracted and encoded as either heterozygous or homozygous, then used to generate a principal component analysis plot using the mixOmics [51] R package invoking commands ‘pca.tune’ then ‘pca’ with parameters ‘ncomp = 3, center = TRUE, scale. = TRUE’. Both samples were determined to be Asian based on the proximity of these samples to clear clusters of individuals of known Asian ethnicity (S19 Fig). SNPs were then imputed and haplotypes phased based on the appropriate ethnicity subset of 1000 genome data using the software MaCH (v1.0.18.c) [52], with parameters ‘–quality—compact—autoFlip—mle—mldetails—greedy—phase—states 500—rounds 50’. All imputed SNPs were cross-checked against WGS data, and only those with matching genotypes and passing the heuristic tests described in the Whole Genome Sequencing and Analysis section retained for ASE testing.

### RNA library construction and sequencing

Ribosomal depletion and RNA library construction was performed using Truseq Stranded Total RNA kit with Ribo-Zero Human/Mouse/Rat Set A (Illumina, Cat. #RS-122-2201). Two technical replicates were prepared from both liver and brain RNA samples with 1μg of total RNA used as starting material. The library preparations were performed according to the manufacturer's protocol (Truseq Stranded Total RNA Sample Prep Guide, Illumina Part # 15031048) with minor modifications to the fragmentation and the number of PCR cycles. After the addition of Elute, Prime, Fragment High Mix to the ribosomal RNA-depleted RNA, samples were incubated on ice and followed immediately by first strand synthesis. The number of PCR cycles used to amplify the final libraries was decreased from 15 to 8 in order to increase library complexity. The final cleaned libraries were quantified using the Bioanalyzer High Sensitivity DNA Kit (Agilent, Cat. #5067–4626).

All the RNA libraries were indexed allowing sequencing of multiple samples on one slide. All four libraries were pooled together in an equimolar ratio and sequenced as 100 bp paired-end Illumina HiSeq 2500 Rapid run.

### RNA-seq alignment and personalised BAM construction

Personalised haplotype reference genomes were constructed from phased and WGS verified heterozygous and homozygous alternate SNPs using the vcf2diploid.pl tool [28]. As we did not know the specific parent of origin for our samples, but we did have phased haplotype information, haplotype A was arbitrarily assigned maternal, and haplotype B paternal. RNA-seq data was aligned to each personalised haplotype reference and the universal reference with the package tophat2 v2.0.6 [53], using the Ensembl v71 gene model, with the following parameters: ‘—b2-very-sensitive—read-realign-edit-dist 0 —b2-N 1—library-type fr-unstranded’.

Alignments were merged using custom written software (available on request). This software selects the most optimal alignments from all three alignment sets (maternal, paternal and universal). Alignments were compared in pairs, with the pair of alignments with the smallest sum of edit distances (SAM tag NM values) chosen and retained in the final BAM file. In the case of a tie, the universal alignments were selected. An alignment that produced a successfully mapped read pair was selected over one producing one or more unmapped reads. In the case of multi-mapped reads, the alignment with the smallest edit distance was used. No discrimination on the number of multi-mapped reads was made regarding selection of read alignment sets. All alignments were tagged with their origin (maternal, paternal or universal) using the custom SAM tag ZR:Z:<origin>, allowing for identification of the read origin once they are written to the BAM file.

Following BAM construction, alignments were filtered for PCR duplicates using the picard v1.88 MarkDuplicates utility (http://picard.sourceforge.net/). Alignments were further filtered to remove multi-mapped reads, reads with low mapping quality (MAPQ < 9) and all reads aligning to the mitochondrial chromosome (to expedite later analysis). All subsequent analyses were performed on these modified BAM files.

### Expression calculations

Gene expression was quantified using custom scripts by summing the total number of R1 fragments with start positions between annotated Ensembl v71 genic boundaries, then calculating the reads per kilobase per million mapped reads (RPKM, [22]). Isoform expression was quantified using cufflinks v2.0.2 [54] on the same BAM files with parameters ‘-G <file>-v—no-update-check-M <file>-u-b <file>—library-type fr-unstranded—max-bundle-frags 1000000 <file>‘, where the mask file contained all Ensembl v71 rRNA genes, the reference was hg19, the GTF file was Ensembl v71.

### Quantification of SNP ASE

Allelic expression from RNA-seq reads at all heterozygous SNPs was counted using the mpileup command in Samtools v0.1.17 [48] with parameters ‘-A B Q 0 M 100-d 1000000’. Only SNPs with at least 10 unique reads were considered for further analysis.

For testable SNPs the fraction of reads containing the reference allele was calculated. All SNPs were then binned by ordered genotype (i.e. the specific reference—variant combination), and the median of all observed SNP reference fraction values for each genotype bin calculated for each library, as described recently [16]. In addition to calculating reference fraction values, the probability of allelic imbalance for each SNP was calculated using a simple two-sided binomial test, with the expected probability set to the appropriate observed median reference fraction for that SNPs’ ordered genotype. Any testable SNP with reference fraction outside the median reference fraction +- 0.15 and a probability of imbalance < = 0.05 (two-sided binomial test), was classified as ASE.

### Quantification of Genic ASE

All testable SNPs passing alignment quality control were grouped by genic boundaries defined by Ensembl v71 annotations. Using the phased haplotype information obtained during SNP imputation (see above), allelic counts for each SNP for both haplotypes were summed independently. The probability of the observed allelic ratio was calculated using a two-sided binomial test and used to rank genes from most imbalanced to least imbalanced.

## Supporting Information

S1 Fig Proportion heterozygosity for imputation reference data set and samples in this study. Brain and Liver samples have lower proportion heterozygosity than the mean of the reference data set. These proportions were consistent (slightly lower than the mean) with all samples from the 1000G reference data set used for imputation (downloaded on April 12, 2012 from the MaCH website http://www.sph.umich.edu/csg/abecasis/MACH/download/1000G-2010-08.html).
(PDF)

S2 Fig Gene expression correlations between replicates and tissues. (A) Correlations between RPKM gene expression values for brain sample replicate one against replicate two, (B) liver replicate one against replicate two, and between replicates for difference tissues; (C) replicate one brain against liver and (D) replicate two brain against liver. Both libraries from the same tissues are sourced from the same starting RNA.
(PDF)

S3 Fig Comparison of allelic expression reference fraction for diploid and universal alignment methods. (A) Density plot shows improvement of reference fraction for diploid alignment (blue, fraction = 0.503) compared to universal method (red, fraction = 0.516). (B) Boxplot of reference fractions for diploid and universal alignment methods shows improvement in mean reference fraction for diploid against universal alignment methods.
(PDF)

S4 Fig Proportion of ASE SNPs binned by proximity to unphased heterozygous *de novo* SNVs. An increase in likelihood of SNPs being classified as ASE (above the average ASE classification rate, indicated by the dotted line) is observed as far away as 10kb of both SNPs (A) and indels (B). This suggests that polymorphisms not included in the phased haplotypes (nor used in the diploid genome construction) affect the alignment at a much greater distance from the testable SNP than the length of a read (100bp), possibly due to spliced reads spanning a longer genomic distance. Alternatively, this result may indicate that SNPs in areas of high polymorphism may be more likely to be ASE.
(PDF)

S5 Fig Overlap between SNPs removed for either SNV proximity or alignability. SNPs with imperfect alignability typically do not have proximal indels nor multiple proximal other SNPs. Additionally, SNPs with large changes in reference fraction (+- 0.15) also do not tend to fall into these categories. These mutually exclusive attributes of SNPs indicate that when testing for ASE multiple alignment quality control mechanisms are necessary.
(PDF)

S6 Fig Comparison of imputation R^2^ values with proportion of ASE classification, proportion heterozygosity, and proportion of WGS verification status. (A) Proportion of all testable SNPs classified as ASE binned by R^2^ values. Poorer R^2^ values have higher likelihood of being called ASE. (B) Proportion of all testable SNPs classified as ASE after removing SNPs with alignment confounders, indicating substantial reduction in ASE SNPs with poor R^2^ values. (C) Total number of SNPs failing WGS verification binned by R^2^ values. SNPs with lower R^2^ values make up the highest number of SNPs failing WGS verification, and SNPs with high R^2^ make up a large proportion also. (D) Proportion of all SNPs failing WGS verification, binned by R^2^ values. (E) Proportion of WGS verified heterozygous SNPs binned by R^2^ values. (F) Proportion of SNPs heterozygous SNPs failing WGS verification, binned by R^2^ values.
(PDF)

S7 Fig Smoothed scatter plots of imputation R^2^ values plotted against DNA and RNA reference fractions. (A) DNA reference fraction plotted against imputed R^2^ values for all WGS verified heterozygous SNPs, indicating an average reference fraction of 0.5, consistent with true heterozygosity. (B) Smoothed scatter plot of DNA reference fraction against imputed R^2^ value for all SNPs imputed as heterozygous, but failing WGS verification. (C) Smoothed scatter plot of RNA reference fraction against imputed R^2^ values for all ASE testable SNPs (WGS verified heterozygous, and RNA-seq coverage > = 10 PCR de-duplicated reads).
(PDF)

S8 Fig SNPs with perfect R^2^ values proximal to SNVs are more likely to be ASE. Figure is a recreation of Fig 2, excluding all SNPs with an R^2^ value < 1. (A) SNPs binned by genomic distance to nearest heterozygous indel. (B) SNPs binned by nearest heterozygous un-phased *de novo* called SNP. (C) SNPs binned by the number of proximal heterozygous SNPs. (D) SNPs binned by genomic alignability. SNPs with perfect R^2^ values are confounded by proximal SNVs and poor alignability.
(PDF)

S9 Fig Proportion of ASE SNPs with perfect R^2^ values binned by proximity to unphased heterozygous *de novo* SNVs. Figure is a recreation of S4 Fig, excluding all SNPs with an R^2^ value < 1. Increased proportion of ASE classified SNPs proximal to unphased SNPs and indels extends to a distance beyond the length of a read. Removing SNPs with R^2^ values < 1 slightly reduces the distance of this confounder for indels (A), and the severity of the confounder for unphased SNPs (B), but does not eliminate it completely in either situation.
(PDF)

S10 Fig Binomial 95% confidence interval as a function of the number of tests (read depth). As read depth increases the standard deviation of the binomial decreases.
(PDF)

S11 Fig Sequence read depth correlation at testable SNPs between library replicates for (A) liver and (B) brain. While the correlation between SNP allelic expression differed substantially, the correlation between SNP sequence depth was much stronger.
(PDF)

S12 Fig SNP reference fraction replicate correlations with increasing read depth thresholds. (A) Scatter plots for correlations show that as read depth increases, so too does correlation. (B) Bar plots show the proportion of all SNPs classified as either not ASE in both replicates, ASE in both replicates, or ASE in one or the other replicate. (C) Bar plots show proportion of SNPs classified as ASE in any replicate as read depth increases. Increasing the read depth dramatically improves correlation, but at a large cost of number of testable SNPs, and hence power to detect ASE.
(PDF)

S13 Fig Overlaps of ASE classified genes using different testing criteria. In all plots, genes are only tested for ASE if they contain at least two testable SNPs. (A) Very strict testing is achieved by classifying a gene as ASE if it contains at least one replicated ASE SNP and all SNPs within the gene show direction of imbalance concordant with the haplotype or (C) any direction. (B) Less stringent testing is shown for genes when they can contain SNPs of which none are classified as ASE (but may still be slightly imbalanced), but are still concordant in imbalance direction, or (D) with any direction of imbalance. The most optimal parameters to maximize specificity, sensitivity and overlap between replicates are shown in panel (C).
(PDF)

S14 Fig SNP ASE testability and intersection of SNP ASE Status. Of all SNPs testable in both replicates, less than 13.47% were testable in both tissues (top). Of these SNPs, a tiny percentage (0.39%; n = 8), were classified as ASE in both tissues (bottom).
(PDF)

S15 Fig Gene ASE schematic for (A) *ERAP2* and (B) *GSTA2* showing strong allele-specific expression for both genes. Red circles are exonic SNPs and blue circles intronic SNPs. SNP colour is proportional to probability of ASE (darker is more significant). Error bars represent the 95% binomial confidence interval (Pearson-Klopper). The gene model at the top shows the exonic (red) and intronic (blue) SNPs, the black sticks above these represent the read depth (min height represents 10 reads, max height 200 or more reads). Isoform models below are ordered by expression (top highest), and coloured by expression (darker is higher expressed). Only exonic SNPs are shown on transcripts, and only transcripts with at least one testable exonic SNP are drawn. Exon lengths are drawn at log2, and intron lengths at log10.
(PDF)

S16 Fig Proportion of SNPs with perfect R^2^ imputation values (R^2^ = 1) annotated within transcript features categorised as ASE. Dashed black line represents the average proportion of all ASE SNPs. SNPs falling within intergenic regions (p < 0.002) are more likely to be classified ASE than SNPs falling in other regions.
(PDF)

S17 Fig Extended allele-specific expression of gene *RP11-422N16*.*3* antisense to Syntabulin (*SYBU*). Integrative Genome Viewer (IGV [55]) screenshot where SNPs are represented as white columns within the top six gray tracks. Three tracks for each SNP with sufficient strand-specific expression are shown; the—log10 of binomial probability, reference fraction and haplotype A fraction. These are clustered into two groups, negative strand (upper three) and positive strand (lower three). RNA-seq data are shown below these tracks (red reads are transcribed from the positive strand, and blue from the negative). Gene models below the RNA-seq show gene start, end, and exon structure. A large block of 36 SNPs is observed on the positive strand showing concordant direction of allelic expression imbalance, initiating antisense to *SYBU* at the *RP11-422N16*.*3* locus and running downstream for approximately 50kb. This extended un-annotated transcription, observed in the liver sample, is likely to show allelic expression imbalance along the entire transcript, however only the window of 14kb contains heterozygous SNPs and is testable.
(TIF)

S18 Fig Un-annotated allele-specific expression antisense to Dihydroorotate dehydrogenase (*DHODH*). Integrative Genome Viewer (IGV [55]) screenshot where SNPs are represented as white columns within the top six gray tracks. Three tracks for each SNP with sufficient strand-specific expression are shown; the—log10 of binomial probability, reference fraction and haplotype A fraction. These are clustered into two groups, negative strand (upper three) and positive strand (lower three). RNA-seq data are shown below these tracks (red reads are transcribed from the positive strand, and blue from the negative). Gene models below the RNA-seq show gene start, end, and exon structure. 22 SNPs show strong evidence of ASE on the negative strand (top tracks). This allele-specific expression initiates downstream of and antisense to *DHODH*, and proceeds for a distance of approximately 32kb. This un-annotated antisense transcription also displays evidence of splicing indicating post-transcriptional regulation.
(TIF)

S19 Fig PCA plot of samples from this study and the 1000 Genome Project pilot data. The two samples used in this study cluster with the Asian samples, indicating they are of Asian descent.
(PDF)

S1 Table Number of genotyped, imputed, and quality control passing heterozygous SNPs for each sample. (XLSX)

S2 Table Number of PCR de-duplicated, uniquely aligning R1 reads per library and alignment method. (XLSX)

S3 Table Read mapping differences at expressed heterozygous SNPs. (XLSX)

S4 Table SNP ASE classification differences between diploid and universal alignment methods. (XLSX)

S5 Table Number of SNPs removed from the analyses due to proximity to SNVs. (XLSX)

S6 Table Number of SNPs classified as ASE in each replicate, and the overlaps between replicate classifications as minimum read depth increases. (XLSX)

S1 Dataset Data for all testable SNPs and genes, with allele read counts, depth, reference fractions and imbalance probabilities. (GZ)

S2 Dataset VCF files for un-phased SNPs and indels. (GZ)
