# Supplementary figures and images for: Recommendations for Accurate Resolution of Gene and Isoform Allele-Specific Expression in RNA-Seq Data
Source: PLoS One. 2015 May 12;10(5):e0126911. doi: 10.1371/journal.pone.0126911 (PMC4428808; doi:10.1371/journal.pone.0126911)

# Proportion Heterozygous SNPs

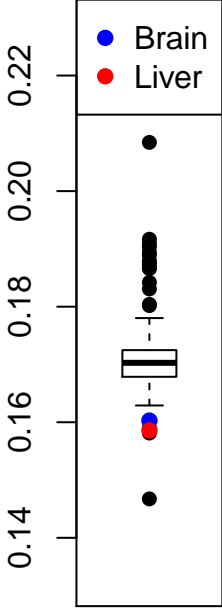

Supplement: S1 Fig — Brain and Liver samples have lower proportion heterozygosity than the mean of the reference data set. These proportions were consistent (slightly lower than the mean) with all samples from the 1000G reference data set used for imputation (downloaded on April 12, 2012 from the MaCH website http://www.sph.umich.edu/csg/abecasis/MACH/download/1000G-2010-08.html). (PDF) [file pone.0126911.s001.pdf]

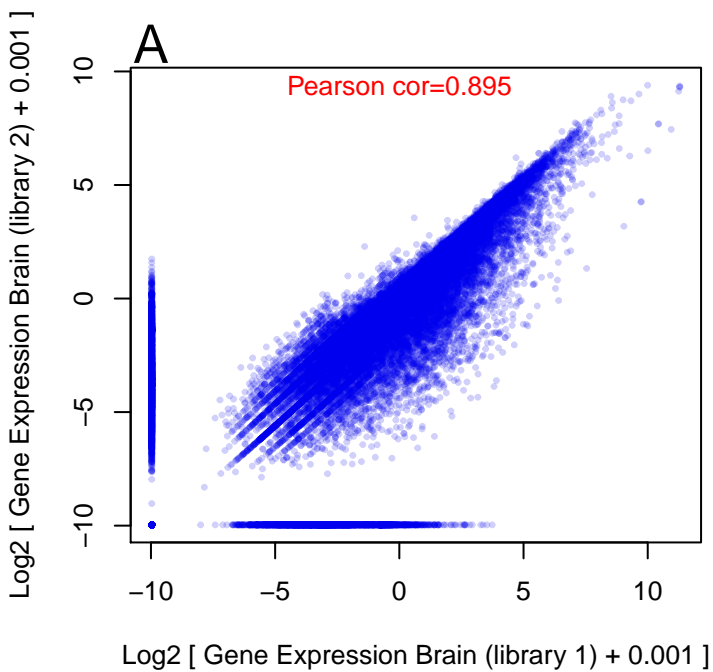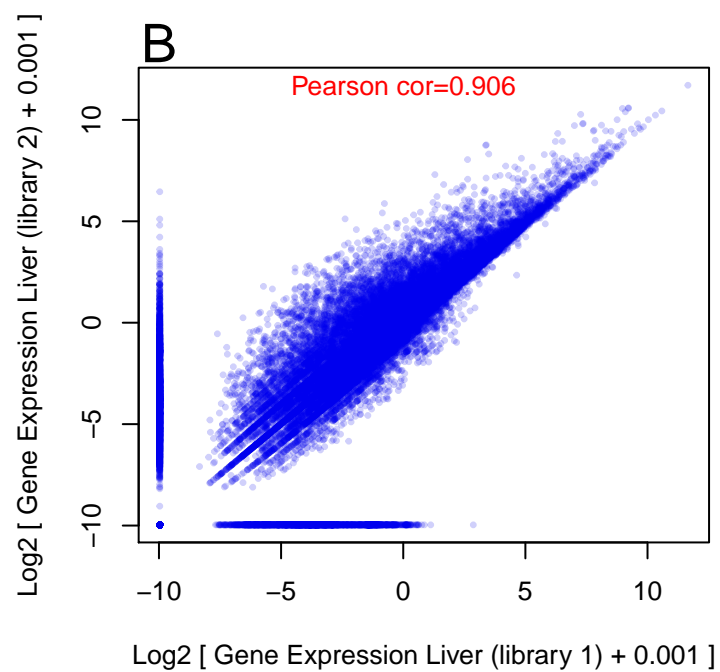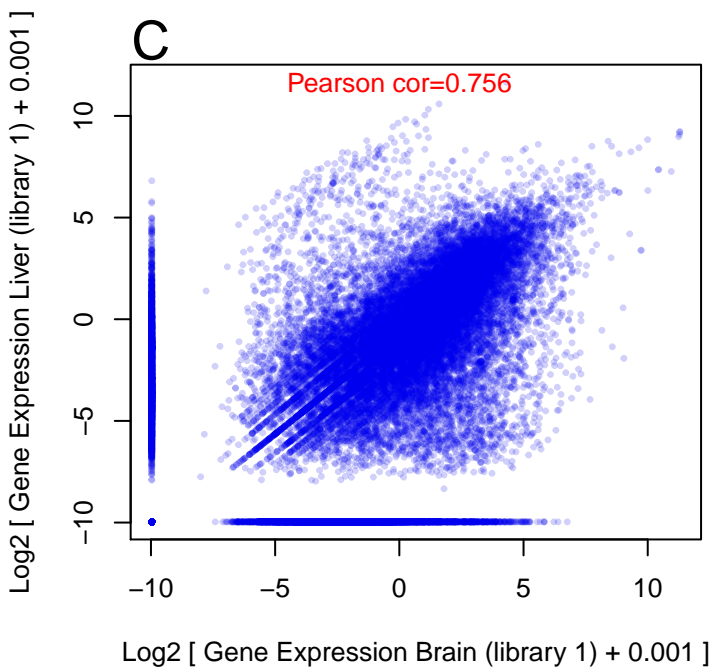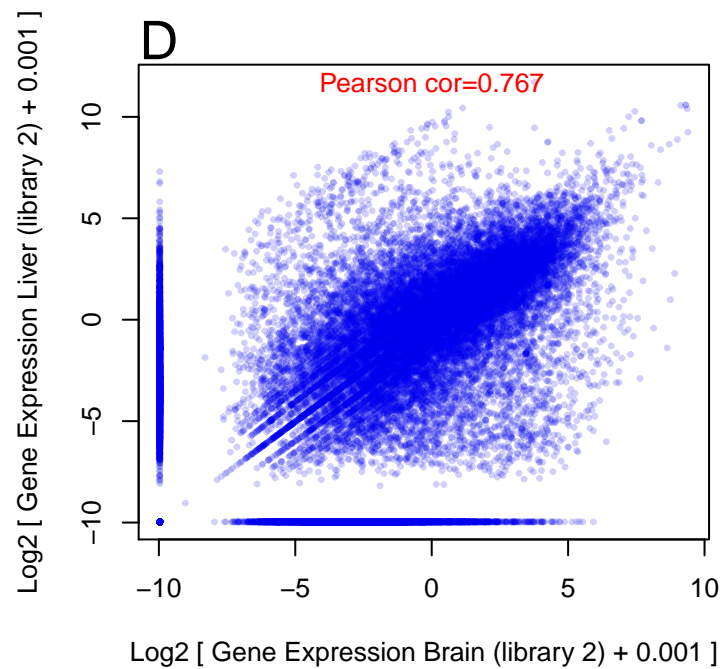

Supplement: S2 Fig — (A) Correlations between RPKM gene expression values for brain sample replicate one against replicate two, (B) liver replicate one against replicate two, and between replicates for difference tissues; (C) replicate one brain against liver and (D) replicate two brain against liver. Both libraries from the same tissues are sourced from the same starting RNA. (PDF) [file pone.0126911.s002.pdf]

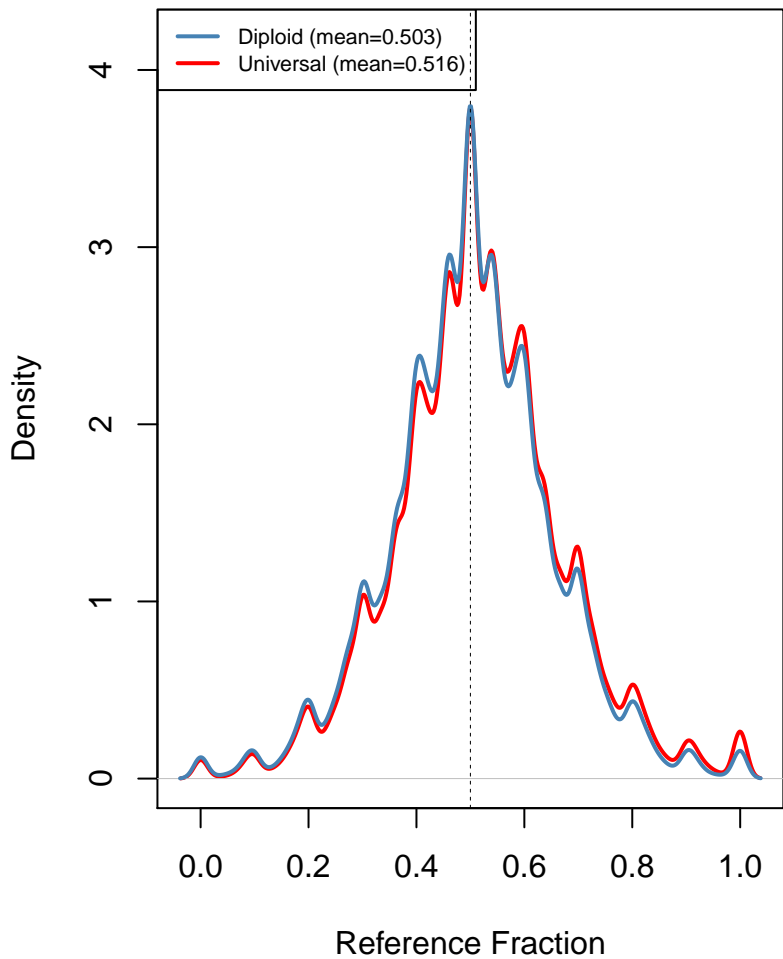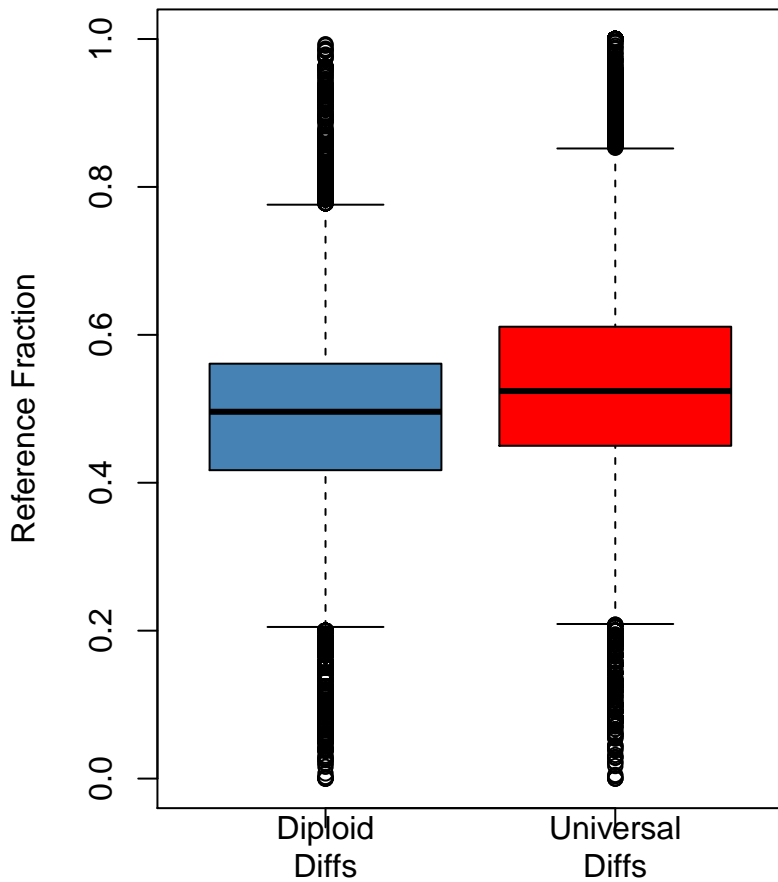

Supplement: S3 Fig — (A) Density plot shows improvement of reference fraction for diploid alignment (blue, fraction = 0.503) compared to universal method (red, fraction = 0.516). (B) Boxplot of reference fractions for diploid and universal alignment methods shows improvement in mean reference fraction for diploid against universal alignment methods. (PDF) [file pone.0126911.s003.pdf]

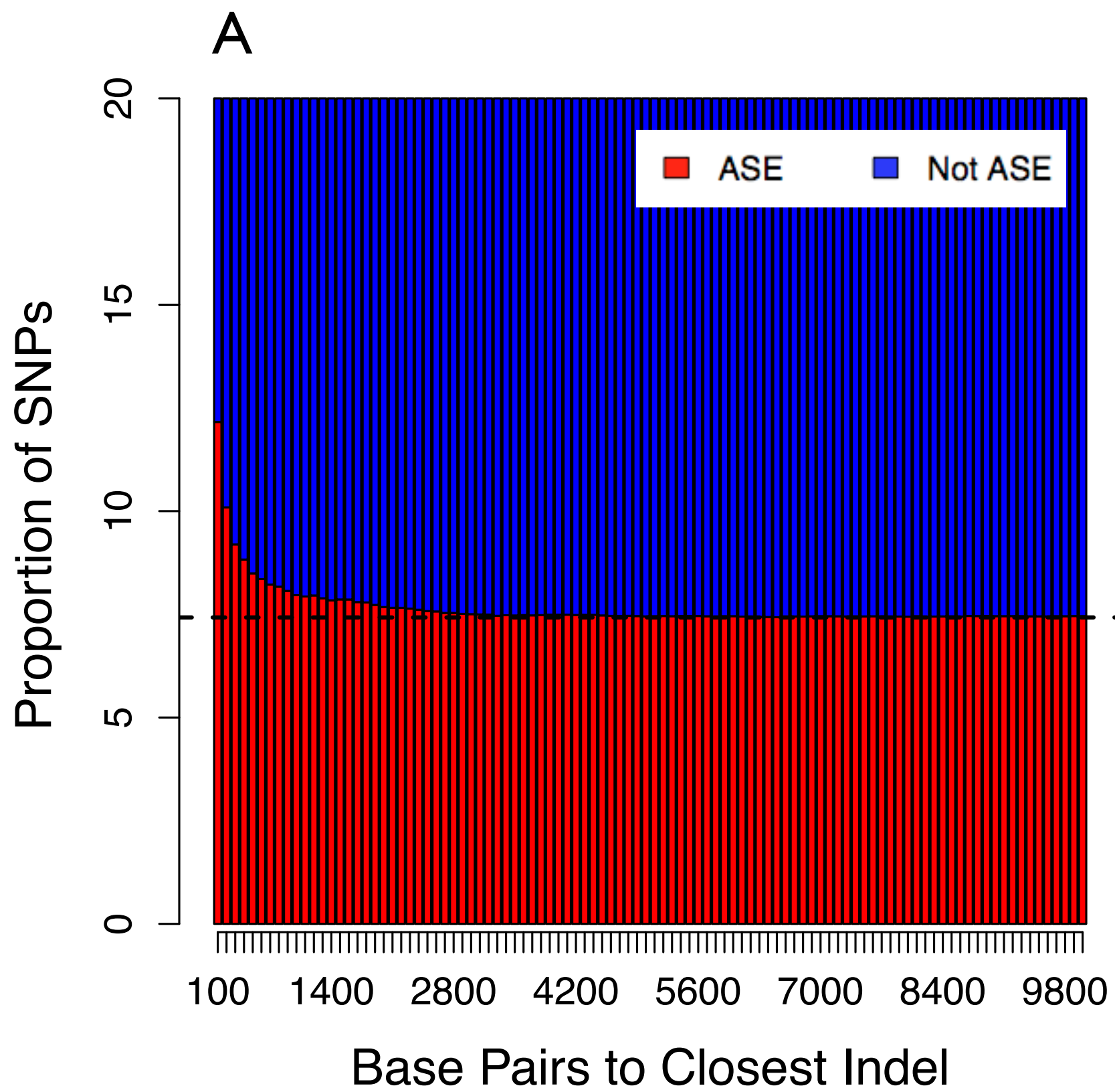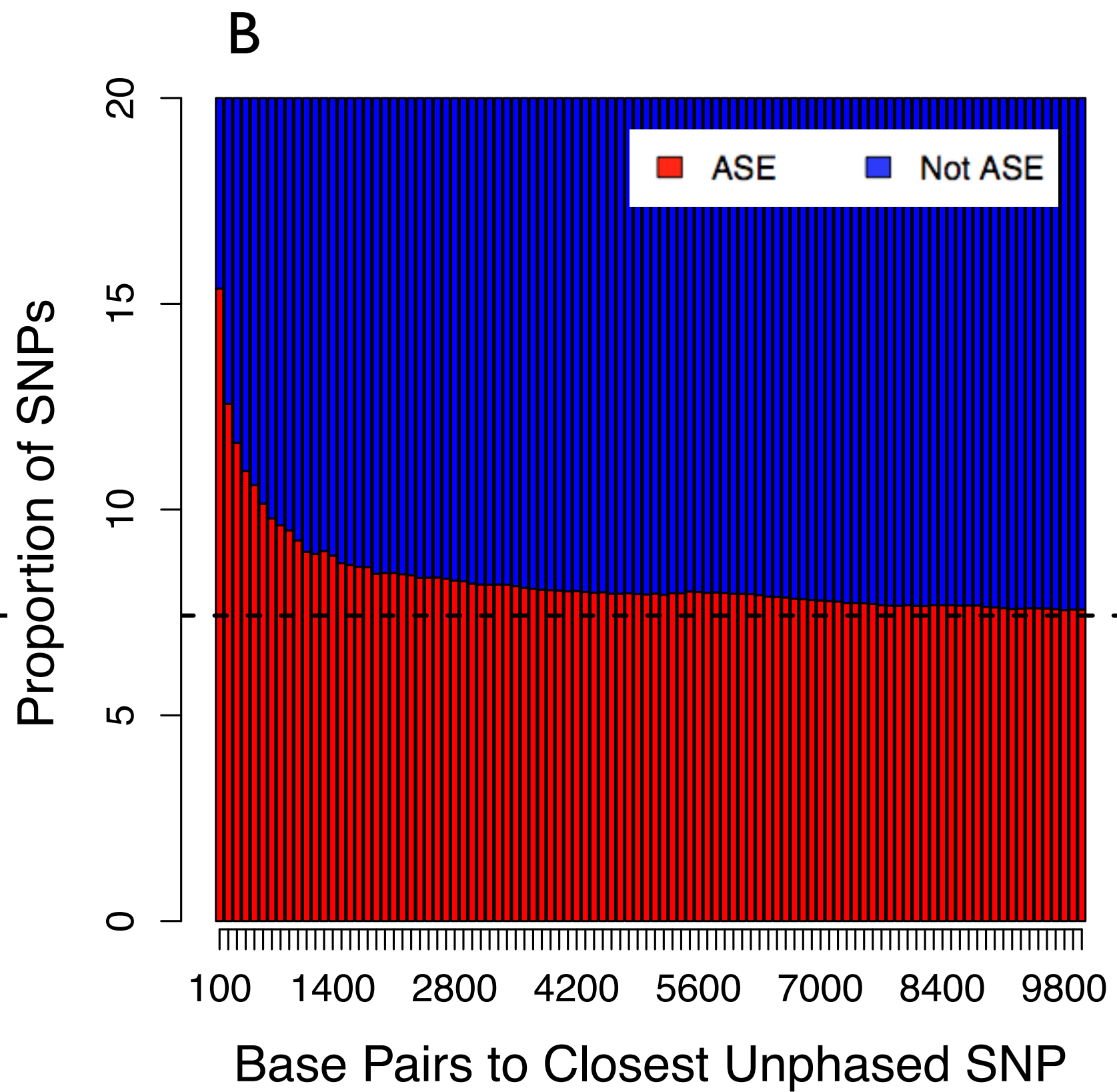

Supplement: S4 Fig — An increase in likelihood of SNPs being classified as ASE (above the average ASE classification rate, indicated by the dotted line) is observed as far away as 10kb of both SNPs (A) and indels (B). This suggests that polymorphisms not included in the phased haplotypes (nor used in the diploid genome construction) affect the alignment at a much greater distance from the testable SNP than the length of a read (100bp), possibly due to spliced reads spanning a longer genomic distance. Alternatively, this result may indicate that SNPs in areas of high polymorphism may be more likely to be ASE. (PDF) [file pone.0126911.s004.pdf]

# Brain

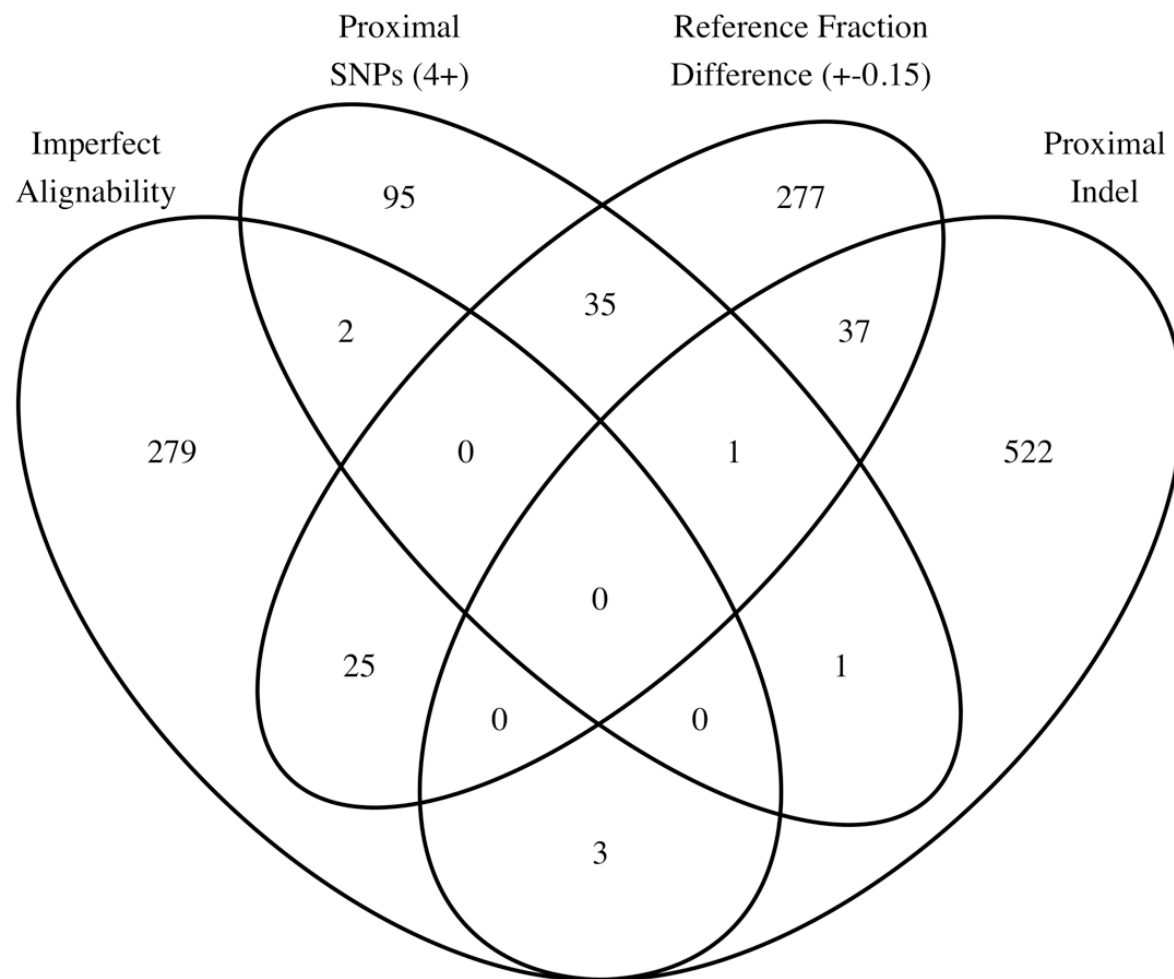

# Liver

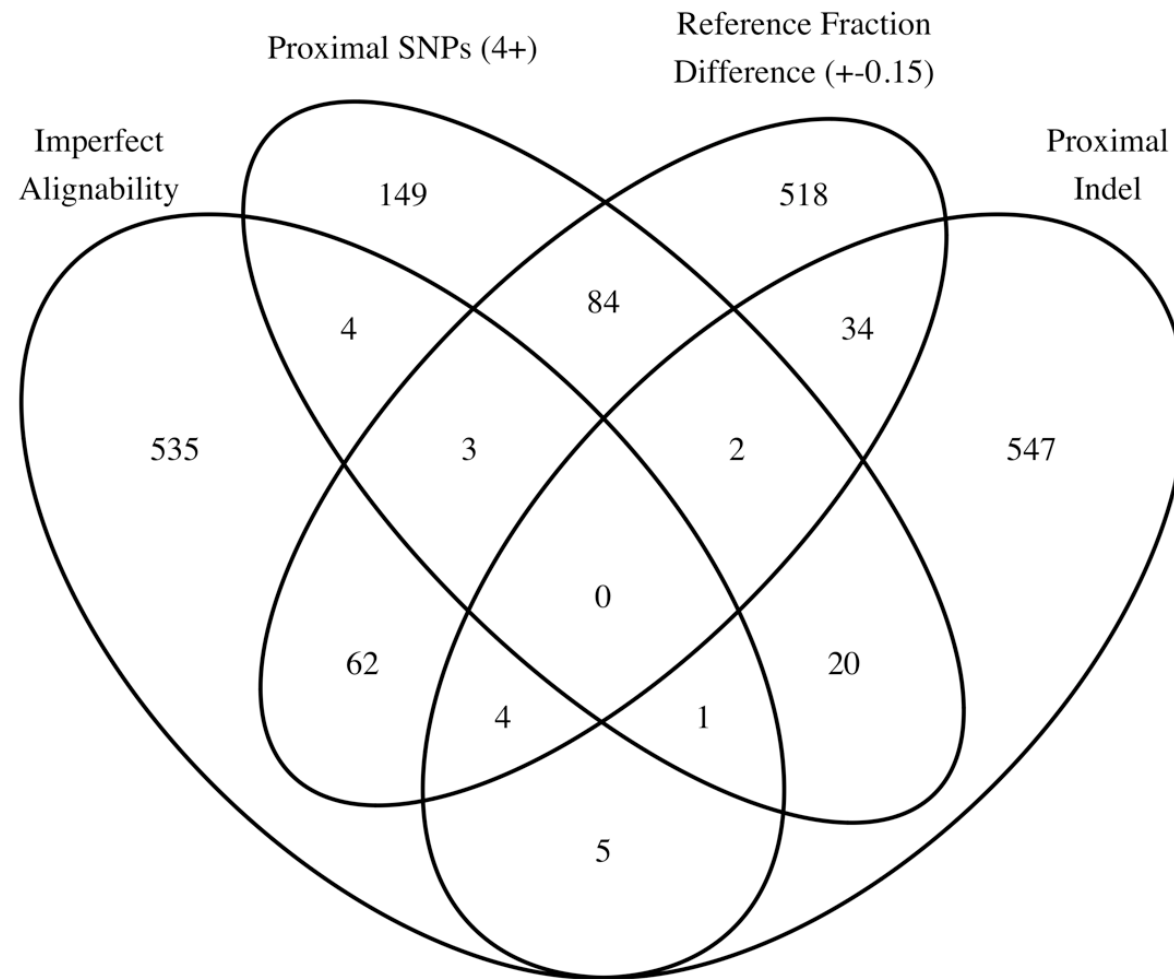

Supplement: S5 Fig — SNPs with imperfect alignability typically do not have proximal indels nor multiple proximal other SNPs. Additionally, SNPs with large changes in reference fraction (+- 0.15) also do not tend to fall into these categories. These mutually exclusive attributes of SNPs indicate that when testing for ASE multiple alignment quality control mechanisms are necessary. (PDF) [file pone.0126911.s005.pdf]

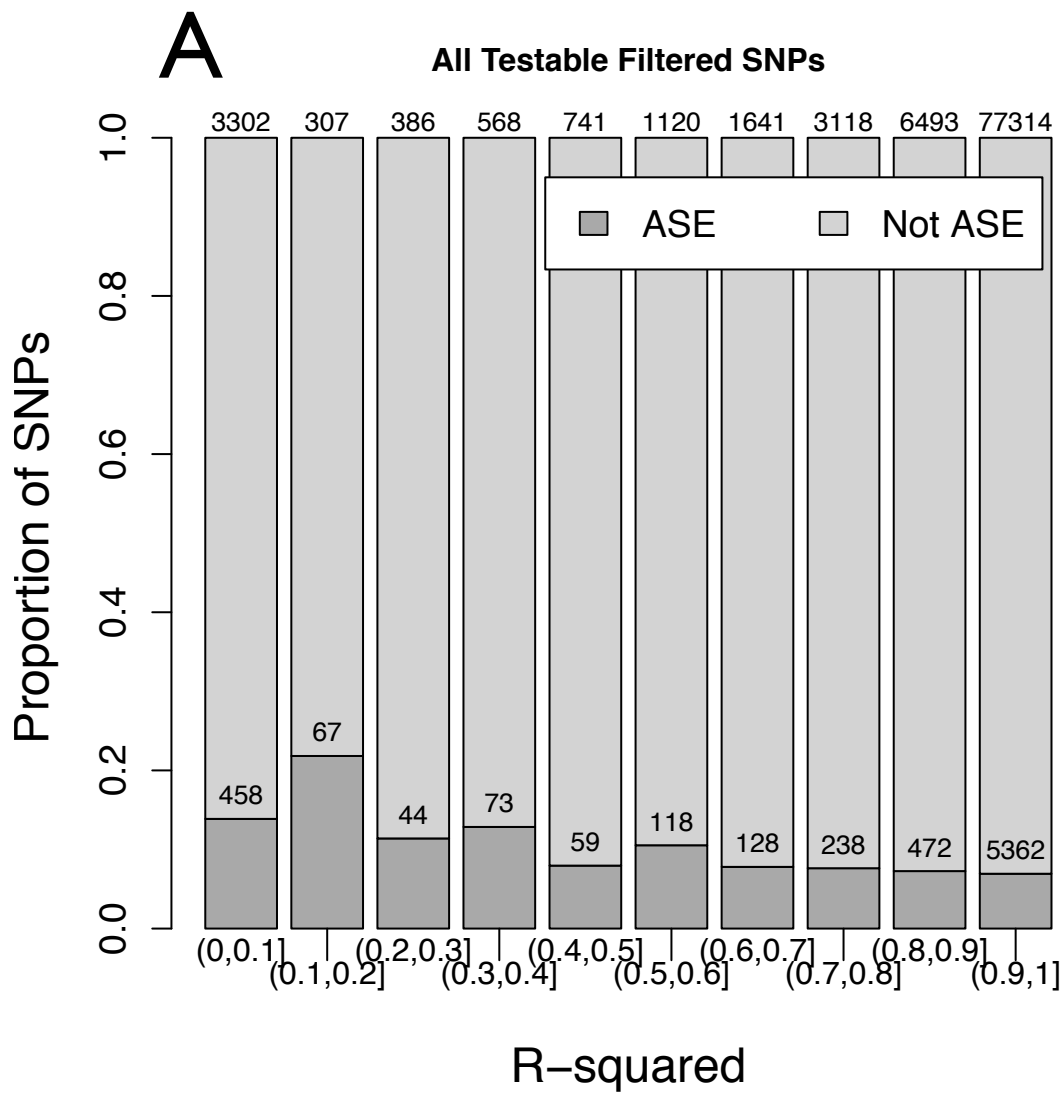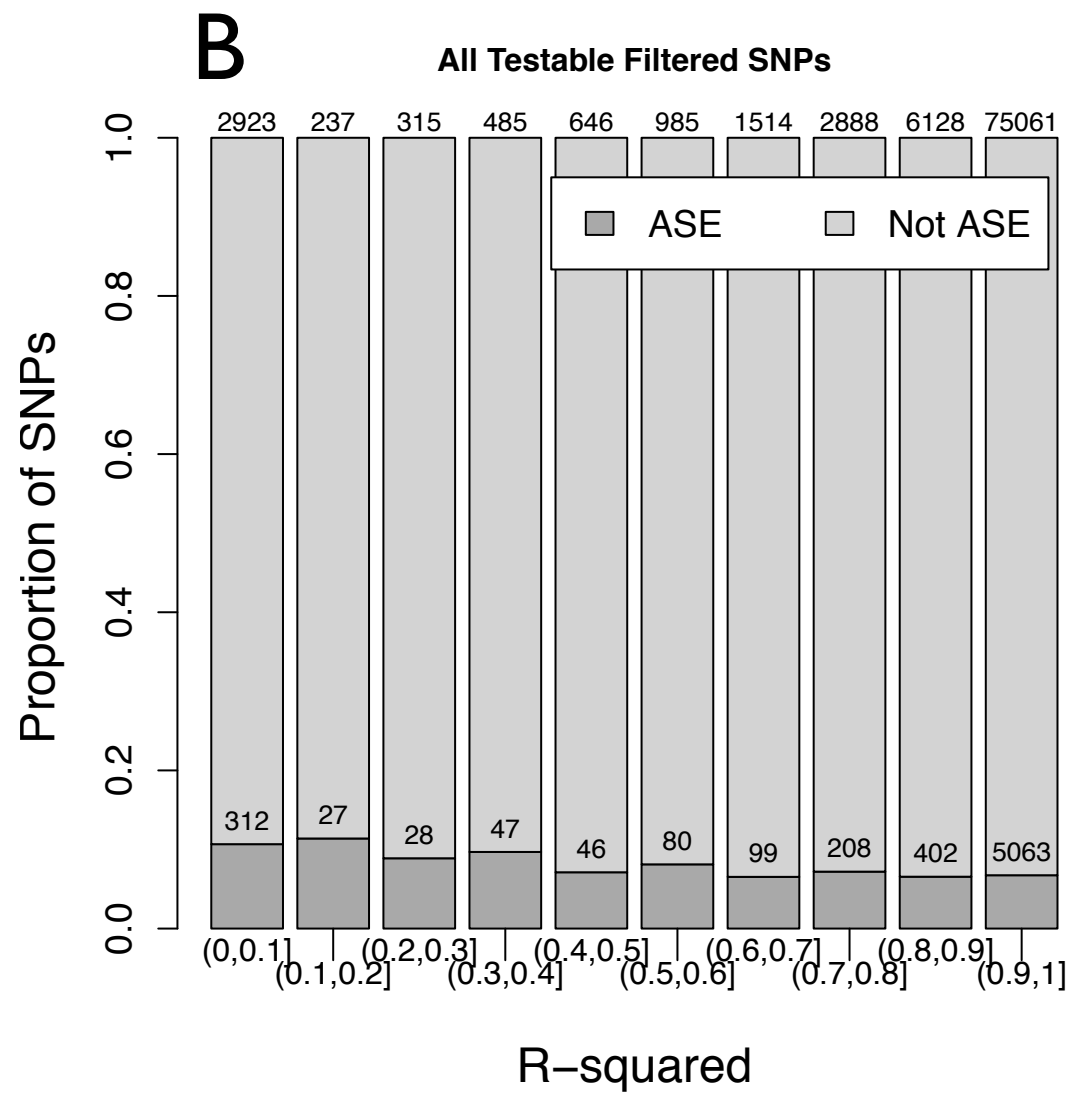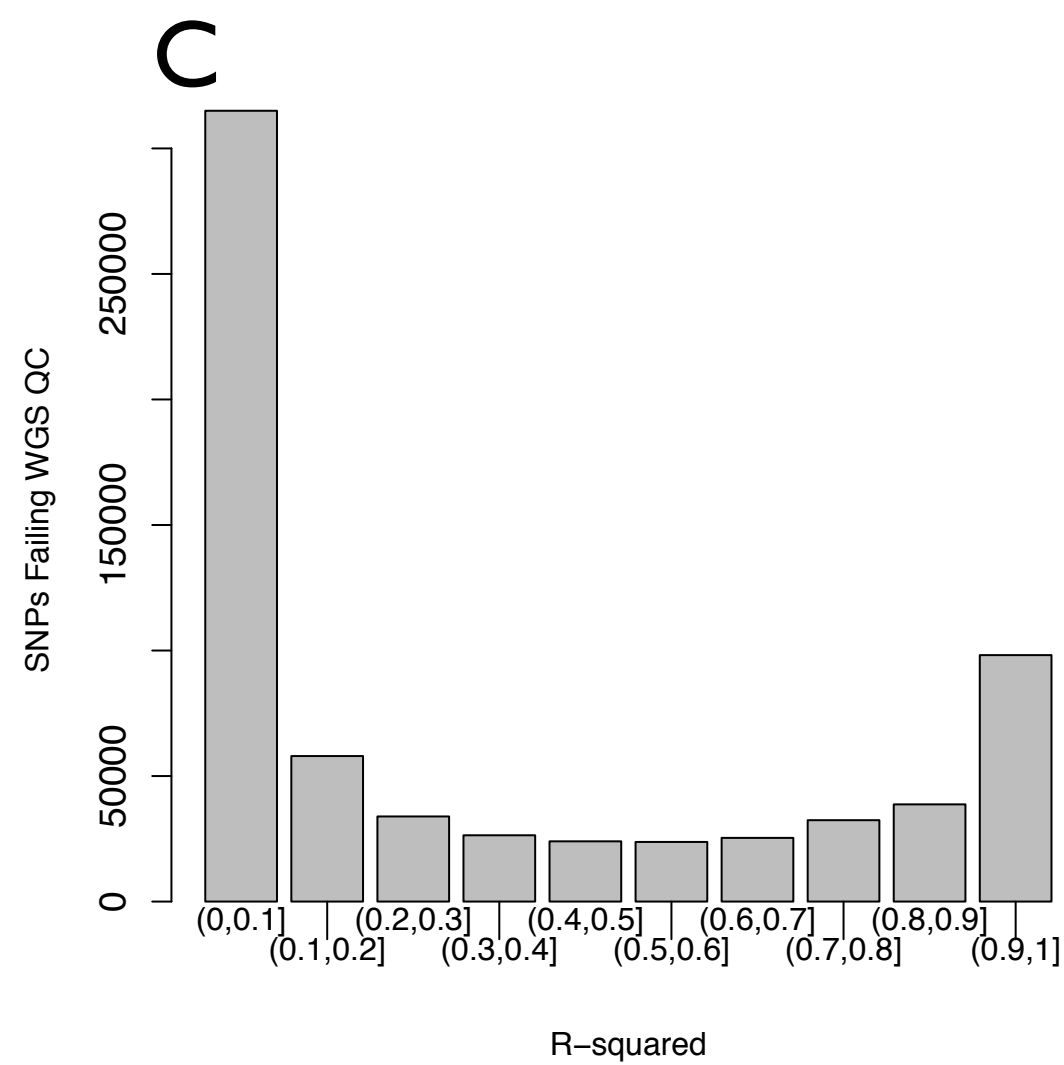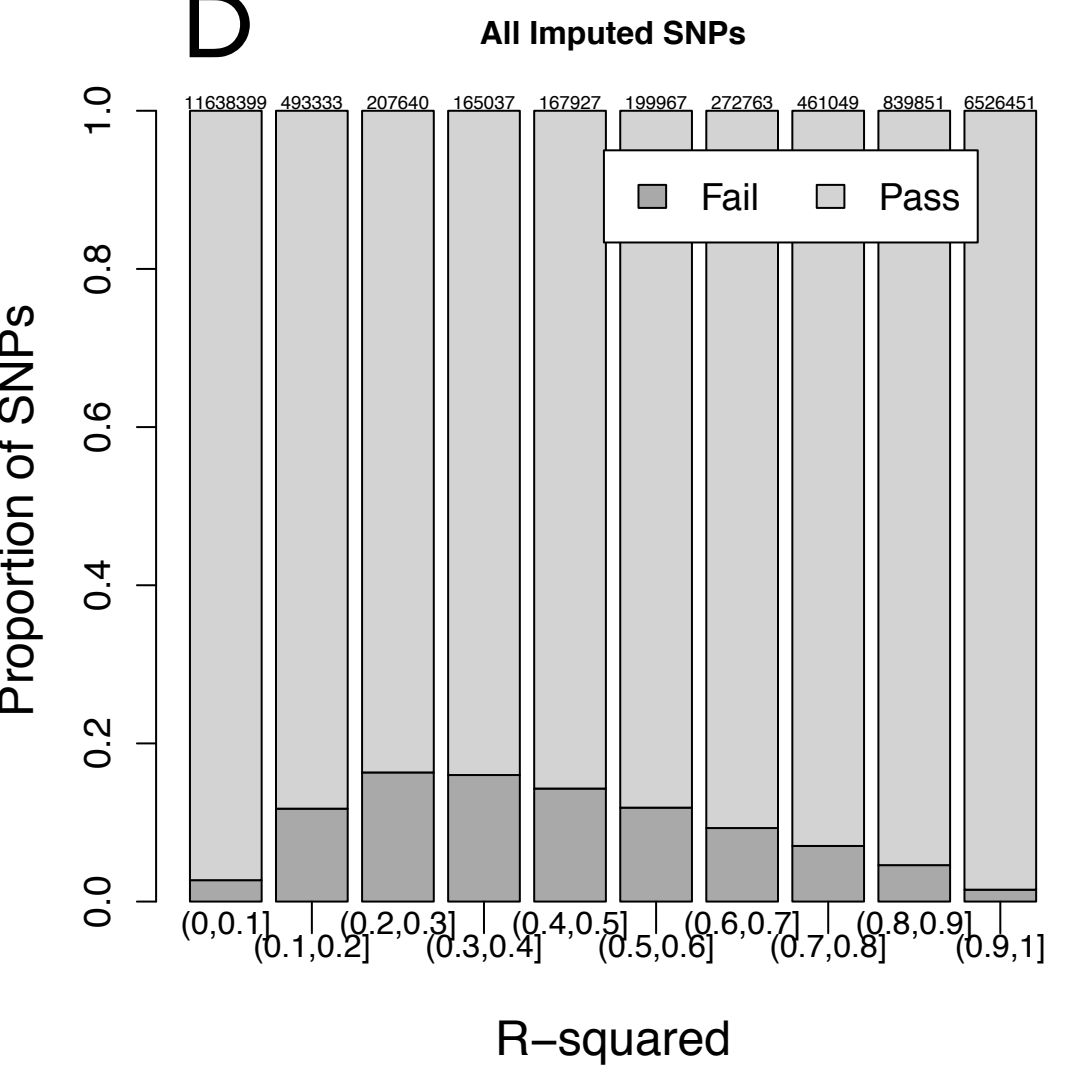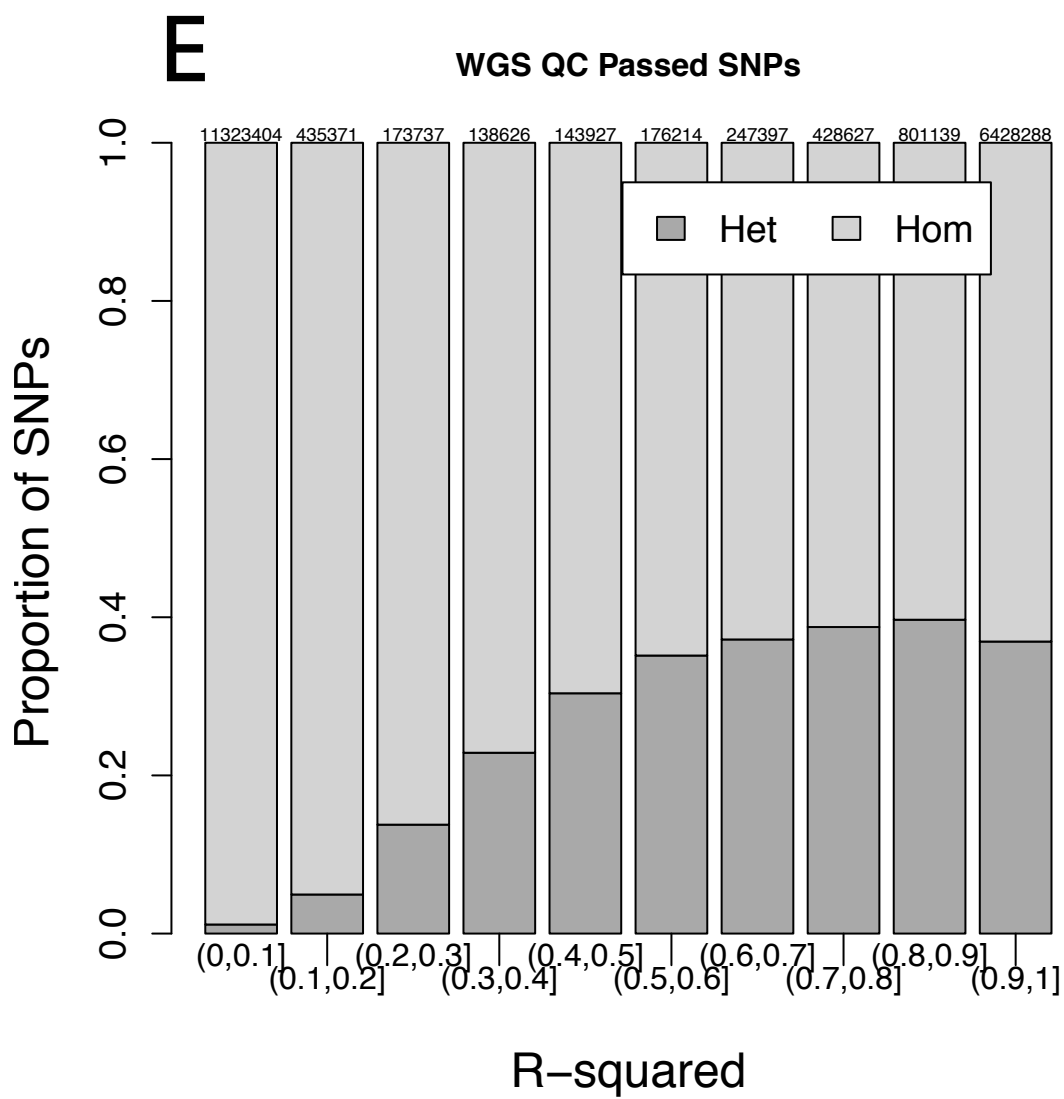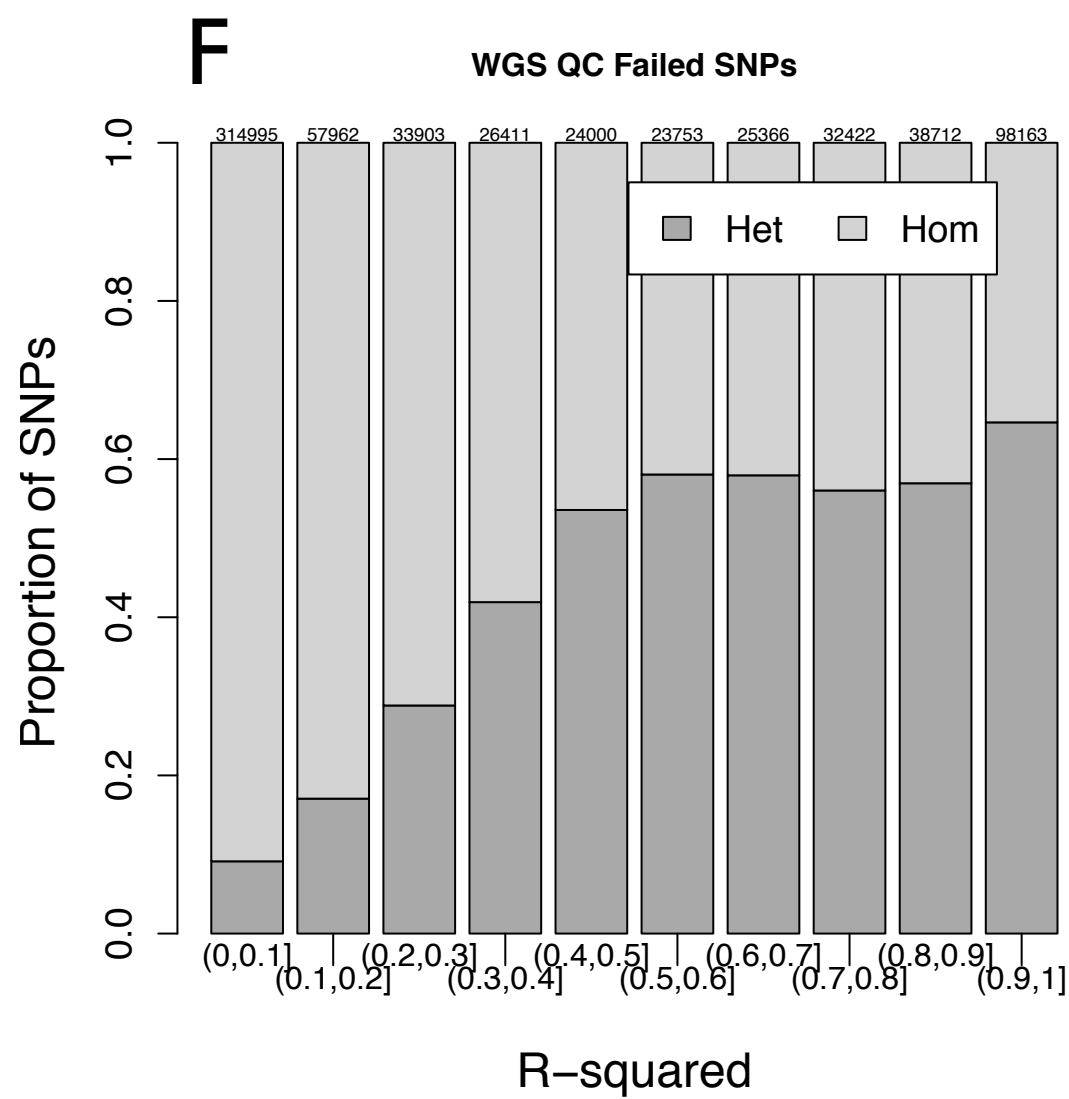

Supplement: S6 Fig — (A) Proportion of all testable SNPs classified as ASE binned by R2 values. Poorer R2 values have higher likelihood of being called ASE. (B) Proportion of all testable SNPs classified as ASE after removing SNPs with alignment confounders, indicating substantial reduction in ASE SNPs with poor R2 values. (C) Total number of SNPs failing WGS verification binned by R2 values. SNPs with lower R2 values make up the highest number of SNPs failing WGS verification, and SNPs with high R2 make up a large proportion also. (D) Proportion of all SNPs failing WGS verification, binned by R2 values. (E) Proportion of WGS verified heterozygous SNPs binned by R2 values. (F) Proportion of SNPs heterozygous SNPs failing WGS verification, binned by R2 values. (PDF) [file pone.0126911.s006.pdf]

**A** Het. WGS QC Passed SNPs

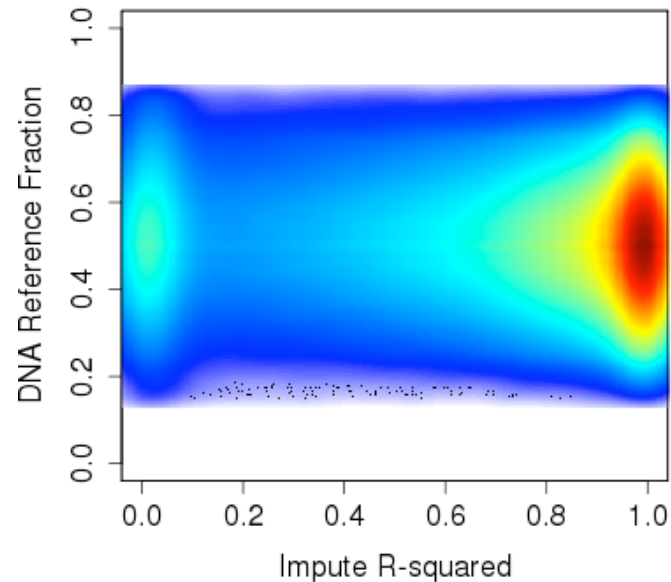

**B** Het. WGS QC Failed SNPs

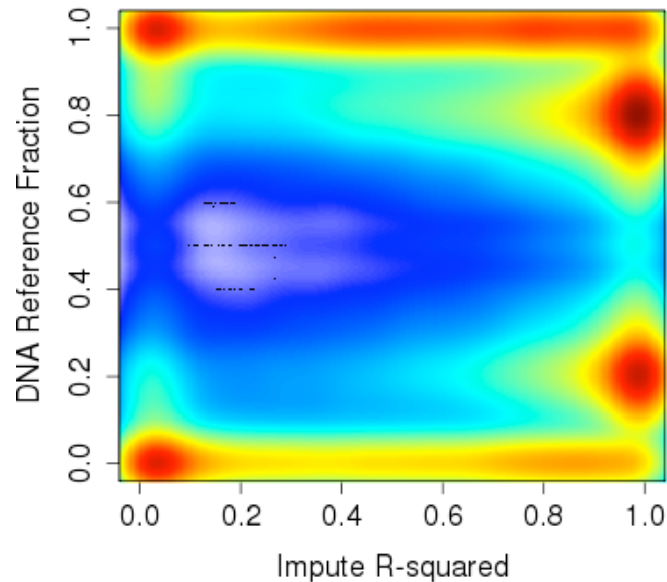

**C** All Testable SNPs

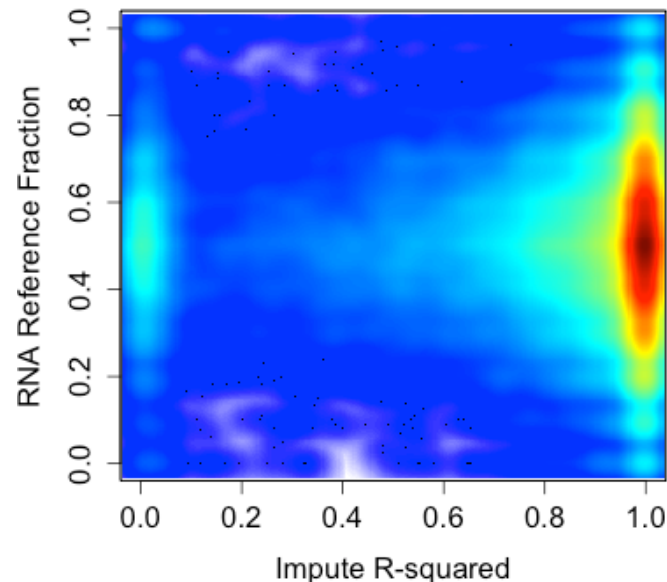

Supplement: S7 Fig — (A) DNA reference fraction plotted against imputed R2 values for all WGS verified heterozygous SNPs, indicating an average reference fraction of 0.5, consistent with true heterozygosity. (B) Smoothed scatter plot of DNA reference fraction against imputed R2 value for all SNPs imputed as heterozygous, but failing WGS verification. (C) Smoothed scatter plot of RNA reference fraction against imputed R2 values for all ASE testable SNPs (WGS verified heterozygous, and RNA-seq coverage > = 10 PCR de-duplicated reads). (PDF) [file pone.0126911.s007.pdf]

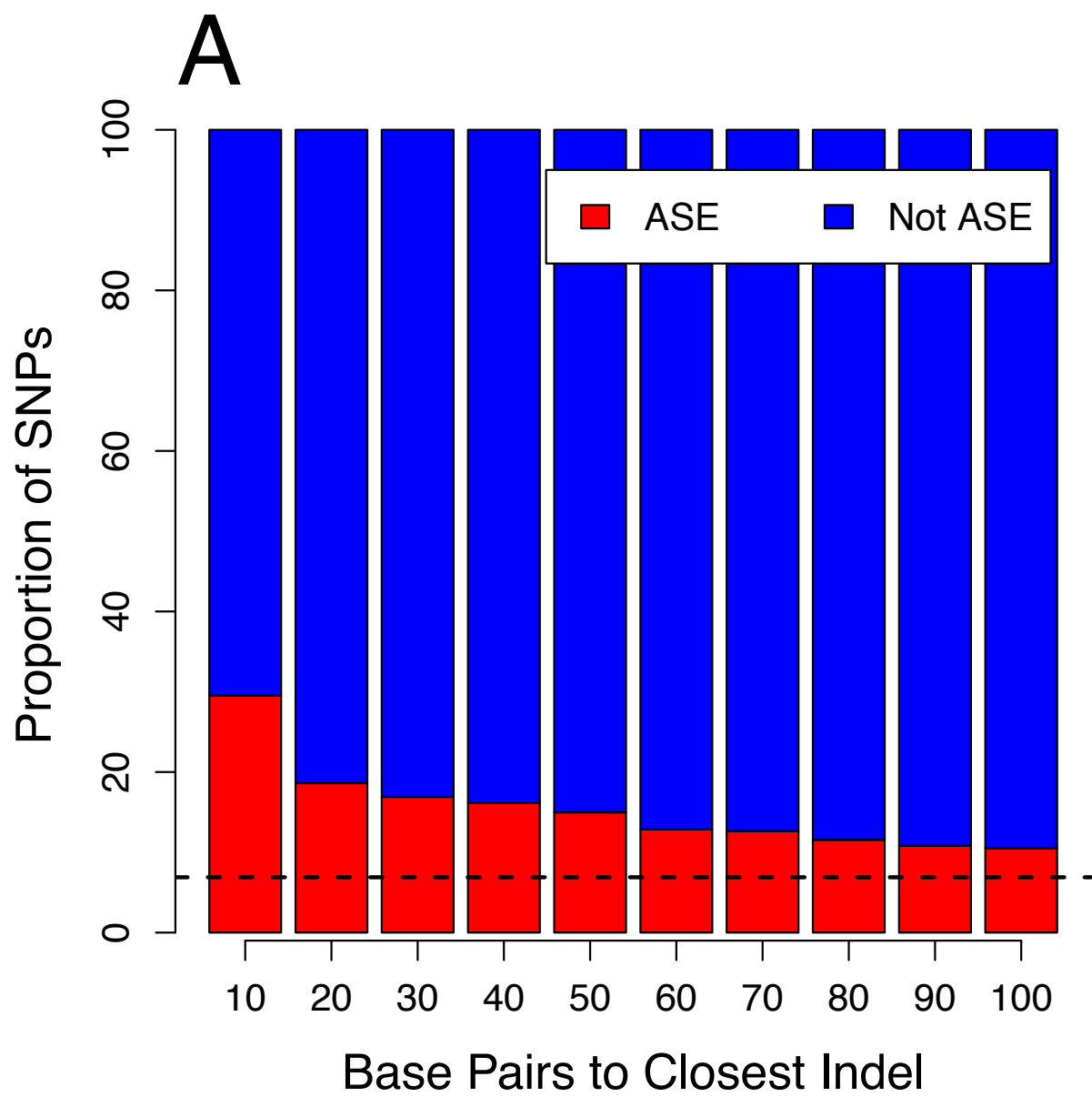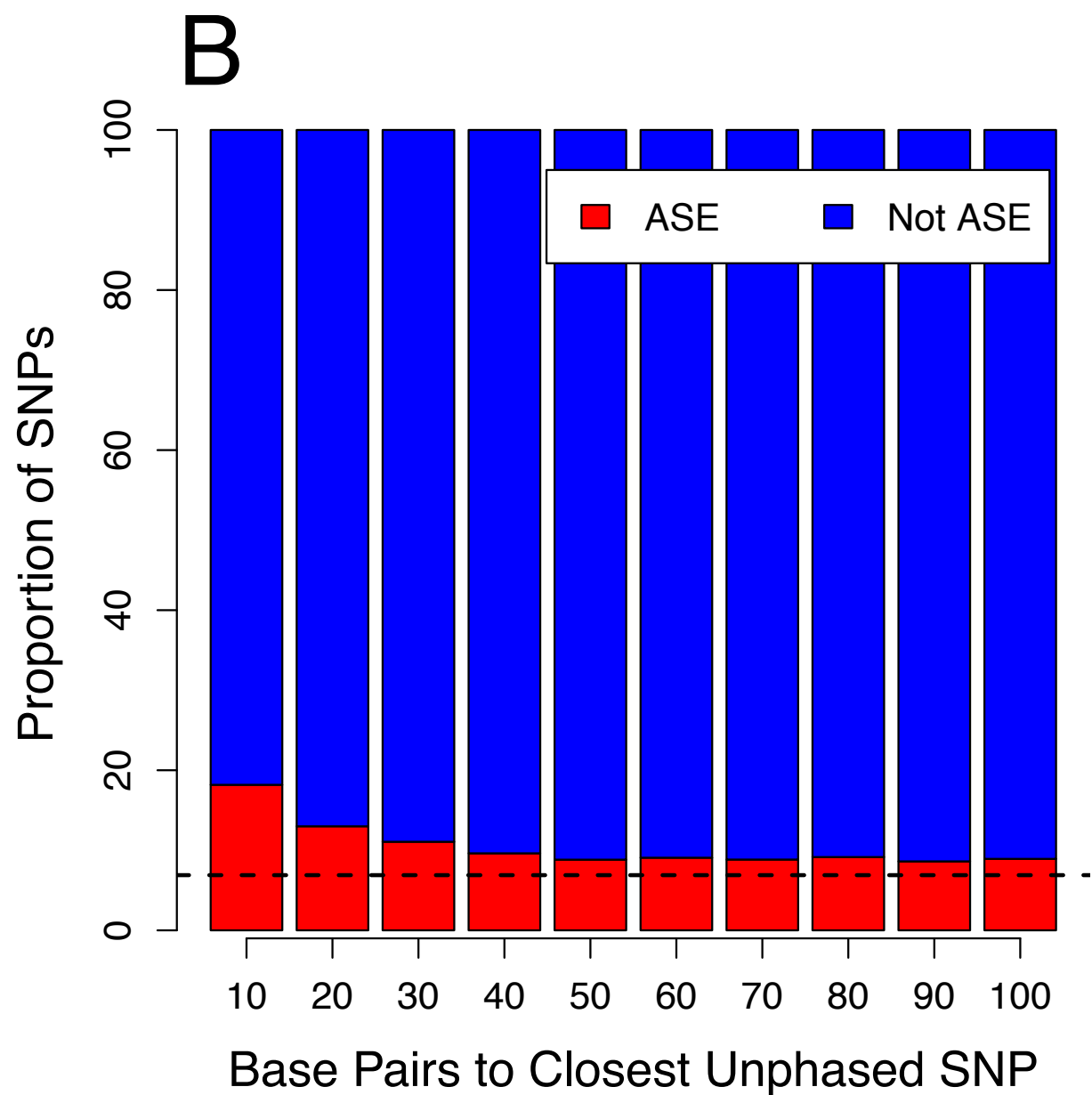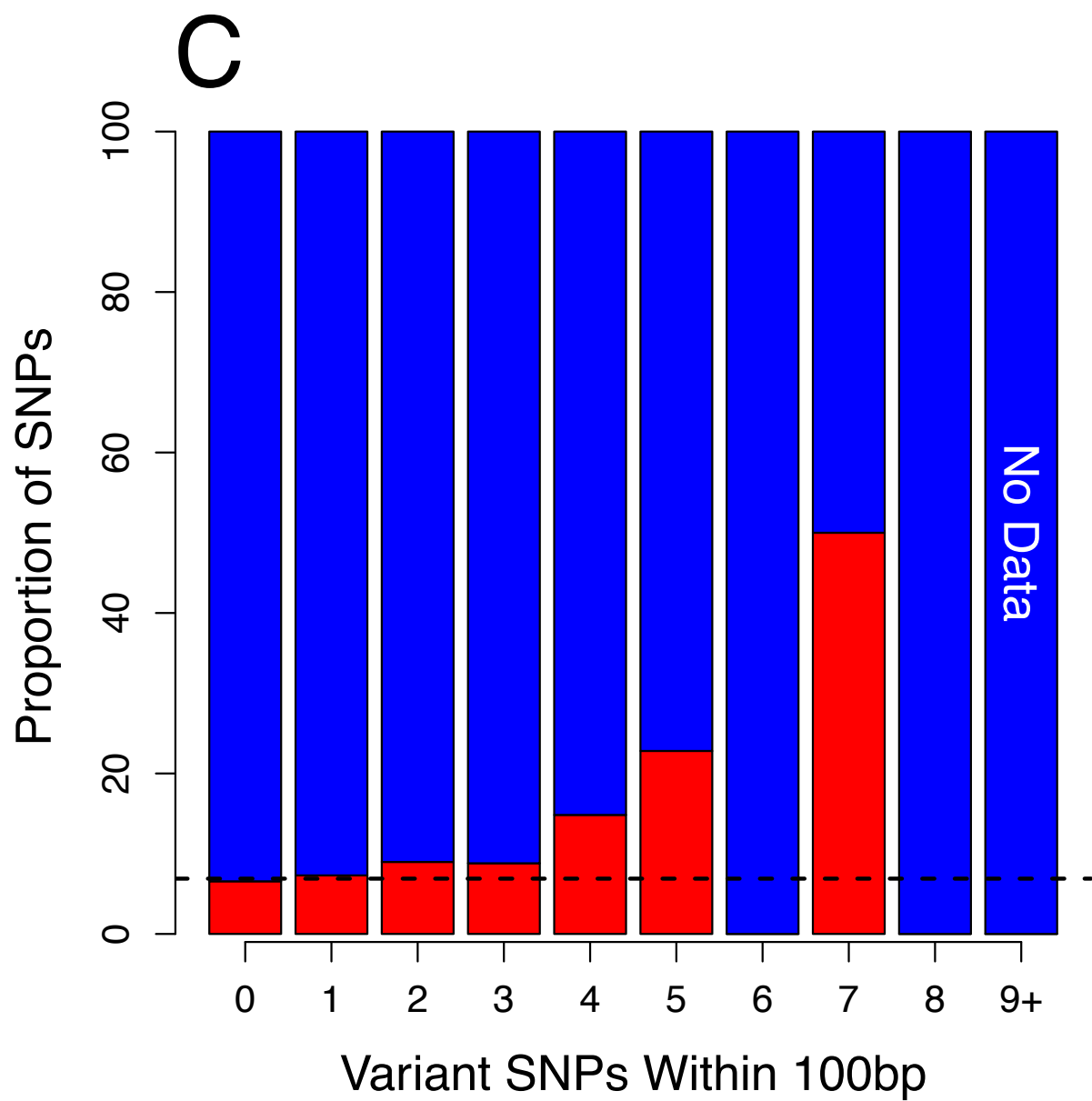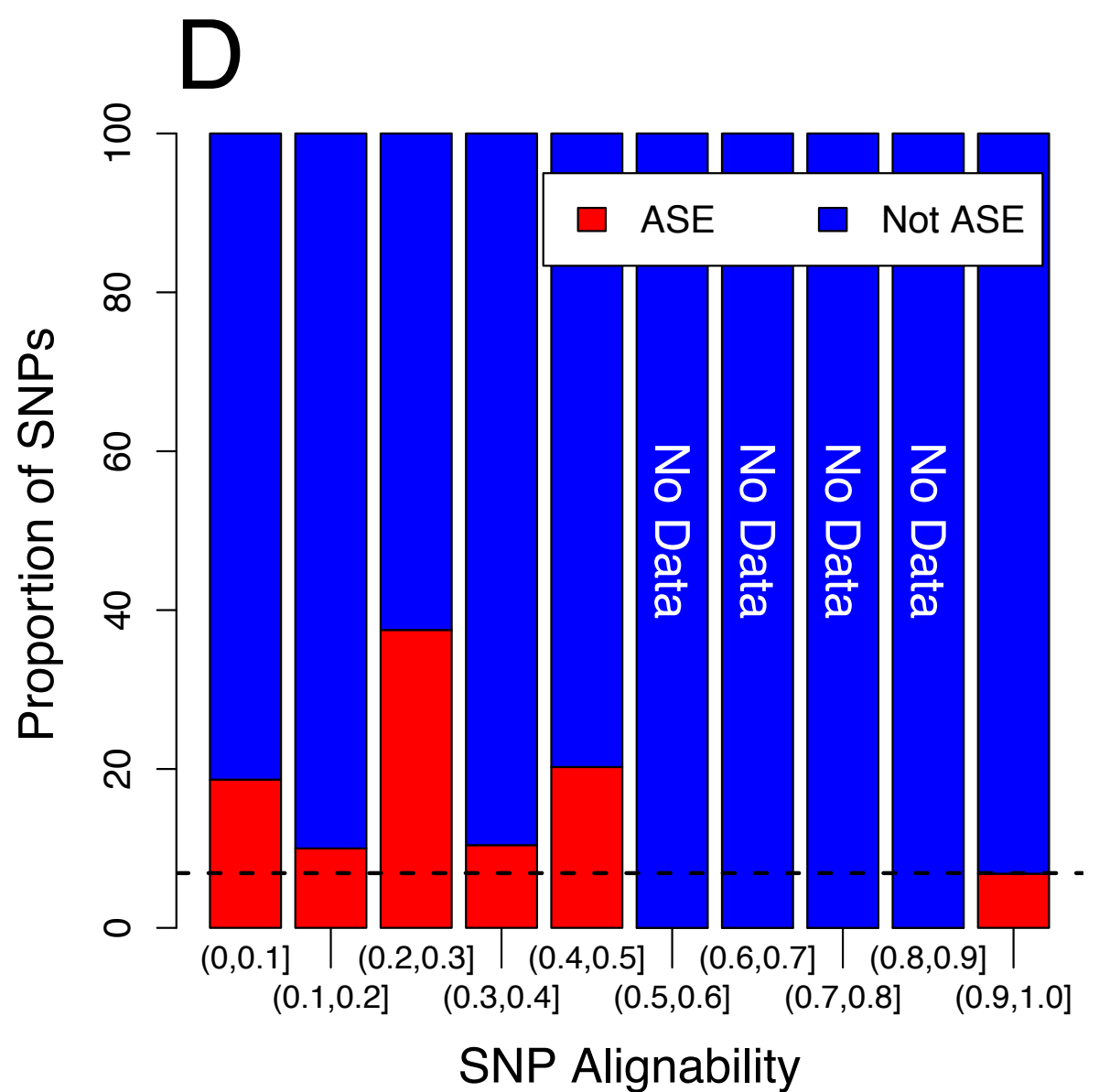

Supplement: S8 Fig — Figure is a recreation of Fig 2, excluding all SNPs with an R2 value < 1. (A) SNPs binned by genomic distance to nearest heterozygous indel. (B) SNPs binned by nearest heterozygous un-phased de novo called SNP. (C) SNPs binned by the number of proximal heterozygous SNPs. (D) SNPs binned by genomic alignability. SNPs with perfect R2 values are confounded by proximal SNVs and poor alignability. (PDF) [file pone.0126911.s008.pdf]

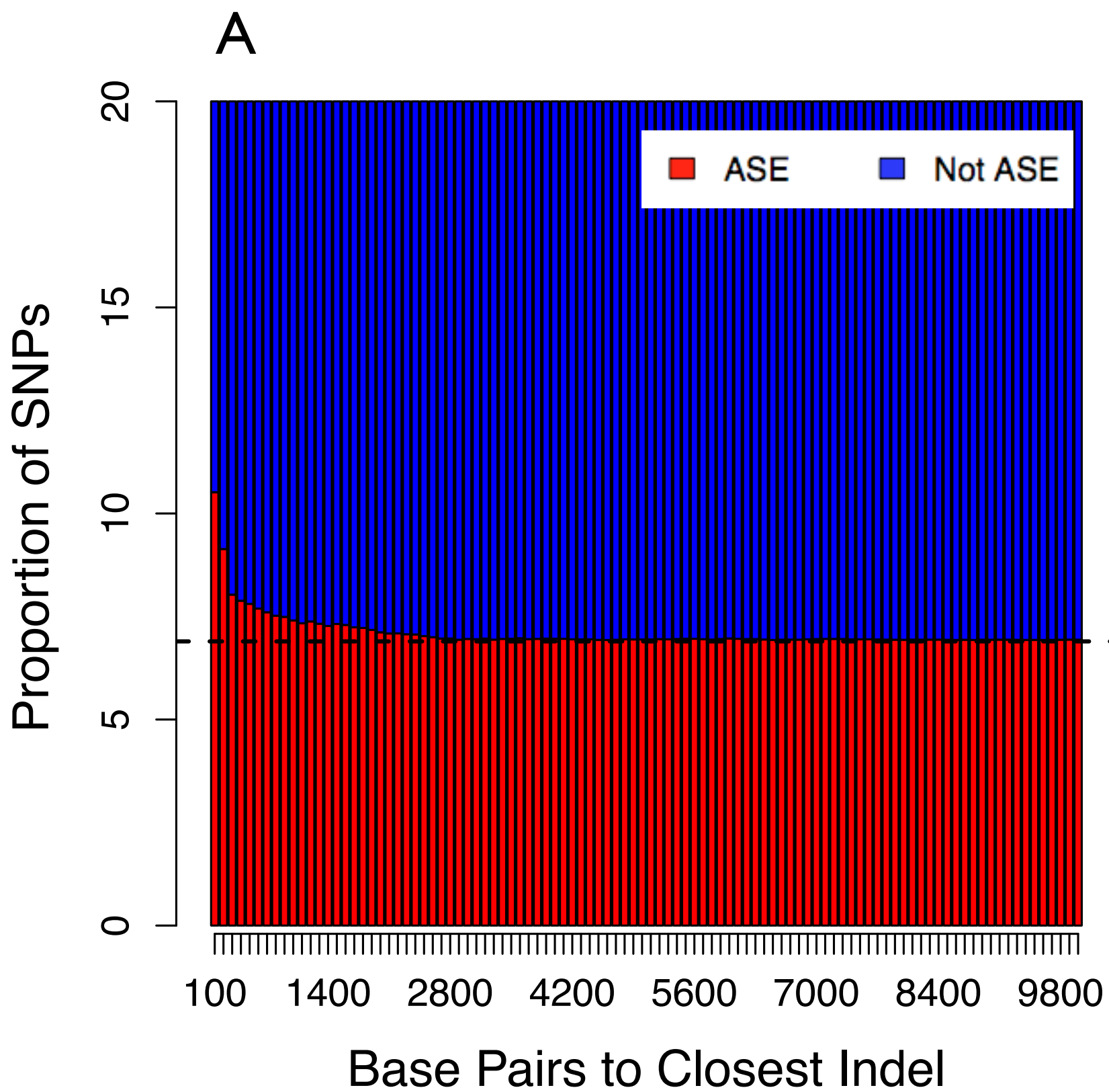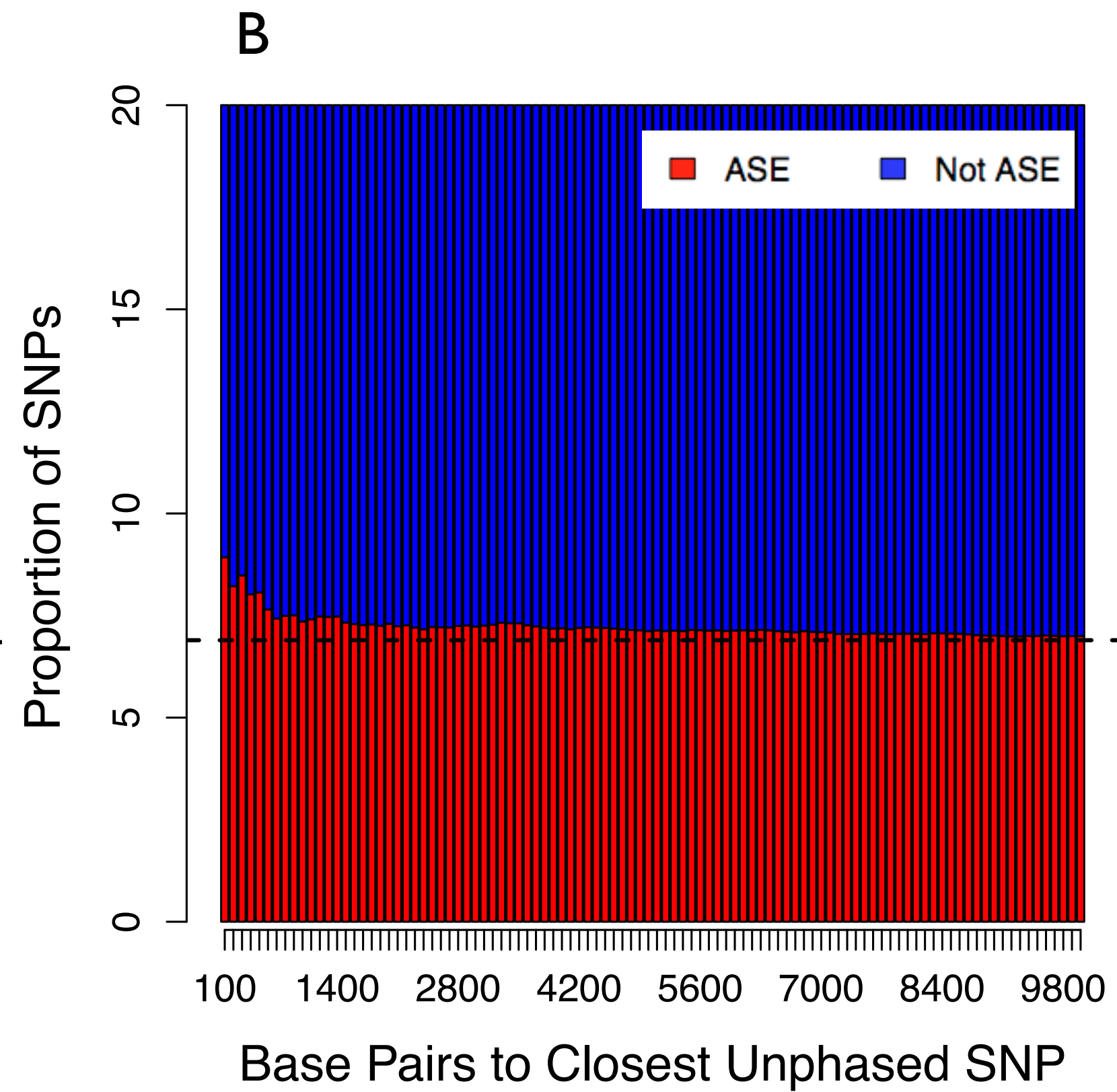

Supplement: S9 Fig — Figure is a recreation of S4 Fig, excluding all SNPs with an R2 value < 1. Increased proportion of ASE classified SNPs proximal to unphased SNPs and indels extends to a distance beyond the length of a read. Removing SNPs with R2 values < 1 slightly reduces the distance of this confounder for indels (A), and the severity of the confounder for unphased SNPs (B), but does not eliminate it completely in either situation. (PDF) [file pone.0126911.s009.pdf]

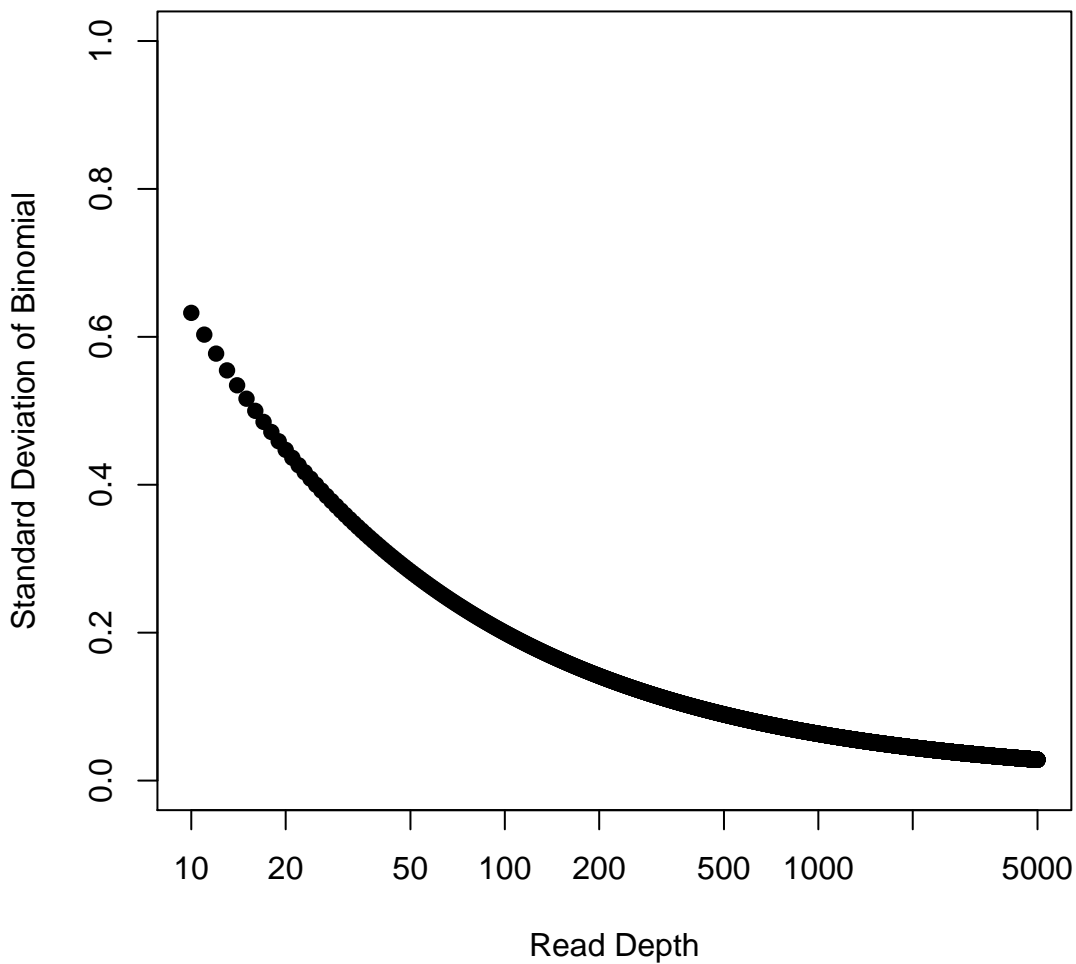

Supplement: S10 Fig — As read depth increases the standard deviation of the binomial decreases. (PDF) [file pone.0126911.s010.pdf]

## Brain

A

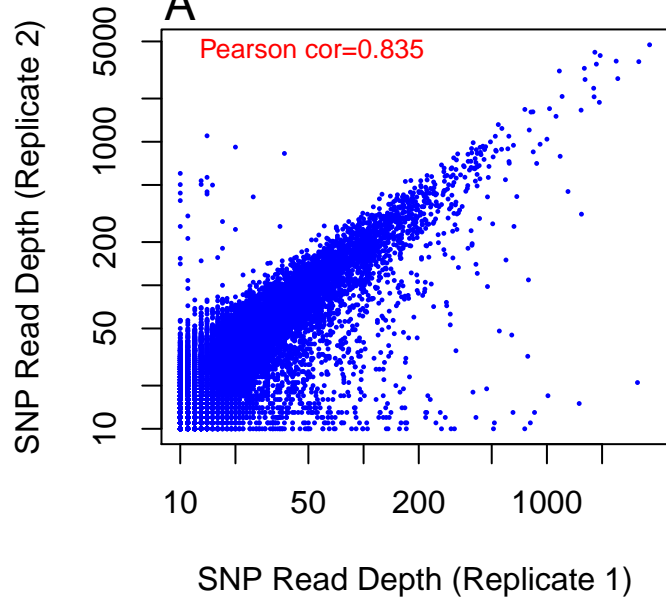

## Liver

B

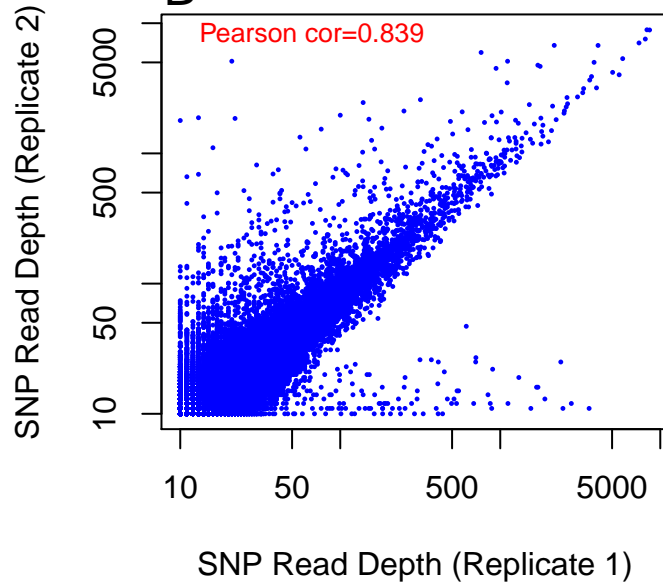

Supplement: S11 Fig — While the correlation between SNP allelic expression differed substantially, the correlation between SNP sequence depth was much stronger. (PDF) [file pone.0126911.s011.pdf]

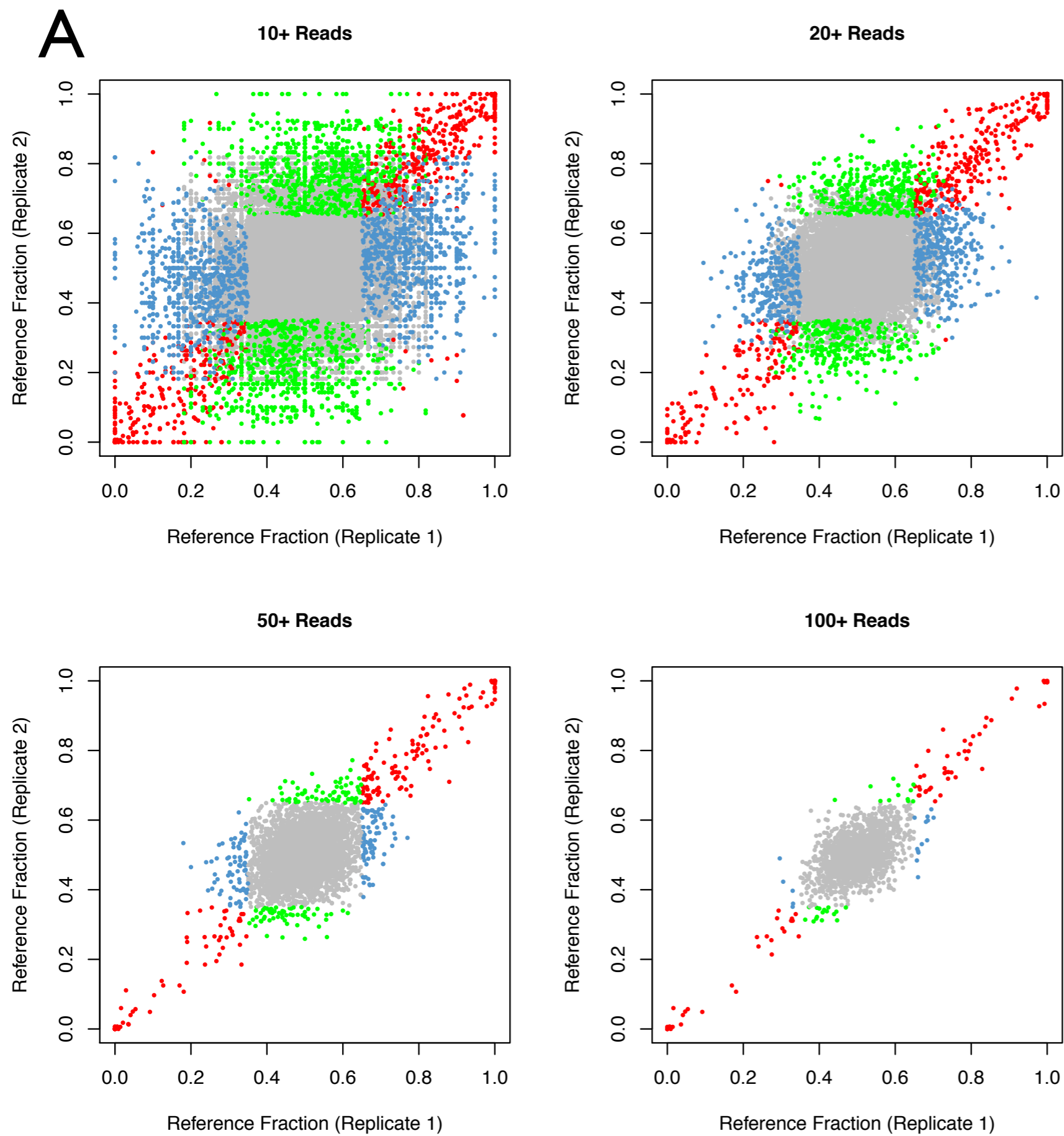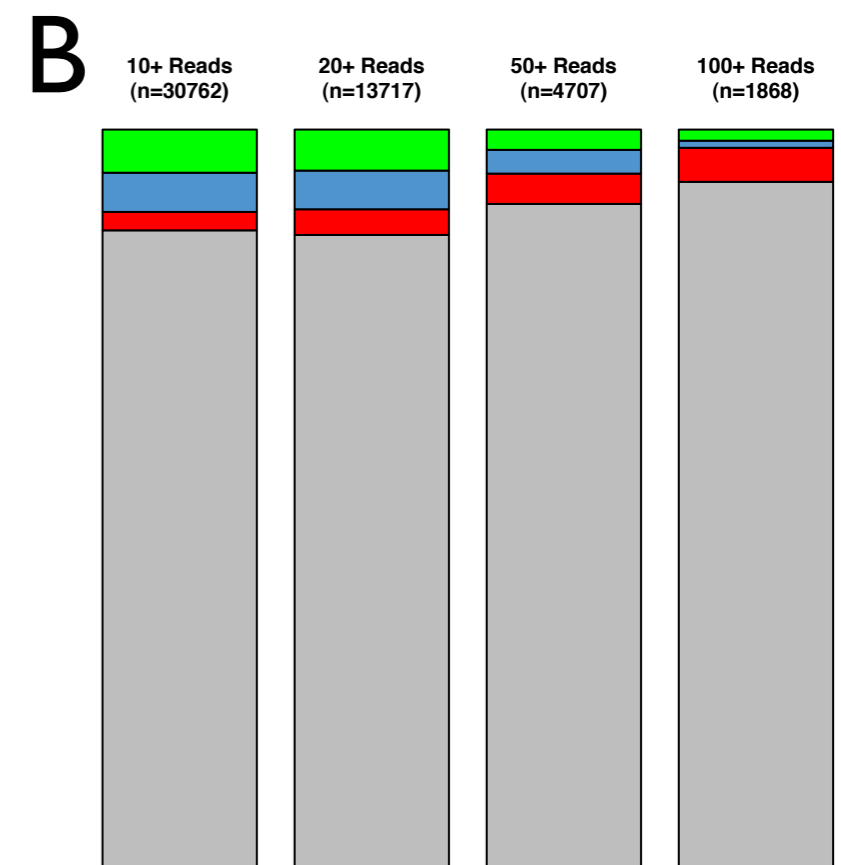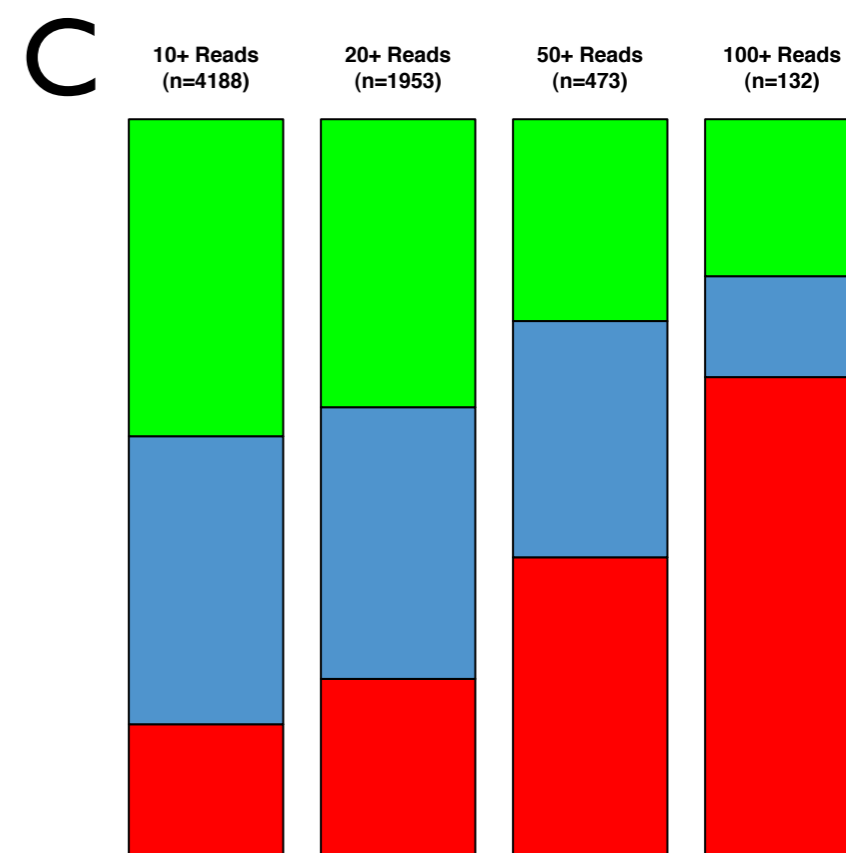

Neither ASE Both ASE Rep 1 ASE Only Rep 2 ASE Only

Supplement: S12 Fig — (A) Scatter plots for correlations show that as read depth increases, so too does correlation. (B) Bar plots show the proportion of all SNPs classified as either not ASE in both replicates, ASE in both replicates, or ASE in one or the other replicate. (C) Bar plots show proportion of SNPs classified as ASE in any replicate as read depth increases. Increasing the read depth dramatically improves correlation, but at a large cost of number of testable SNPs, and hence power to detect ASE. (PDF) [file pone.0126911.s012.pdf]

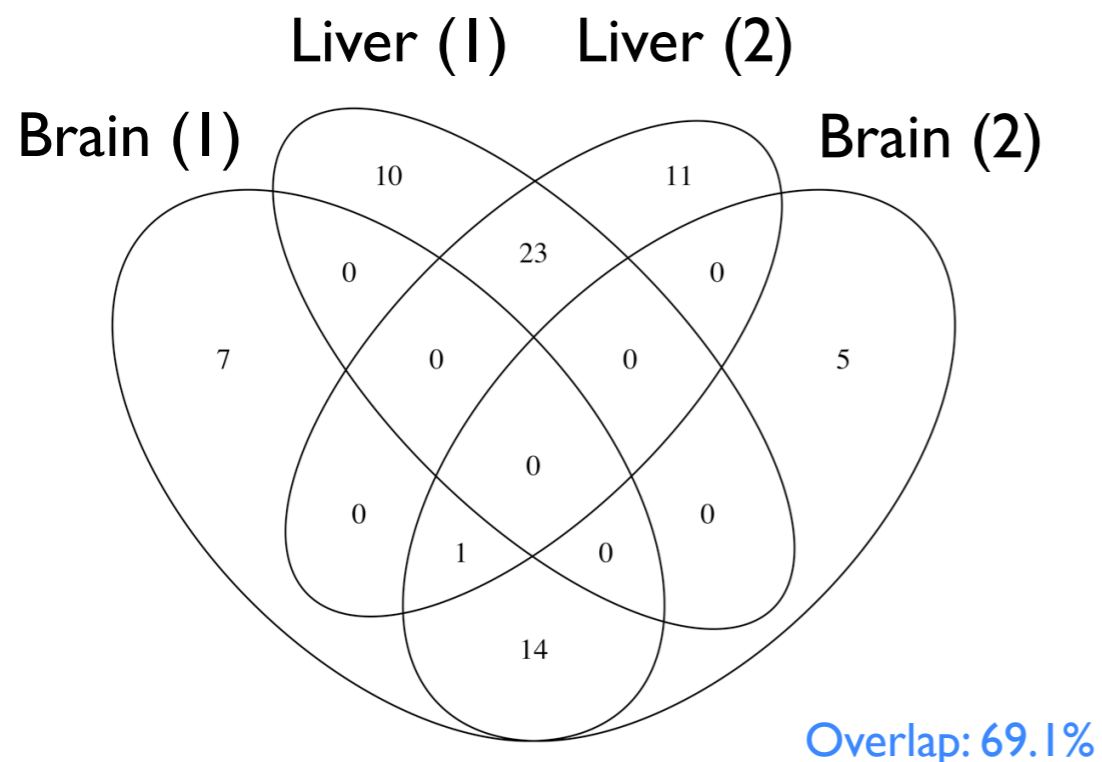

2+ Testable, 1+ ASE, In phase

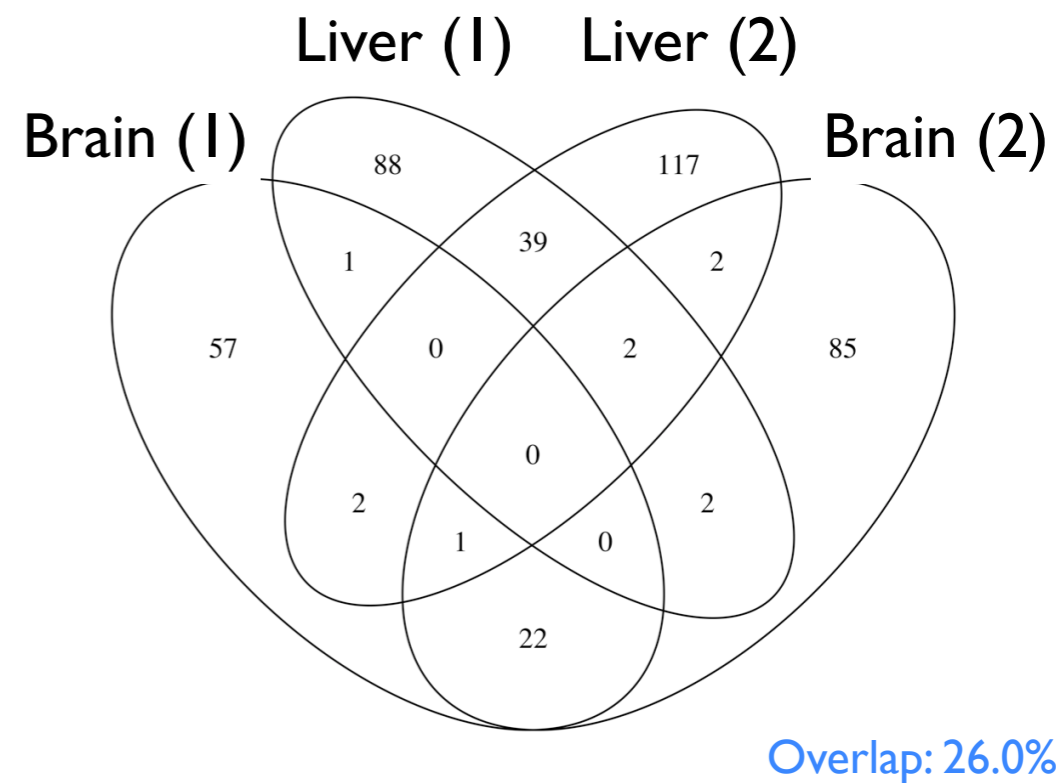

2+ Testable, 0+ ASE, In phase

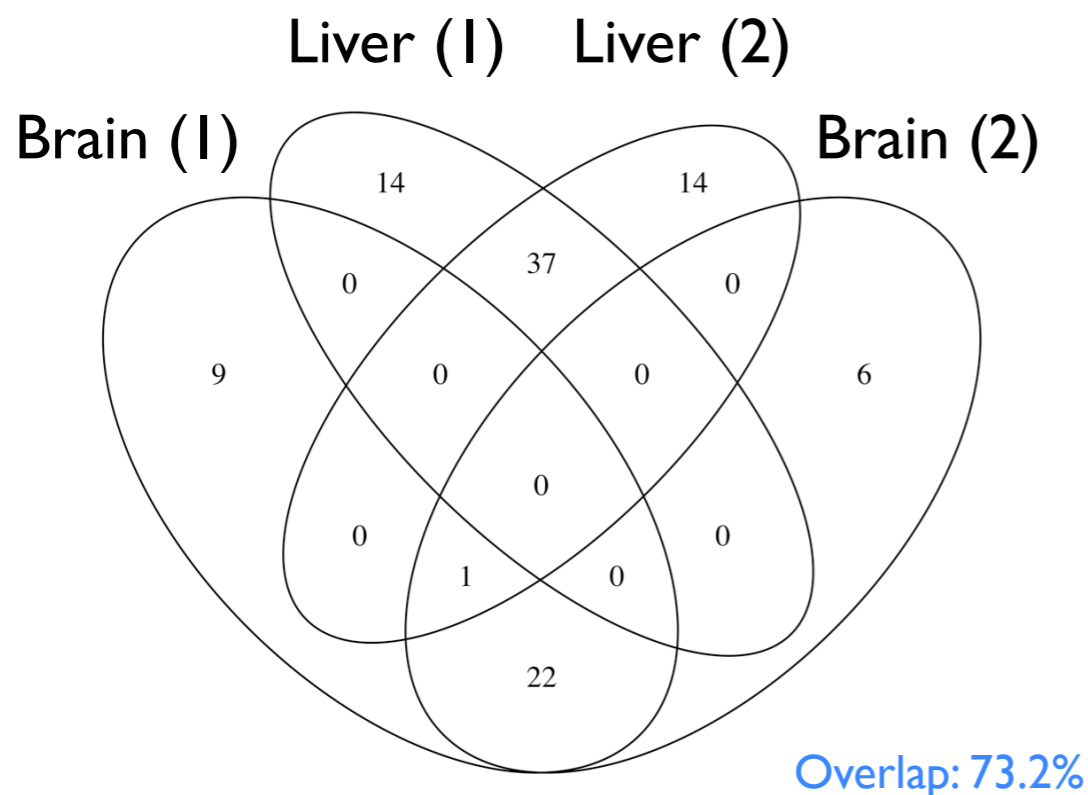

2+ Testable, 1+ ASE, Any phase

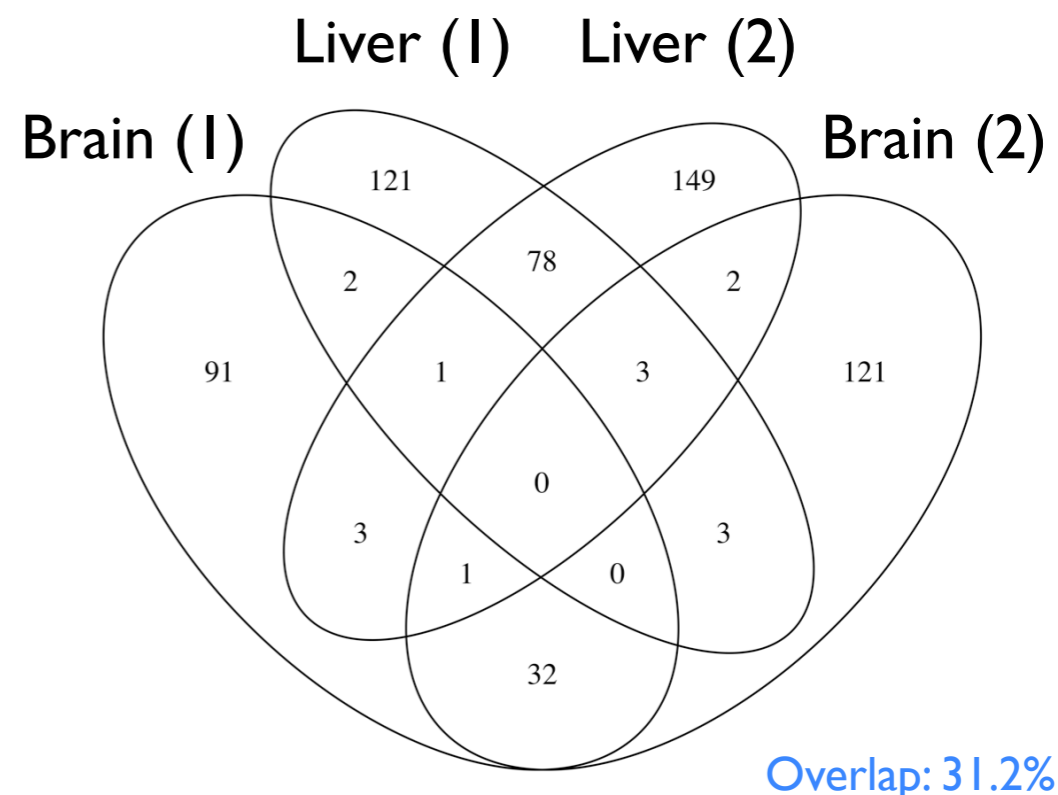

2+ Testable, 0+ ASE Any phase

Supplement: S13 Fig — In all plots, genes are only tested for ASE if they contain at least two testable SNPs. (A) Very strict testing is achieved by classifying a gene as ASE if it contains at least one replicated ASE SNP and all SNPs within the gene show direction of imbalance concordant with the haplotype or (C) any direction. (B) Less stringent testing is shown for genes when they can contain SNPs of which none are classified as ASE (but may still be slightly imbalanced), but are still concordant in imbalance direction, or (D) with any direction of imbalance. The most optimal parameters to maximize specificity, sensitivity and overlap between replicates are shown in panel (C). (PDF) [file pone.0126911.s013.pdf]

# All SNPs Testable in Both Replicates

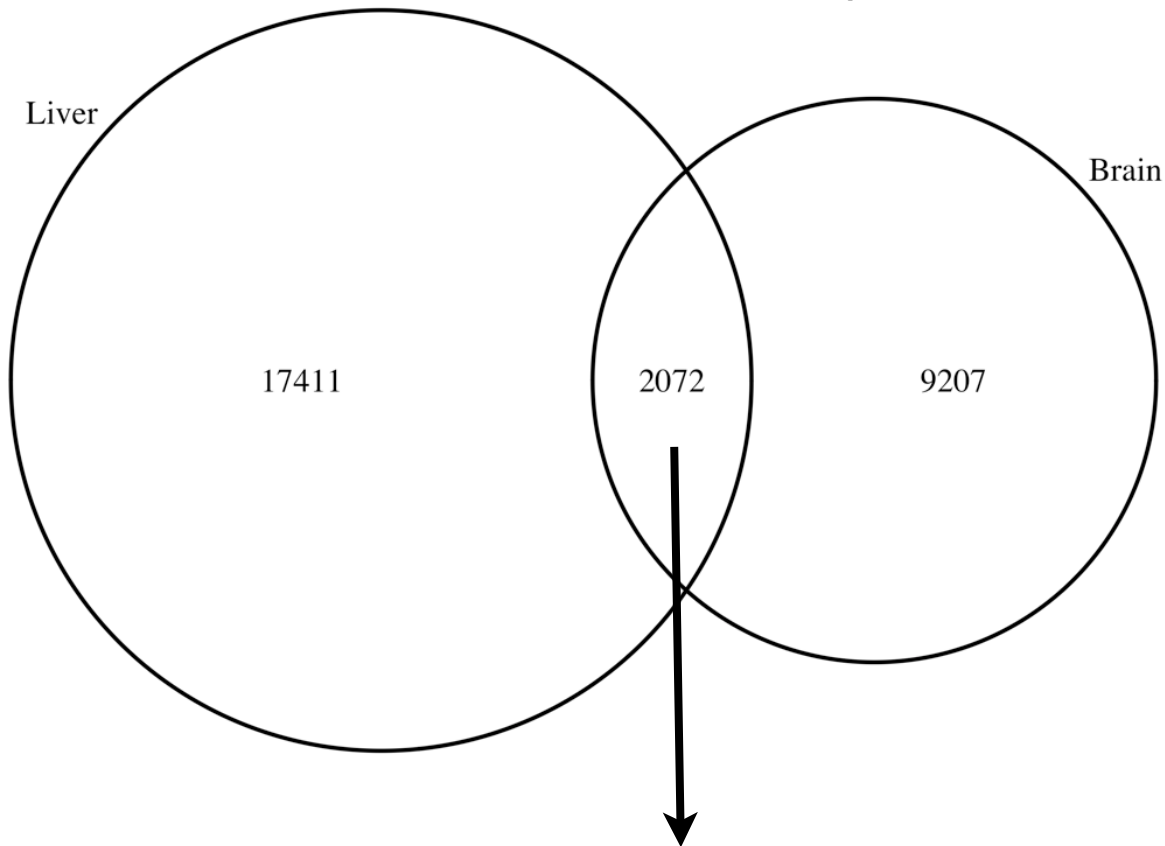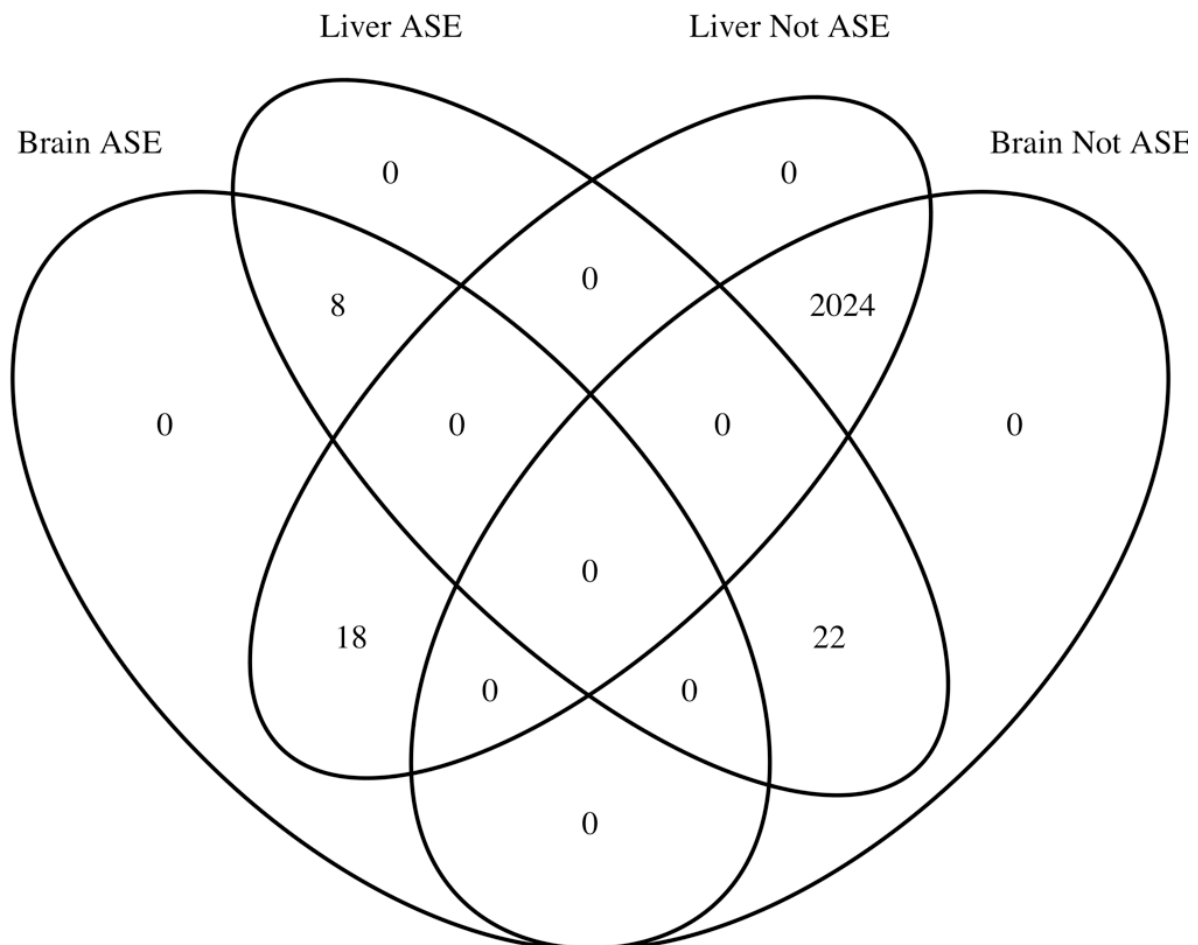

# All SNPs Testable In Both Replicates and Both Tissues

Supplement: S14 Fig — Of all SNPs testable in both replicates, less than 13.47% were testable in both tissues (top). Of these SNPs, a tiny percentage (0.39%; n = 8), were classified as ASE in both tissues (bottom). (PDF) [file pone.0126911.s014.pdf]

A

**ERAP2, Length:43777, Strand:+**

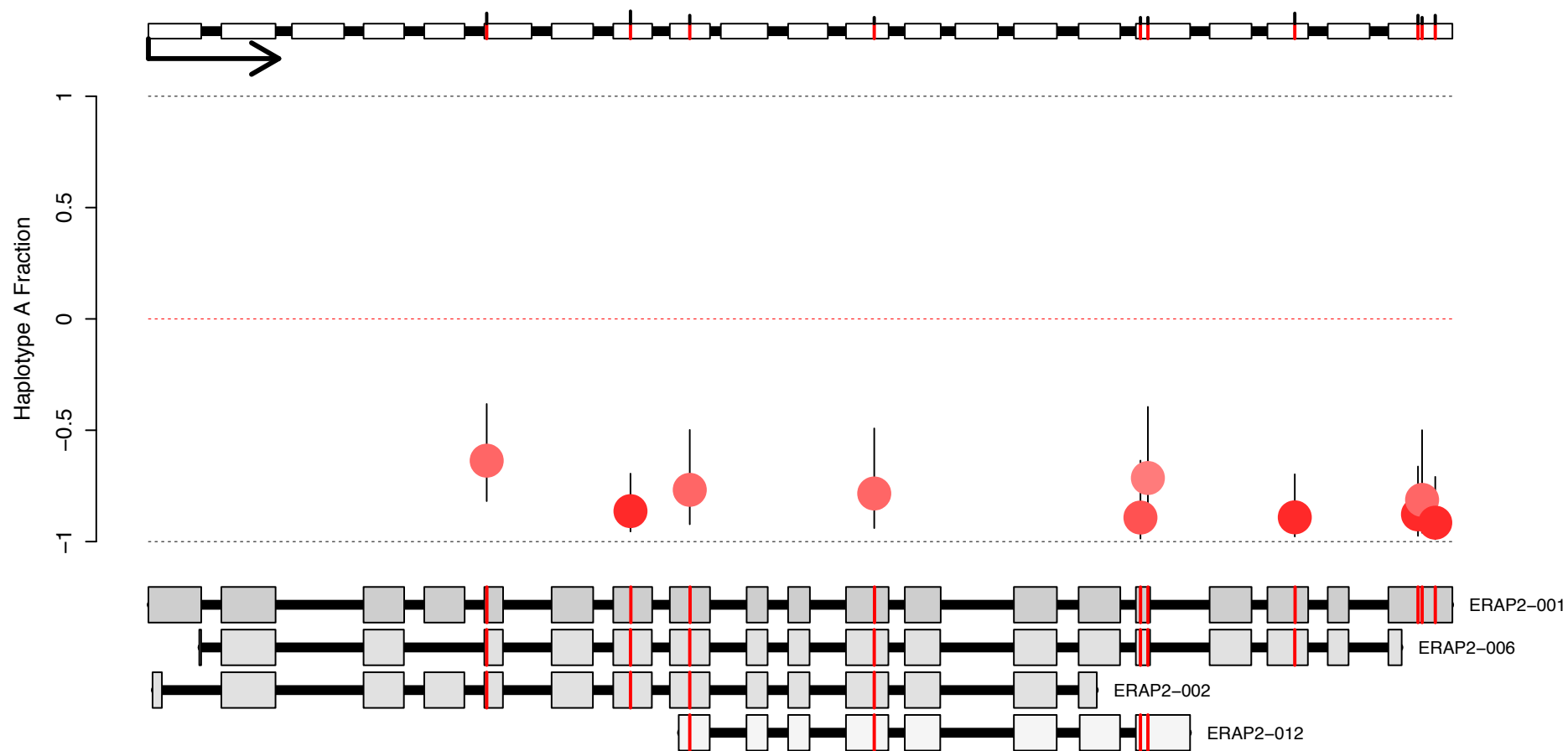

**B**

**GSTA2, Length:13470, Strand:-**

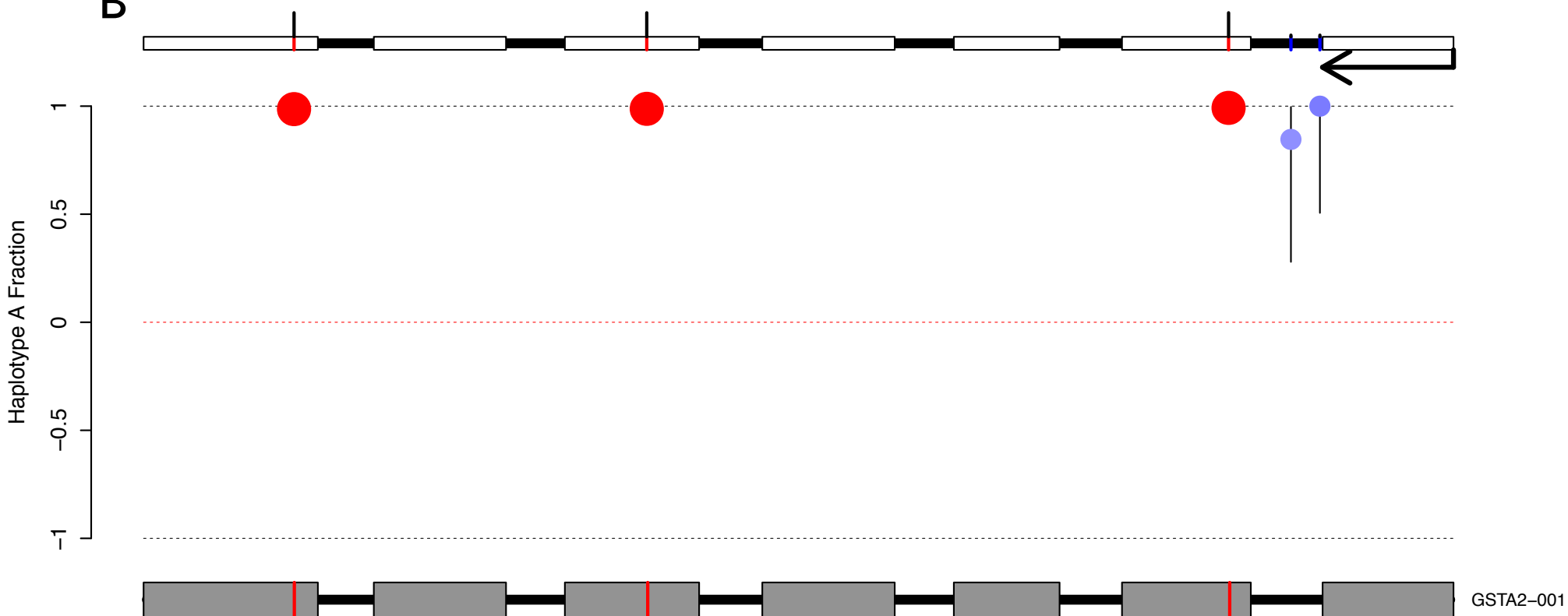

Supplement: S15 Fig — Red circles are exonic SNPs and blue circles intronic SNPs. SNP colour is proportional to probability of ASE (darker is more significant). Error bars represent the 95% binomial confidence interval (Pearson-Klopper). The gene model at the top shows the exonic (red) and intronic (blue) SNPs, the black sticks above these represent the read depth (min height represents 10 reads, max height 200 or more reads). Isoform models below are ordered by expression (top highest), and coloured by expression (darker is higher expressed). Only exonic SNPs are shown on transcripts, and only transcripts with at least one testable exonic SNP are drawn. Exon lengths are drawn at log2, and intron lengths at log10. (PDF) [file pone.0126911.s015.pdf]

Proportion of ASE SNPs

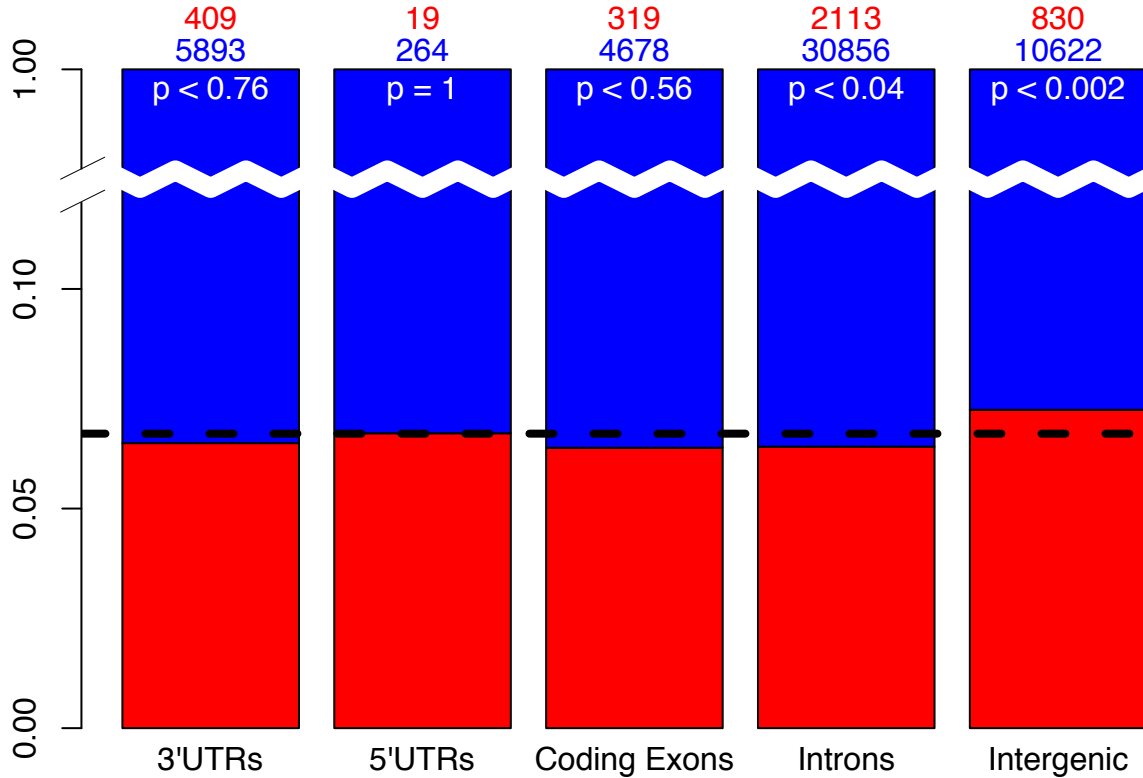

ASE

Not ASE

Supplement: S16 Fig — Dashed black line represents the average proportion of all ASE SNPs. SNPs falling within intergenic regions (p < 0.002) are more likely to be classified ASE than SNPs falling in other regions. (PDF) [file pone.0126911.s016.pdf]

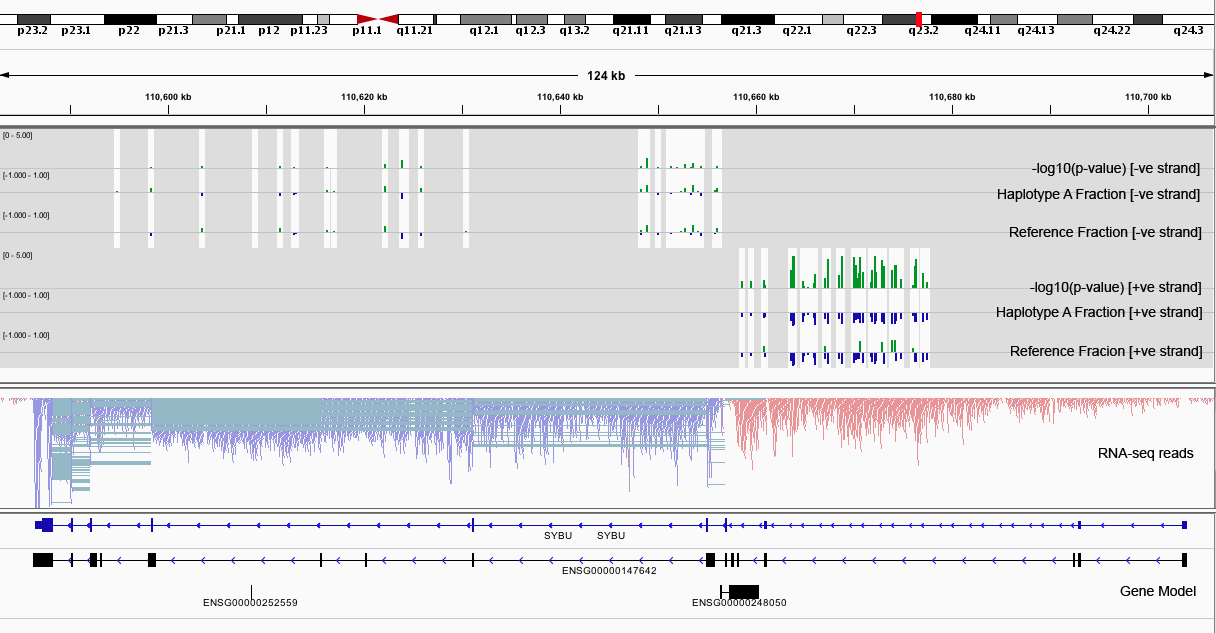

Supplement: S17 Fig — Integrative Genome Viewer (IGV [55]) screenshot where SNPs are represented as white columns within the top six gray tracks. Three tracks for each SNP with sufficient strand-specific expression are shown; the—log10 of binomial probability, reference fraction and haplotype A fraction. These are clustered into two groups, negative strand (upper three) and positive strand (lower three). RNA-seq data are shown below these tracks (red reads are transcribed from the positive strand, and blue from the negative). Gene models below the RNA-seq show gene start, end, and exon structure. A large block of 36 SNPs is observed on the positive strand showing concordant direction of allelic expression imbalance, initiating antisense to SYBU at the RP11-422N16.3 locus and running downstream for approximately 50kb. This extended un-annotated transcription, observed in the liver sample, is likely to show allelic expression imbalance along the entire transcript, however only the window of 14kb contains heterozygous SNPs and is testable. (TIF) [file pone.0126911.s017.tif]

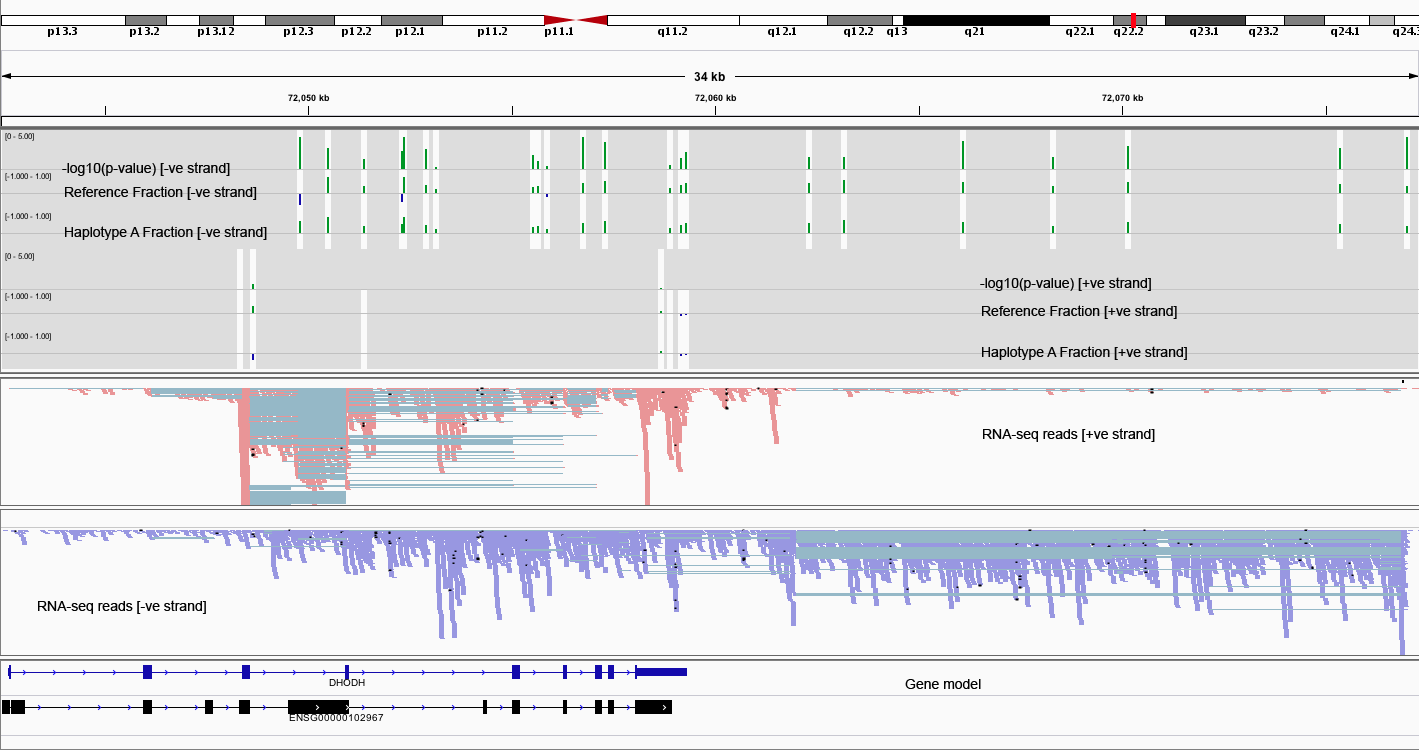

Supplement: S18 Fig — Integrative Genome Viewer (IGV [55]) screenshot where SNPs are represented as white columns within the top six gray tracks. Three tracks for each SNP with sufficient strand-specific expression are shown; the—log10 of binomial probability, reference fraction and haplotype A fraction. These are clustered into two groups, negative strand (upper three) and positive strand (lower three). RNA-seq data are shown below these tracks (red reads are transcribed from the positive strand, and blue from the negative). Gene models below the RNA-seq show gene start, end, and exon structure. 22 SNPs show strong evidence of ASE on the negative strand (top tracks). This allele-specific expression initiates downstream of and antisense to DHODH, and proceeds for a distance of approximately 32kb. This un-annotated antisense transcription also displays evidence of splicing indicating post-transcriptional regulation. (TIF) [file pone.0126911.s018.tif]

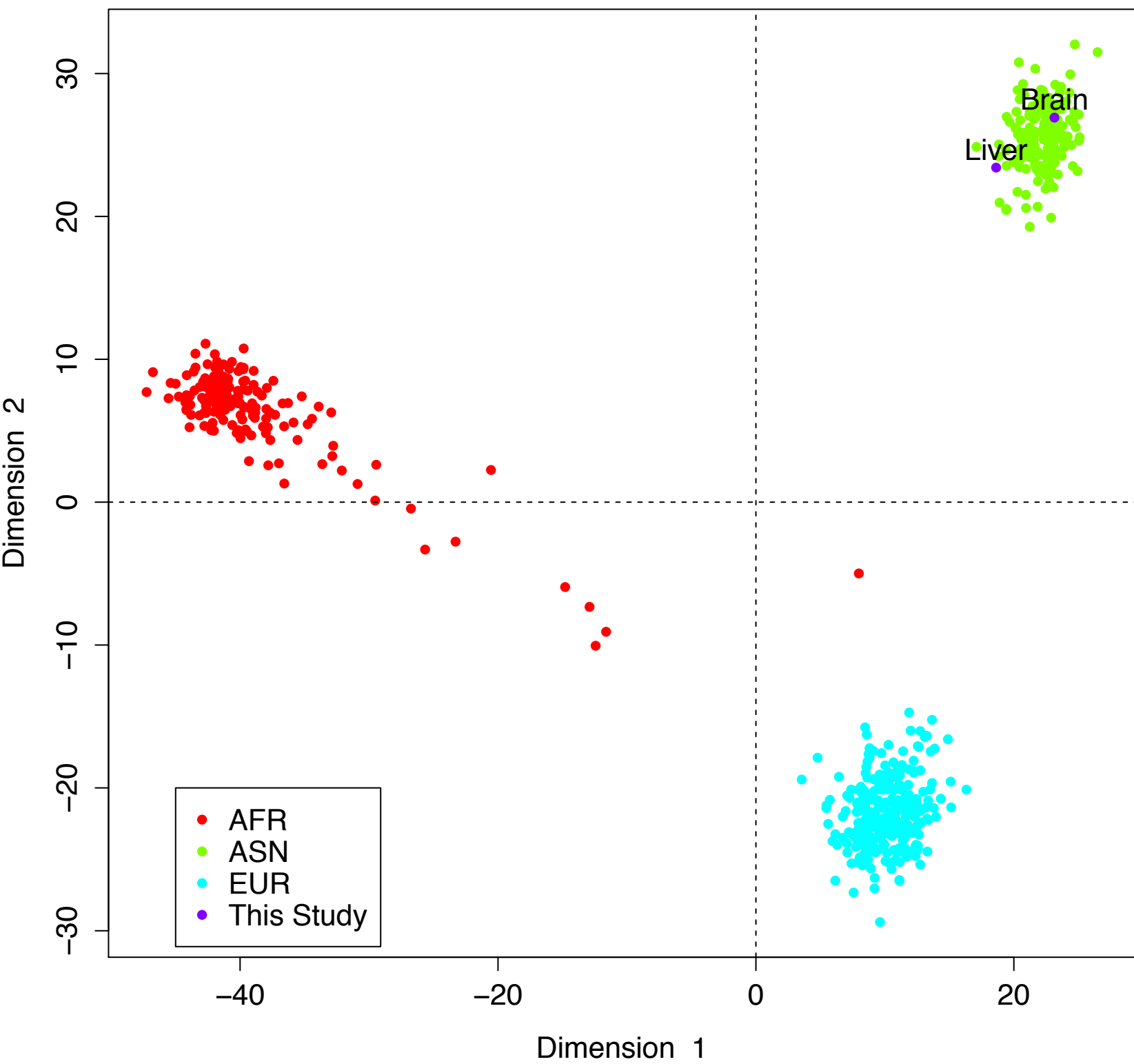

Supplement: S19 Fig — The two samples used in this study cluster with the Asian samples, indicating they are of Asian descent. (PDF) [file pone.0126911.s019.pdf]
